# Exploiting redundancy in large materials datasets for efficient machine learning with less data

**DOI:** 10.1038/s41467-023-42992-y

**Published:** 2023-11-10

**Authors:** Kangming Li, Daniel Persaud, Kamal Choudhary, Brian DeCost, Michael Greenwood, Jason Hattrick-Simpers

**Affiliations:** 1 Department of Materials Science and Engineering, University of Toronto, 27 King’s College Cir, Toronto, ON Canada; 2 Material Measurement Laboratory, National Institute of Standards and Technology, 100 Bureau Dr, Gaithersburg, MD USA; 3 Canmet MATERIALS, Natural Resources Canada, 183 Longwood Road south, Hamilton, ON Canada; 4 Acceleration Consortium, University of Toronto, 27 King’s College Cir, Toronto, ON Canada; 5 Vector Institute for Artificial Intelligence, 661 University Ave, Toronto, ON Canada; 6 Schwartz Reisman Institute for Technology and Society, 101 College St, Toronto, ON Canada

**Keywords:** Condensed-matter physics, Scaling laws, Materials chemistry, Scientific data

## Abstract

Extensive efforts to gather materials data have largely overlooked potential data redundancy. In this study, we present evidence of a significant degree of redundancy across multiple large datasets for various material properties, by revealing that up to 95% of data can be safely removed from machine learning training with little impact on in-distribution prediction performance. The redundant data is related to over-represented material types and does not mitigate the severe performance degradation on out-of-distribution samples. In addition, we show that uncertainty-based active learning algorithms can construct much smaller but equally informative datasets. We discuss the effectiveness of informative data in improving prediction performance and robustness and provide insights into efficient data acquisition and machine learning training. This work challenges the “bigger is better” mentality and calls for attention to the information richness of materials data rather than a narrow emphasis on data volume.

## Abstract (web summary)

Big data is crucial for machine learning, but the redundancies in the datasets are rarely studied. Here the authors reveal significant redundancy in large materials datasets, showing that up to 95% of data can be removed without impacting prediction accuracy.

## Introduction

Data is essential to the development and application of machine learning (ML), which has now become a widely adopted tool in materials science^1–11^. While data is generally considered to be scarce in various subfields of materials science, there are indications that the era of big data is emerging for certain crucial material properties. For instance, a substantial amount of material data has been produced through high-throughput density functional theory (DFT) calculations^12^, leading to the curation of several large databases with energy and band gap data for millions of crystal structures^13–17^. The recently released Open Catalyst datasets contain over 260 million DFT data points for catalyst modeling^18,19^. The quantity of available materials data is expected to grow at an accelerated rate, driven by the community’s growing interest in data collection and sharing.

In contrast to the extensive effort to gather ever larger volume of data, information richness of data has so far attracted little attention. Such a discussion is important as it can provide critical feedback to data acquisition strategies adopted in the community. For instance, DFT databases were typically constructed either from exhaustive enumerations over possible chemical combinations and known structural prototypes or from random sub-sampling of such enumerations^14–20^, but the effectiveness of these strategies in exploring the materials space remains unclear. Furthermore, existing datasets are often used as the starting point for the data acquisition in the next stage. For example, slab structures in Open Catalyst datasets were created based on the bulk materials from Materials Project^18,19^. Redundancy in the existing datasets, left unrecognized, may thus be passed on to future datasets, making subsequent data acquisition less efficient.

In addition, examining and eliminating redundancy in existing datasets can improve training efficiency of ML models. Indeed, the large volume of data already presents significant challenges in developing ML models due to the increasingly strong demand for compute power and long training time. For example, over 16,000 GPU days were recently used for analyzing and developing models on the Open Catalyst datasets^21^. Such training budgets are not available to most researchers, hence often limiting model development to smaller datasets or a portion of the available data^22^. On the other hand, recent work on image classification has shown that a small subset of data can be sufficient to train a model with performance comparable to that obtained using the entire dataset^23,24^. It has been reported that aggressively filtering training data can even lead to modest performance improvements on natural language tasks, in contrast to the prevailing wisdom of “bigger is better” in this field^25^. To the best of our knowledge, however, there has been no investigation of the presence and degree of data redundancy in materials science. Revealing data redundancy can inform and motivate the community to create smaller benchmark datasets, hence significantly scaling down the training costs and facilitating model development and selection. This may be important in the future if data volume grows much faster than the available training budget, which is a likely scenario, as data volume is proportional to resources available to the entire community, while training budgets are confined to individual research groups.

The examination of data redundancy is also important in other scenarios in materials science. Methods developed for selecting the most informative data can be used as the strong baselines for active learning algorithms, which are increasingly common in ML-driven materials discovery workflows^26–34^. Analysis of information richness can also improve our understanding of the material representation and guide the design of active learning algorithms. In the multi-fidelity data acquisition setting^35^, one can perform high-fidelity measurement only on the informative materials down-selected from the larger but low-fidelity datasets.

In this work, we present a systematic investigation of data redundancy across multiple large material datasets by examining the performance degradation as a function of training set size for traditional descriptor-based models and state-of-the-art neural networks. To identify informative training data, we propose a pruning algorithm and demonstrate that smaller training sets can be used without substantially compromising the ML model performance, highlighting the issue of data redundancy. We also find that selected sets of informative materials transfer well between different ML architectures, but may transfer poorly between substantially different material properties. Finally, we compare uncertainty-based active learning strategies with our pruning algorithm, and discuss the effectiveness of active learning for more efficient high throughput materials discovery and design.

## Results

### Redundancy evaluation tasks

We investigate data redundancy by examining the performance of ML models. To do so, we use the standard hold-out method for evaluating ML model performance: We create the training set and the hold-out test set from a random split of the given dataset. The training set is used for model training, while the test set is reserved for evaluating the model performance. In the following, we refer to the performance evaluated on this test set as the in-distribution (ID) performance, and this training set as the pool. To reveal data redundancy, we train a ML model on a portion of the pool and check whether its ID performance is comparable to the one resulting from using the entire pool. Since ID performance alone may not be sufficient to prove the redundancy of the remaining unused pool data, we further evaluate the prediction performance on the unused pool data and out-of-distribution (OOD) test data.

**Fig. 1:**
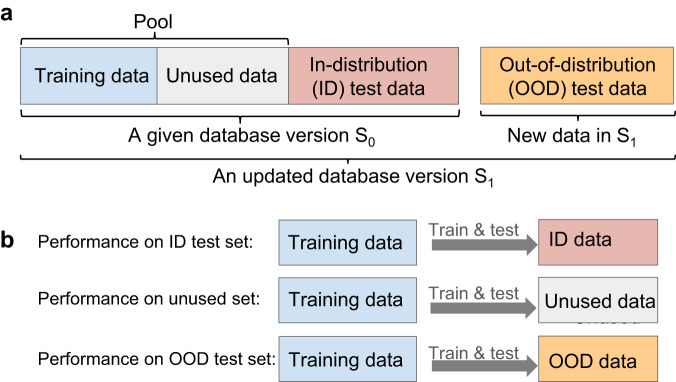
Schematic of redundancy evaluation. **a** The dataset splits. **b** Three Prediction tasks to evaluate model performance and data redundancy.

Figure 1 illustrates the redundancy evaluation discussed above. We first perform a (90, 10)% random split of the given dataset *S*_0_ to create the pool and the ID test set. To create an OOD test set, we consider new materials included in a more recent version of the database *S*_1_. Such OOD sets enable the examination of model performance robustness against distribution shifts that may occur when mission-driven research programs focus on new areas of material space^36^. We progressively reduce the training set size from 100% to 5% of the pool via a pruning algorithm (see “Methods”). ML models are trained for each training set size, and their performance is tested on the hold-out ID test data, the unused pool data, and the OOD data, respectively.

To ensure a comprehensive and robust assessment of data redundancy, we examine the formation energy, band gap, and bulk modulus data in three widely-used DFT databases, namely JARVIS^15^, Materials Project (MP)^16^, and OQMD^17^. For each database, we consider two release versions to study the OOD performance and to compare the data redundancy between different database versions. The number of entries for these datasets is given in Table 1.

**Table 1:** Number of entries of formation energy (*E*_*f*_), band gap (*E*_*g*_), and bulk modulus (*K*) data in JARVIS18, JARVIS22, MP18, MP21, OQMD14, and OQMD21 datasets

	JARVIS18	JARVIS22	MP18	MP21	OQMD14	OQMD21
*E*_*f*_	53k	76k	68k	146k	290k	1M
*E*_*g*_	53k	76k	68k	146k	290k	1M
*K*	19k	24k	7k	7k	0	0

The last two digits in the dataset name indicate the year of release (e.g., MP18 for the 2018 version).

To ascertain whether data redundancy is model-agnostic, we consider two conventional ML models, namely XGBoost (XGB)^37^ and random forests (RF)^38^, and a graph neural network called the Atomistic LIne Graph Neural Network (ALIGNN)^39^. The RF and XGB models are chosen since they are among the most powerful descriptor-based algorithms^40^, whereas ALIGNN is chosen as the representative neural network because of its state-of-the-art performance in the Matbench test suite^41^ at the time of writing.

### In-distribution performance

We begin by presenting an overview of the ID performance for all the model-property-dataset combinations in Table 2, where the root mean square errors (RMSE) of the models trained on the entire pool are compared to those obtained with 20% of the pool. For brevity, we refer to the models trained on the entire pool and on the subsets of the pool as the full and reduced models, respectively, but we note that the model specification is the same for both full and reduced models and the terms “reduced” and “full” pertain only to the amount of training data.

**Table 2:** RMSE scores obtained with the full and reduced models on the ID test set in JARVIS18, JARVIS22, MP18, MP21, OQMD14, and OQMD21 datasets

Dataset	STD	RF		XGB		ALIGNN	
		Full	20%	Full	20%	Full	20%
	Formation energy (eV atom^−1^)						
JARVIS18	1.08	0.187	0.190	0.136	0.159	0.064	0.093
JARVIS22	1.08	0.191	0.196	0.149	0.165	0.074	0.102
MP18	1.06	0.159	0.168	0.120	0.140	0.065	0.085
MP21	1.21	0.190	0.196	0.161	0.175	0.081	0.093
OQMD14	0.85	0.117	0.124	0.096	0.105	0.058	0.068
OQMD21	1.00	0.117	0.123	0.109	0.104	/	/
	Band gap (eV)						
JARVIS18	1.41	0.433	0.506	0.404	0.439	0.395	0.497
JARVIS22	1.33	0.406	0.465	0.385	0.411	0.365	0.441
MP18	1.62	0.613	0.738	0.587	0.658	0.613	0.743
MP21	1.51	0.555	0.683	0.535	0.616	0.529	0.682
OQMD14	0.72	0.211	0.212	0.196	0.198	0.185	0.189
OQMD21	0.87	0.308	0.314	0.314	0.323	/	/
	Bulk modulus (GPa)						
JARVIS18	66.6	24.6	26.8	23.7	27.1	22.9	29.6
MP18	75.8	22.0	23.0	18.7	24.2	16.0	31.2

The standard deviation (STD) of labels is also given in the second column. The reduced models are trained on the subset (20% of the pool) selected via the pruning algorithm. The ALIGNN results for the formation energy and band gap data in OQMD21 are not available because of the high training cost associated with the large data volume. Source data are provided as a Source data file.

*RF* random forest, *XGB* XGBoost, *ALIGNN* atomistic line graph neural network.

For the formation energy prediction, the RMSE of the reduced RF models increase by less than 6% compared to those of the full RF models in all cases. Similarly, the RMSE of the reduced XGB models increase only by 10 to 15% compared to the RMSE of the full XGB models in most datasets, except in OQMD21 where a 3% decrease in the RMSE is observed. The RMSE of the reduced ALIGNN models increase by 15 to 45%, a larger increment than observed for the RF and XGB models. Similar trend is observed for the band gap and bulk modulus prediction, where the RMSE of the reduced models typically increase by no more than 30% compared to those of the full models.

**Fig. 2:**
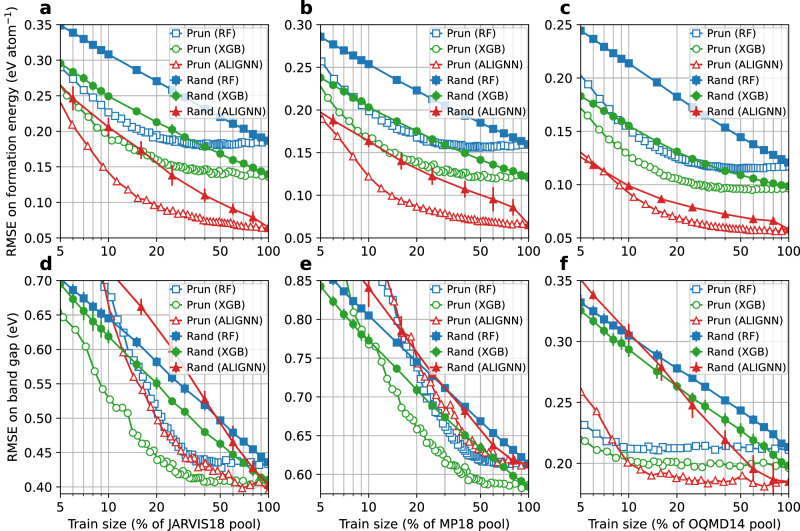
Root mean square error (RMSE) on the ID test sets. **a**–**c** JARVIS18, MP18, and OQMD14 formation energy prediction. **d**–**f** JARVIS18, MP18, and OQMD14 band gap prediction. RF random forest, XGB XGBoost, ALIGNN atomistic line graph neural network. The random baseline results to for the XGB and RF (or ALIGNN) models are obtained by averaging over the results of 10 (or 5) random data selections for each training set size, with the error bars denoting the standard deviations. The *X* axis is in the log scale. Data points are connected by straight lines. Source data are provided as a Source data file.

Next, we conduct a detailed analysis for formation energy and band gap properties because of their fundamental importance for a wide range of materials design problems. Figure 2 shows the ID performance as a function of training set size (in percentage of the pool) for the formation energy and band gap prediction in the JARVIS18, MP18, and OQMD14 datasets. Results for other datasets can be found in Supplementary Figs. 1–6.

For the formation energy prediction, the prediction error obtained with the pruned data drops much faster with increasing data size than the one obtained using the randomly selected data. When accounting for more than 5% of the training pool, the pruned datasets lead to better ID performance than the ones from random sampling. In particular, the RF, XGB, and ALIGNN models trained with 20% of the pool selected by the pruning algorithm have the same ID performance as the ones trained with a random selection of around 90%, 70%, and 50%, respectively, of the pool.

A large portion of training data can be removed without significantly hurting the model performance. To demonstrate this, we define a quantitative threshold for the “significance” of the performance degradation as a 10% relative increase in RMSE; data that can be pruned without exceeding this performance degradation threshold are considered redundant. With this definition, only 13% of the JARVIS18 data, and 17% of the MP18 and OQMD data are informative for the RF models. For the XGB models, between 20% and 30% of the data are needed depending on the datasets. For the ALIGNN models, 55%, 40%, and 30% of the JARVIS18, MP18, and OQMD14 data are informative, respectively. While the JARVIS18 dataset may seem to be less redundant for the ALIGNN models, the 10% increase in the RMSE (60 meV atom^−1^) corresponds to an RMSE increase of only 6 meV atom^−1^, much smaller than the DFT accuracy of around 100 meV atom^−1^ with respect to experiments^42^. In fact, training the ALIGNN model on 30% of the JARVIS18 data only leads to a drop of 0.002 in the *R*^2^ test score.

While this work is focused on redundancy which is model and dataset specific, it is still worth commenting on the model performance scaling across models and datasets. When using the random sampling for data selection, we observe a power law scaling for all the models and datasets. For formation energy datasets, switching the models mainly shifts the scaling curve without much change to the slopes. For band gap datasets, switching from RF to XGB models shifts the scaling curve down without changing the slope, whereas switching from tree-based models to ALIGNN leads to a steeper slope and hence better scaling. Compared to training on randomly sampled data, training on informative data as selected by the pruning algorithm can lead to better scaling until reaching saturation when there is no more informative data in the pool. Different datasets exhibit similar scaling behaviors with the slope and saturation point dependent on target property and material space covered by the datasets.

The performance response to the size of band gap data is similar to that observed in the formation energy data. The redundancy issue is also evident in band gap data: a 10% RMSE increase corresponds to training with 25 to 40% of the data in the JARVIS18 and MP18 datasets. Even more strikingly, only 5% (or 10%) of the OQMD14 band gap data are sufficiently informative for the RF and XGB (or ALIGNN) models.

**Fig. 3:**
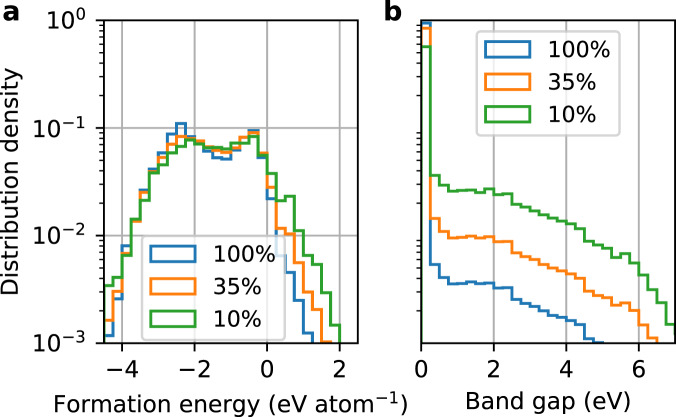
Label distributions of the training sets pruned by the XGBoost (XGB) model. **a** Formation energy data from the MP18 dataset. **b** Band gap data from the OQMD14 dataset. The legend indicates the training set size in percentage of the pool. Results for other datasets can be found in Supplementary Figs. 15 and 16. Source data are provided as a Source data file.

These results demonstrate the feasibility of training on only a small portion of the available data without much performance degradation. We find that this is achieved by skewing the data distribution towards the underrepresented materials. For instance, the distributions of the pruned data are skewed towards materials with large formation energies and band gaps (Fig. 3), which are both underrepresented and less accurately predicted materials. These results not only confirm the importance of the data diversity^40^ but also highlight the redundancy associated with overrepresented materials.

ID performance is not sufficient to prove that the unused data are truly redundant. The effects related to model capability and the test set distribution should also be considered. Indeed, one may argue that the current ML models (in particular, the band gap models) are not advanced enough to learn from the unused data leading to a false sense of the data redundancy. Furthermore, the similar performance of the full and reduced models does not imply a similar performance on a test set following a different distribution. These questions are addressed in the following two sections by discussing the performance on the unused data and on the OOD data.

### Performance on unused data

**Fig. 4:**
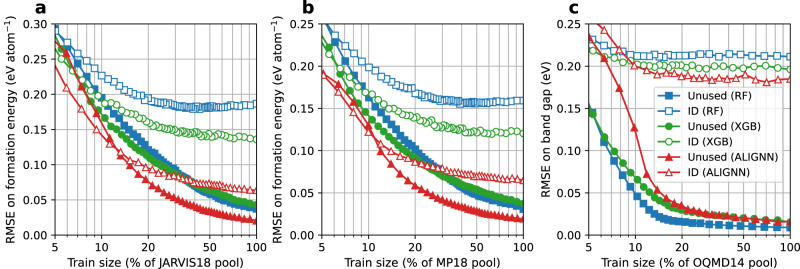
Root mean square error (RMSE) on the unused data in the pool. **a** JARVIS18 formation energy prediction. **b** MP18 formation energy prediction. **c** OQMD14 band gap prediction. RF random forest, XGB XGBoost, ALIGNN atomistic line graph neural network. Performance on the ID test set is shown for comparison. Data points are connected by straight lines. Source data are provided as a Source data file.

Here we further examine the model performance on the unused pool data. Figure 4 shows three representative cases: the JARVIS18 and MP18 formation energy datasets, and the OQMD14 band gap dataset. For the formation energy prediction, the RMSE on the unused data become lower than on the ID RMSE when the training set size is above 5 to 12% of the pool, and is half of the ID RMSE when the training set size is above 30 to 40% of the pool. Similar trend is observed for the band gap prediction with varying thresholds of the performance improvement saturation depending the datasets (Supplementary Figs. 10–12). In particular, the OQMD14 results in Fig. 4 show that the models trained on 10% of the pool can well predict the unused data that account for 90% of the pool, with the associated RMSE much lower than the RMSE on the ID test set. The good prediction on the unused data signifies a lack of new information in these data, confirming that the improvement saturation in the ID performance is caused by the information redundancy in the unused data rather than the incapability of models to learn new information.

While the scaling curve for the unused data has a shape similar to the one for the ID test data, the former shows a much steeper slope for the training set sizes below 15% of the pool, and reaches saturation at a slower rate. In addition, it is noted that the ranking of different ML models for their performance on the unused data is not necessarily the same as for the ID test data. For instance, for the JARVIS18 and MP18 formation energy data, the XGB model outperforms the RF model on the ID test set whereas their performance is practically the same on the unused data. Among the models trained on the OQMD14 band gap data, the RF model has the largest RMSE on the ID test set but the lowest error on the unused data.

### Out-of-distribution performance

To check whether redundancy in training data also manifests under a distribution shift in test data, we examine the model performance on the OOD test data consisting of the new materials in the latest database versions (JARVIS22, MP21, and OQMD21) using the models trained on the older versions (JARVIS18, MP18, and OQMD14).

**Fig. 5:**
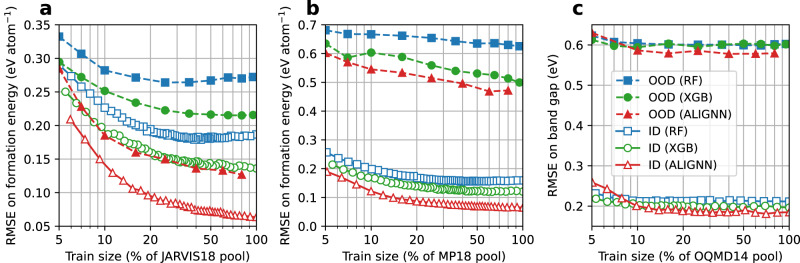
Root mean square error (RMSE) on the OOD test sets. **a** JARVIS formation energy prediction. **b** MP formation energy prediction. **c** OQMD band gap prediction. RF random forest, XGB XGBoost, ALIGNN atomistic line graph neural network. Performance on the ID test set is shown for comparison. Data points are connected by straight lines. The reader interested in the statistical overlaps between the ID and OOD data in the feature space is referred to Supplementary Fig. 24. Source data are provided as a Source data file.

First, we find that training on the pruned data can lead to better or similar OOD performance than the randomly sampled data of the same size. We therefore focus here on the OOD performance based on the pruned data shown in Fig. 5. Overall, the scaling curves for the OOD performance show are similar to those for the ID performance with slightly different slopes and saturation data size, confirming the existence of the data redundancy measured by the OOD performance. Specifically, using 20%, 30%, or 5% to 10% of the JARVIS18, MP18, or OQMD14 data, respectively, can lead to an OOD performance similar to that of the full models, with around 10% RMSE increase.

The performance on OOD data can be severely degraded. Even for the models trained on the entire pool, the increase in the OOD RMSE with respect to the ID RMSE often goes above 200% for the considered databases and can rise up to 640% in the case of the ALIGNN-MP formation energy prediction (Supplementary Table 1). Therefore, the excellent ID performance obtained with state-of-the-art models and large datasets might be a catastrophically optimistic estimation of the true generalization performance in a realistic materials discovery setting^36,40^.

Different databases exhibit a varying degree of performance degradation, which should be correlated with the degree of statistical overlaps between the database versions rather than the quality of the databases. In fact, database updates that induce such performance degradation are desirable because they are indications of new “unknown” observations and can lead to more robust generalization performance. One interesting line of research would be therefore to develop methods to deliberately search for materials where the previous models would fail catastrophically as a path to expand a database.

The strong OOD performance degradation highlights the importance of information richness over data volume. It also raises an interesting question: given a training set *A*_1_, is it possible to find a smaller training set *A*_2_ such that the *A*_2_-trained model perform similarly to the *A*_1_-trained model on an *A*_1_-favorable test set *B*_1_ (i.e., same distribution as *A*_1_) but significantly outperform the *A*_1_-trained model on an *A*_1_-unfavorable test set B_*_ (i.e., distribution different from *A*_1_)? Indeed, we find that training on the heavily pruned MP21 pool (*A*_2_) gives dramatically better prediction performance on the MP21 test data (B_*_) than training on 10 × more data from the MP18 pool (*A*_1_) whereas their performance is similar on the MP18 test set (*B*_1_). The result confirms the idea of finding a training set whose distribution can not only well cover but also significantly extend beyond the original one while still being much smaller in size. The result highlights that information richness and data volume are not necessarily correlated, and the former is much more important for the prediction robustness. By covering more materials within the data distribution, we may better ensure unknown materials are from known distributions (“known unknown”) and avoid unexpected performance degradation (“unknown unknown”), which is particularly important in scenarios such as materials discovery or building universal interatomic potentials^22,43,44^.

### Transferability of pruned material sets

The ID performance results demonstrate that our pruning algorithm effectively identifies informative material sets for a given ML model and material property. A natural follow-up inquiry is the universality, or more specifically, the transferability of these sets between ML architectures and material properties.

We find a reasonable level of transferability of the pruned material set across ML architectures, confirming that data pruned by a given ML architecture remains informative to other ones (Supplementary Figs. 17–20). For example, XGB models trained on RF-pruned data outperform those trained on twice as much randomly selected data for formation energy prediction. Moreover, the XGB model still outperforms an RF model trained on the same pruned data, consistent with our observed performance ranking (XGB > RF). This ensures robustness against information loss with respect to future architecture change: more capable models developed in the future can be expected to extract no less information from the pruned dataset than the current state-of-the-art one, even if the dataset is pruned by the latter. It would therefore be desirable to propose benchmark datasets pruned from existing large databases using current models, which can help accelerate the development of ML models due to the smaller training cost.

In contrast, we find that there is a limited transferability of pruned datasets across different material properties. For instance, the band gap models trained on the pruned formation energy data outperform those trained on randomly sampled data by only by a slight margin (Supplementary Fig. 21), suggesting little overlap between informative material sets for predicting these two properties. This limited task transferability may be a result of the lack of strong correlation between the formation energy and band gap data, for which the Spearman correlation coefficient is −0.5 in the considered databases. Additionally, the OOD results show that formation energy and band gap models do not necessarily suffer the same degree of performance degradation when tested on new materials despite being trained on the same set of materials (Supplementary Table 1), indicating learned feature-property relations could differ significantly. These considerations suggest that a fruitful line of future research might explore dataset pruning based on multitask regression models focusing on a diverse set of material properties controlled by different underlying physical phenomena.

### Uncertainty-based active learning

In the previous sections we have revealed the data redundancy in the existing large material databases through dataset pruning. How much, then, can we avoid such data redundancy in the first place when constructing the databases? To this end, we consider active learning algorithms that select samples with largest prediction uncertainty (see “Methods”). The first and the second algorithms use the width of the 90% prediction intervals of the RF and XGB models as the uncertainty measure, respectively, whereas the third one is based on the query by committee (QBC), where the uncertainty is taken as the disagreement between the RF and XGB predictions.

**Fig. 6:**
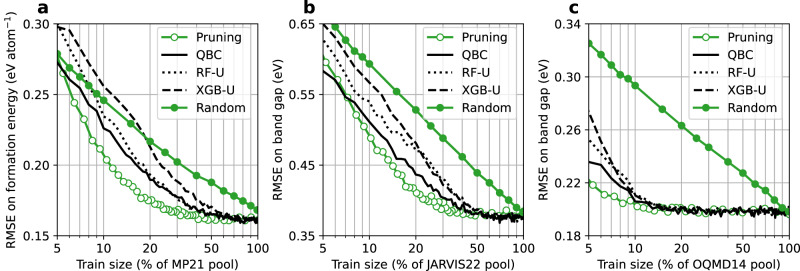
RMSE on the ID test sets by the XGB models trained on the data selected using the active learning algorithms. **a** MP21 formation energy prediction. **b** JARVIS22 formation energy prediction. **c** OQMD14 band gap prediction. QBC query by committee, RF-U random forest uncertainty, XGB-U XGBoost uncertainty. The performance obtained using the random sampling and the pruning algorithm is shown for comparison. Data points are connected by straight lines. Source data are provided as a Source data file.

Figure 6 shows a comparison of the ID performance of the XGB models trained on the data selected using the active learning algorithm, the pruning algorithm, and the random sampling. The QBC algorithm is found to be the best performing active learning algorithm. For the formation energy prediction across the three databases, 30 to 35% of the pool data selected by the QBC algorithm is enough to achieve the same model performance obtained with 20% of the pool data using the pruning algorithm. Furthermore, the resulting model performance is equivalent to that obtained with 70 to 90% of the pool using the random sampling. As for the band gap prediction, the models trained on the QBC-selected data perform similarly to those trained on the pruned data, or even sometimes outperform the latter when the data volume is below 20% (Supplementary Fig. 23). In particular, the QBC algorithm can effectively identify 10% of the OQMD14 band gap data as the training data without hurting the model performance (Fig. 6c). Similar trends are also found for the RF models and for other datasets (Supplementary Fig. 23).

Overall, our results across multiple datasets suggest that it is possible to leverage active learning algorithms to query only 30% of the existing data with a relatively small accuracy loss in the ID prediction. The remaining 70% of the compute may then be used to obtain a larger and more representative material space. Considering the potentially severe performance degradation on OOD samples which are likely to be encountered in material discovery, the gain in the robustness of ML models may be preferred over the incremental gain in the ID performance.

## Discussion

It is worth emphasizing that this work is by no means critical of the curation efforts or significance of these materials datasets. Indeed, many datasets were not originally generated for training ML models; they are the results of long-term project-driven computational campaigns. Some of them were even curated before the widespread use of ML and have played a significant role in fueling the rapid adoption of ML in materials science. On the other hand, the presence and degree of redundancy in a dataset is worth discussing irrespective of the original purpose. Furthermore, ML should be considered not only as a purpose, though it has become a primary use case of these datasets, but also as a statistical means of examining and improving these datasets.

This work is also not to oppose the use of big data, but to advocate a critical assessment of the information richness in data, which has been largely overlooked due to a narrow emphasis on data volume. As materials science transitions towards a big data-driven approach, such evaluations and reflections on current practices and data can offer insights into more efficient data acquisition and sensible resource usage. For instance, conventional high-throughput DFT often relies on enumerations over structural prototypes and chemical combinations. The substantial redundancy revealed in this work suggests these strategies are suboptimal in querying new informative data, whereas uncertainty based active learning can enable a 3× to 10× boost in sampling efficiency. Our scaling results for OOD performance degradation further highlight the importance of information richness over sheer volume for robust predictive models. In this regard, it is preferable to allocate more resources to explore a diverse materials space rather than seeking incremental improvements in prediction accuracy within limited or well-studied regions. This may represent a paradigm shift from systematic high-throughput studies, where we can start with uncertainty based active learning in a much larger design space, and then reconsider the design space by interrogating the model and switching to a property optimization or local interpretable prediction objective.

While the pruning algorithm is proposed here to illustrate data redundancy, such data selection algorithms can have other use cases, e.g., inform the design of active learning algorithms. Indeed, the observation that data redundancy predominantly involves overrepresented materials implies that information entropy might also serve as a promising criterion for data acquisition^40,45^. A detailed analysis of pruned material sets may also offer insights into material prototypes and improve understanding of feature-property relationships, including identifying specific groups of redundant materials as well as identifying patterns that explain the poor task transferability of pruned datasets. Finally, the pruning algorithm offers a new funneling strategy for prioritizing materials for high-fidelity measurements. For instance, pruning the existing DFT data obtained with generalized gradient approximation (GGA) functionals can point to the materials to be recomputed with high-fidelity meta-GGA functionals^35^.

We demonstrate that transferability of compact datasets is reasonable across models but is limited across tasks (materials properties). It is discussed in the context of data pruning, but the idea and implication hold for active learning. The limited task transferability indicates that the maximally compact set of materials for property A is not ensured to be the maximally compact set for property B. While this is an interesting observation and invites further investigation, it is not a practical issue for active learning when the measurements of two properties are independent. For example, DFT calculations of band gap and elastic modulus are unrelated, therefore the maximally compact sets of materials can be constructed independently via active learning and need not be the same. For correlated property measurements, however, more careful planning is required. For instance, the calculations of more “expensive” properties such as band gap and elastic modulus would also give the formation energy of the same material since energy is a basic output of any DFT calculations. While the compact datasets for band gap and elastic modulus can still be searched independently without considering formation energy data, the construction of the compact dataset for formation energy should consider the data that can be obtained as by-products from the band gap and elastic modulus calculations.

In conclusion, we investigate data redundancy across multiple material datasets using both conventional ML models and state-of-the-art neural networks. We propose a pruning algorithm to remove uninformative data from the training set, resulting in models that outperform those trained on randomly selected data of the same size. Depending on the dataset and ML architecture, up to 95% of data can be pruned with little degradation in in-distribution performance (defined as <10% increase in RMSE) compared to training on all available data. The removed data, mainly associated with overrepresented material types, are shown to be well predicted by the reduced models trained without them, confirming again the information redundancy. Using new materials in newer database versions as the out-of-distribution test set, we find that 70 to 95% of data can be removed from the training set without exceeding a 10% performance degradation threshold on out-of-distribution data, confirming again that the removed data are redundant and do not lead to improved performance robustness against distribution shift. Transferability analysis shows that the information content of pruned datasets transfers well to different ML architectures but less so between material properties. Finally, we show that the QBC active learning algorithm can achieve an efficiency comparable to the pruning algorithm in terms of finding informative data, hence demonstrating the feasibility of constructing much smaller material databases while still maintaining a high level of information richness. While active learning algorithm may still induce bias in the datasets they generate, we believe that there is an exciting opportunity to optimize high throughput material simulation studies for generalization on a broad array of material property prediction tasks.

## Methods

### Materials datasets

The 2018.06.01 version of Materials Project (MP18), and the 2018.07.07 and 2022.12.12 versions of JARVIS (JARVIS18 and JARVIS22) were retrieved by using JARVIS-tools^15^. The 2021.11.10 version of Materials Project (MP21) was retrieved using the Materials Project API^16^. The OQMD14 and OQMD21 data were retrieved from https://oqmd.org/download.

The JARVIS22, MP21, OQMD21 data were preprocessed as follows. First, entries of materials with a formation energy larger than 5 eV atom^−1^ were removed. Then, the Voronoi tessellation scheme^46^ as implemented in Matminer^47^ were used to extract 273 compositional and structural features. The Voronoi tessellation did not work for a very small number of materials and these materials were removed.

For the older versions (JARVIS18, MP18, OQMD14), we did not directly use the structures and label values from the older database. Instead, we use the materials identifiers from the older database to search for the corresponding structures and label values in the newer database. This is to avoid any potential inconsistency caused by the database update.

### ML models

We considered three ML models here: XGB^37^, RF^38^, and a graph neural network called the Atomistic LIne Graph Neural Network (ALIGNN)^39^. XGB is a gradient-boosted method that builds sequentially a number of decision trees in a way such that each subsequent tree tries to reduce the residuals of the previous one. RF is an ensemble learning method that combines multiple independently built decision trees to improve accuracy and minimize variance. ALIGNN constructs and utilizes graphs of interatomic bonds and bond angles.

We used the RF model as implemented in the scikit-learn 1.2.0 package^48^, and the XGB model as implemented in the XGBoost 1.7.1 package^37^. For the RF model, we used 100 estimators, 30% of the features for the best splitting, and default settings for other hyperparameters. We used a boosted random forest for the XGB model: 4 parallel boosted trees were used; for each tree, we used 1000 estimators, a learning rate of 0.1, an L1 (L2) regularization strength of 0.01 (0.1), and the histogram tree grow method; we set the subsample ratio of training instances to 0.85, the subsample ratio of columns to 0.3 when constructing each tree, and the subsample ratio of columns to 0.5 for each level. The hyperparameter set was kept to be the same in all the model training for the following reasons: First, performing hyperparameter tuning every time when changing the size of the training set would be very computationally expensive. Second, we verified that the model performance using the optimal hyperparameters tuned from the randomized cross-validation search was close to the one using the chosen hyperparameters.

For the ALIGNN model, we used 2 ALIGNN layers, 2 GCN layers, a batch size of 128, and the layer normalization, while keeping other hyperparameters the same as in the original ALIGNN implementation^39^. We trained the ALIGNN model for 50 epochs as we found more epochs did not lead to further performance improvement. We used the same OneCycle learning rate schedule, with 30% of the training budget allocated to linear warmup and 70% to cosine annealing.

### Pruning algorithm

We proposed a pruning algorithm that starts with the full training pool and iteratively reduces the training set size. We denote the full training pool as *D*_pool_, the training set at the *i*-th iteration as ${D}_{{{{{{{{\rm{train}}}}}}}}}^{i}$, the unused set as ${D}_{{{{{{{{\rm{unused}}}}}}}}}^{i}$ ($={D}_{{{{{{{{\rm{pool}}}}}}}}}-{D}_{{{{{{{{\rm{train}}}}}}}}}^{i}$), and the trained model as *M*^*i*^. At the initial iteration (*i* = 0), ${D}_{{{{{{{{\rm{train}}}}}}}}}^{0}={D}_{{{{{{{{\rm{pool}}}}}}}}}$, and ${D}_{{{{{{{{\rm{unused}}}}}}}}}^{0}$ is empty. At each iteration *i* > 0, ${D}_{{{{{{{{\rm{train}}}}}}}}}^{i}$ and ${D}_{{{{{{{{\rm{unused}}}}}}}}}^{i}$ are updated as follows: First, a random splitting of ${D}_{{{{{{{{\rm{train}}}}}}}}}^{i-1}$ is performed to obtained two subsets ${D}_{A}^{i}$ (80% of ${D}_{{{{{{{{\rm{train}}}}}}}}}^{i-1}$) and ${D}_{B}^{i}$ (20% of ${D}_{{{{{{{{\rm{train}}}}}}}}}^{i-1}$). Then, a model $M{\prime}$ is trained on ${D}_{A}^{i}$ and then tested on ${D}_{B}^{i}$. The data in ${D}_{B}^{i}$ with lowest prediction errors (denoted as ${D}_{B,{{{{{{\rm{unused}}}}}}}}^{i}$) are then removed from the training set. Namely, ${D}_{{{{{{{{\rm{train}}}}}}}}}^{i}={D}_{{{{{{{{\rm{train}}}}}}}}}^{i-1}-{D}_{B,{{{{{{\rm{unused}}}}}}}}^{i}$, and ${D}_{{{{{{{{\rm{unused}}}}}}}}}^{i}$ = ${D}_{{{{{{{{\rm{unused}}}}}}}}}^{i-1}+{D}_{B,{{{{{{\rm{unused}}}}}}}}^{i}$. The model *M*^*i*^ trained on ${D}_{{{{{{{{\rm{train}}}}}}}}}^{i}$ is then used in the performance evaluation on the ID test set, the unused set ${D}_{{{{{{{{\rm{unused}}}}}}}}}^{i}$ and the OOD test set.

### Active learning algorithm

During the active learning process, the training set is initially constructed by randomly sampling 1 to 2% of the pool, and is grown with a batch size of 1 to 2% of the pool by selecting the materials with maximal prediction uncertainty. Three uncertainty measures are used to rank the materials. The first one is based on the uncertainty of the RF model and is calculated as the difference between the 95th and 5th percentile of the tree predictions in the forest. The second one is based on the uncertainty of the XGB model using an instance-based uncertainty estimation for gradient-boosted regression trees developed in ref. ^49^. The third one is based on the query by committee, where the uncertainty is taken as the difference between the RF and XGB predictions.

### Reporting summary

Further information on research design is available in the Nature Portfolio Reporting Summary linked to this article.

### Supplementary information

Supplementary Information

Peer Review File

Reporting Summary

### Source data

Source data

## Data Availability

The data required and generated by our code are available on Zenodo at https://zenodo.org/record/8200972. Source data are provided with this paper.

## Code availability

The code used in this work is available on GitHub at https://github.com/mathsphy/paper-data-redundancy and a snapshot of the code is provided on Zenodo^50^.
