# Publisher Correction: Exploiting redundancy in large materials datasets for efficient machine learning with less data

**DOI:** 10.1038/s41467-023-44462-x

**Published:** 2024-01-04

**Authors:** Kangming Li, Daniel Persaud, Kamal Choudhary, Brian DeCost, Michael Greenwood, Jason Hattrick-Simpers

**Affiliations:** 1 Department of Materials Science and Engineering, University of Toronto, 27 King’s College Cir, Toronto, ON Canada; 2 Material Measurement Laboratory, National Institute of Standards and Technology, 100 Bureau Dr, Gaithersburg, MD USA; 3 Canmet MATERIALS, Natural Resources Canada, 183 Longwood Road south, Hamilton, ON Canada; 4 Acceleration Consortium, University of Toronto, 27 King’s College Cir, Toronto, ON Canada; 5 Vector Institute for Artificial Intelligence, 661 University Ave, Toronto, ON Canada; 6 Schwartz Reisman Institute for Technology and Society, 101 College St, Toronto, ON Canada

**Keywords:** Condensed-matter physics, Scaling laws, Materials chemistry, Scientific data

Correction to: *Nature Communications* 10.1038/s41467-023-42992-y, published online 10 November 2023

The copyright for this article was incorrectly given as ‘The Author(s)’ but should have been ‘His Majesty the King in Right of Canada as represented by the Minister of Natural Resources’. The original article has been corrected.

