# Supplementary material for: Exploiting redundancy in large materials datasets for efficient machine learning with less data
Source: Nat Commun. 2023 Nov 10;14:7283. doi: 10.1038/s41467-023-42992-y (PMC10638383; doi:10.1038/s41467-023-42992-y)
Supplement: Supplementary file 1 — Supplementary Information [file 41467_2023_42992_MOESM1_ESM.pdf]

Supplemental information: Exploiting redundancy in large materials datasets for efficient machine learning with less data

Kangming Li,[1] Daniel Persaud,[1] Kamal Choudhary,[2,3] Brian DeCost,[2] Michael Greenwood,[4] and Jason Hattrick-Simpers[1,5,6,7,*]

[1]*Department of Materials Science and Engineering, University of Toronto, 27 King's College Cir, Toronto, ON, Canada.*

[2]*Material Measurement Laboratory, National Institute of Standards and Technology, 100 Bureau Dr, Gaithersburg, MD, USA.*

[3]*Theiss Research, La Jolla, CA 92037, USA.*

[4]*Canmet MATERIALS, Natural Resources Canada, 183 Longwood Road south, Hamilton, ON, Canada.*

[5]*Acceleration Consortium, University of Toronto, 27 King's College Cir, Toronto, ON, Canada.*

[6]*Vector Institute for Artificial Intelligence, University of Toronto, 661 University Ave, Toronto, ON, Canada.*

[7]*Schwartz Reisman Institute for Technology and Society, University of Toronto, 101 College St, Toronto, ON, Canada.*

In this document, we provide the supplementary information for: the model performance on in-distribution (ID) test data, the unused data in the pool and the out-of-distribution (OOD) test data in Sec. I; the label distribution of the pruned data sets in Sec.II; the transferability of pruned material sets in Sec. III; the performance of the uncertainty-based active learning algorithms in Sec. IV; the statistical overlaps between the older databases and OOD data in Sec. V.

CONTENTS

I. Model performance on ID, unused, and OOD test data 2

A. Performance on ID test set 3

B. Performance on unused data 10

C. Performance on OOD test set 17

II. Label distribution of the pruned data sets 20

III. Transferability of pruned material sets 23

A. Transferability between ML models 23

B. Transferability between material properties 28

IV. Uncertainty-based active learning 29

V. Statistical overlap between the training pool and OOD data 33

References 34

I. MODEL PERFORMANCE ON ID, UNUSED, AND OOD TEST DATA

In this section, we present the supplementary figures of the model performance, namely the root mean square errors (RMSE), and the coefficients of determination (R^2), for the formation energy and band gap predictions of the JARVIS, MP, and OQMD datasets. The model performance on the in-distribution (ID) test set, the unused data in the pool, and the out-of-distribution (OOD) test set is presented in Sec. I A, Sec. I B, and Sec. I C, respectively.

* Correspondence: jason.hattrick.simpers@utoronto.ca

A. Performance on ID test set

The ID performance (root mean square error and R^2) of the formation energy models is shown in Fig. 1 for the JARVIS18 and JARVIS22 datasets, Fig. 2 for the MP18 and MP21 datasets, and Fig. 3 for the OQMD14 and OQMD21 datasets.

The ID performance (root mean square error and R^2) of the band gap models is shown in Fig. 4 for the JARVIS18 and JARVIS22 datasets, Fig. 5 for the MP18 and MP21 datasets, and Fig. 6 for the OQMD14 and OQMD21 datasets.

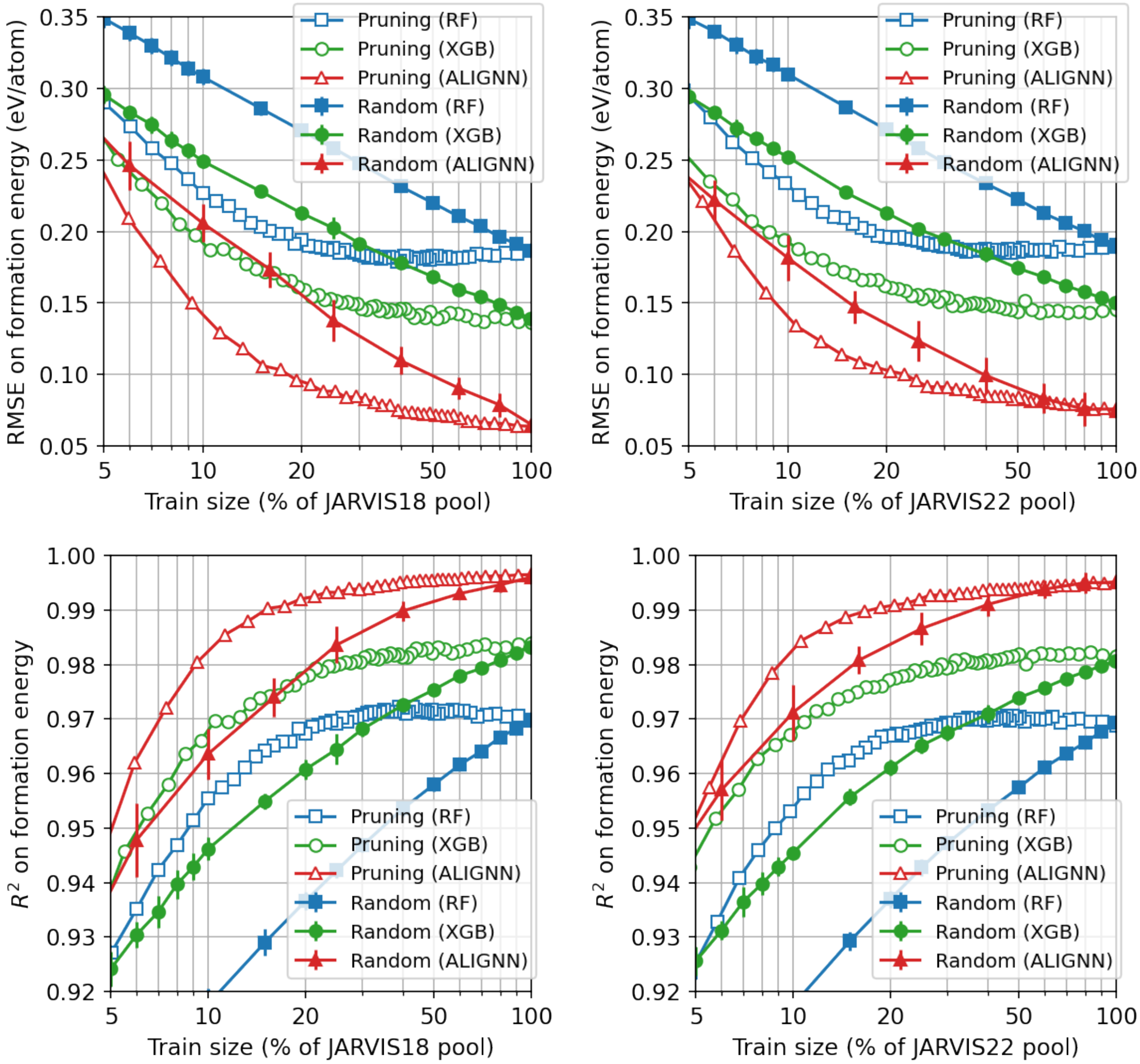

Supplementary Figure 1. Root mean square error (1st row) and R^2 (2nd row) on the ID test sets for the JARVIS18 (1st column) and JARVIS22 (2nd column) formation energy prediction. RF: random forest. XGB: XGBoost. ALIGNN: atomistic line graph neural network.

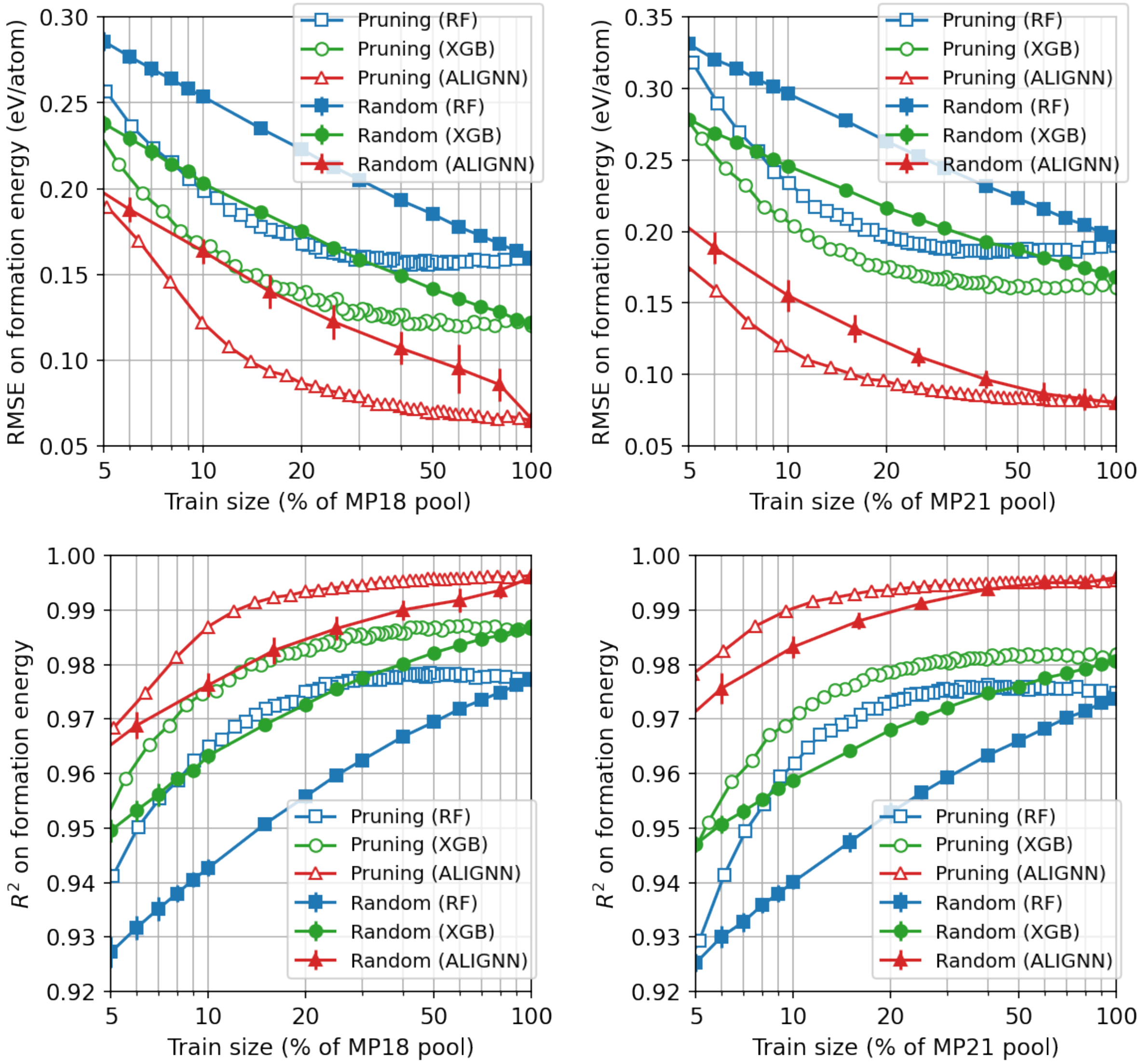

Supplementary Figure 2. Root mean square error (1st row) and R^2 (2nd row) on the ID test sets for the MP18 (1st column) and MP21 (2nd column) formation energy prediction. RF: random forest. XGB: XGBoost. ALIGNN: atomistic line graph neural network.

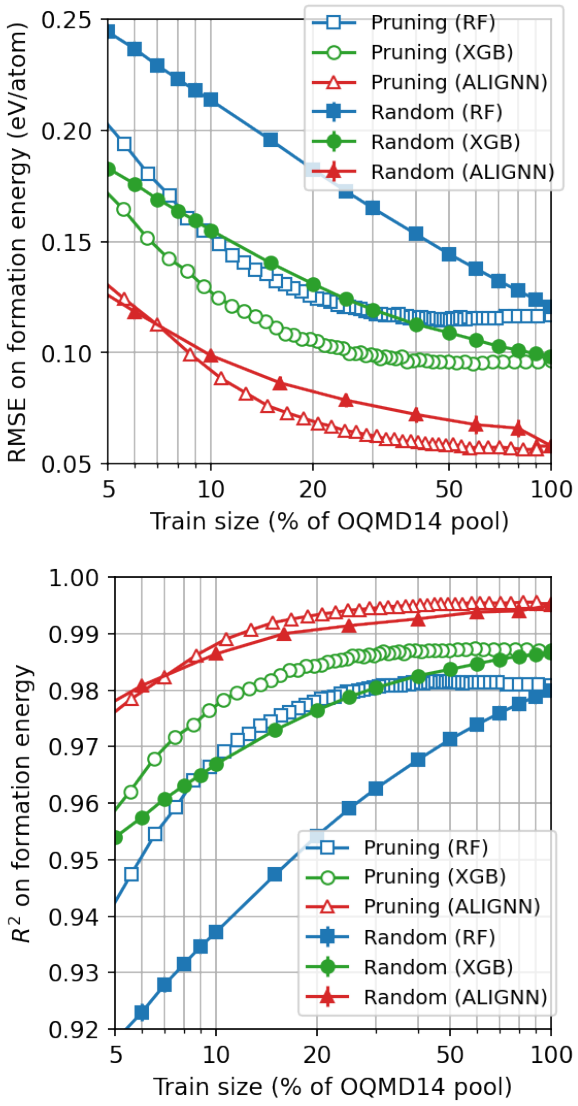

Supplementary Figure 3. Root mean square error (1st row) and R^2 (2nd row) on the ID test sets for the OQMD14 (1st column) and OQMD21 (2nd column) formation energy prediction. RF: random forest. XGB: XGBoost. ALIGNN: atomistic line graph neural network.

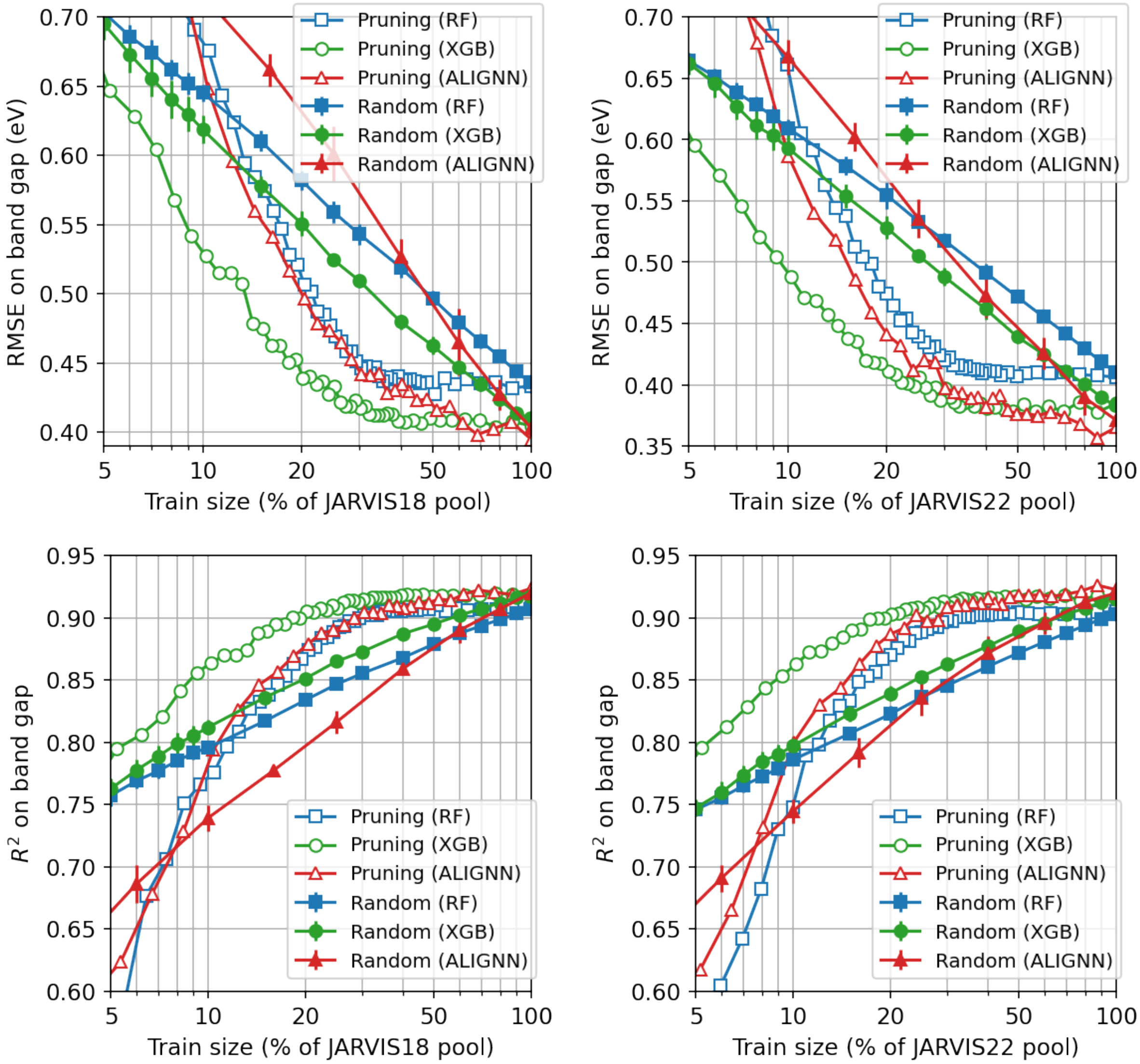

Supplementary Figure 4. Root mean square error (1st row) and R^2 (2nd row) on the ID test sets for the JARVIS18 (1st column) and JARVIS22 (2nd column) band gap prediction. RF: random forest. XGB: XGBoost. ALIGNN: atomistic line graph neural network.

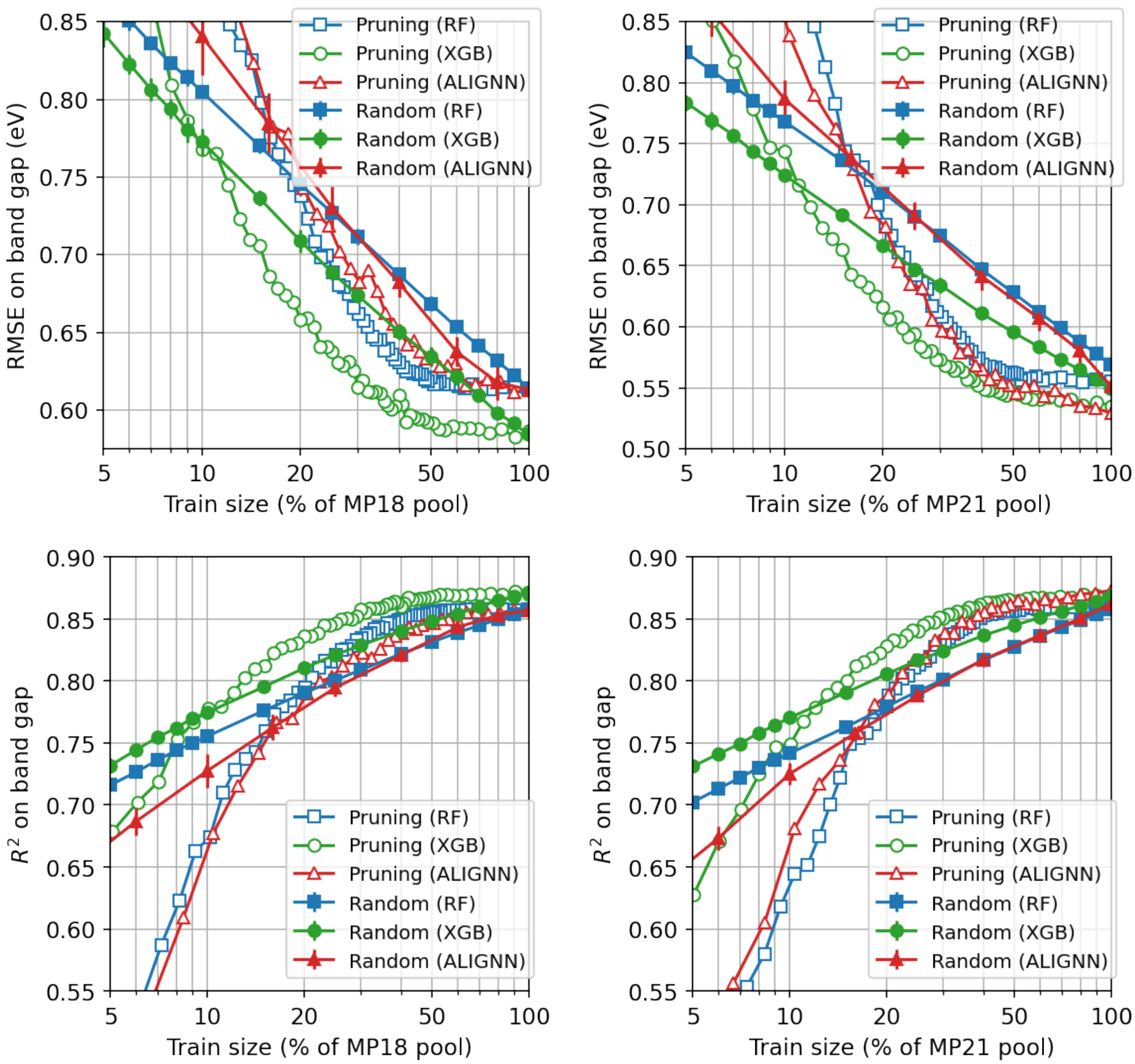

Supplementary Figure 5. Root mean square error (1st row) and R^2 (2nd row) on the ID test sets for the MP18 (1st column) and MP21 (2nd column) band gap prediction. RF: random forest. XGB: XGBoost. ALIGNN: atomistic line graph neural network.

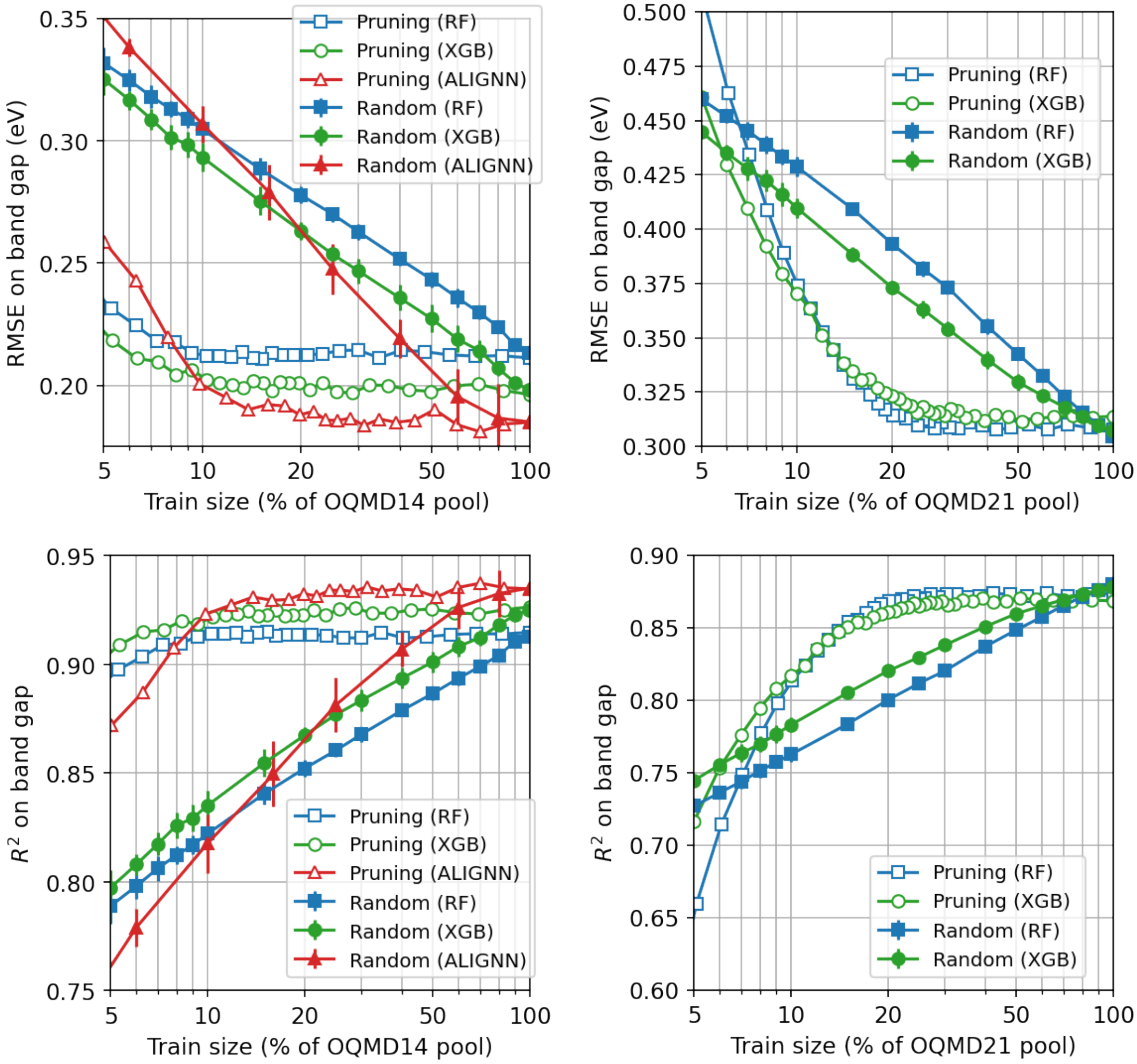

Supplementary Figure 6. Root mean square error (1st row) and R^2 (2nd row) on the ID test sets for the OQMD14 (1st column) and OQMD21 (2nd column) band gap prediction. RF: random forest. XGB: XGBoost. ALIGNN: atomistic line graph neural network.

B. Performance on unused data

The performance (root mean square error and R^2) of the formation energy models on the unused data is shown in Fig. 7 for the JARVIS18 and JARVIS22 datasets, Fig. 8 for the MP18 and MP21 datasets, and Fig. 9 for the OQMD14 and OQMD21 datasets.

The performance (root mean square error and R^2) of the band gap models on the unused data is shown in Fig. 10 for the JARVIS18 and JARVIS22 datasets, Fig. 11 for the MP18 and MP21 datasets, and Fig. 12 for the OQMD14 and OQMD21 datasets.

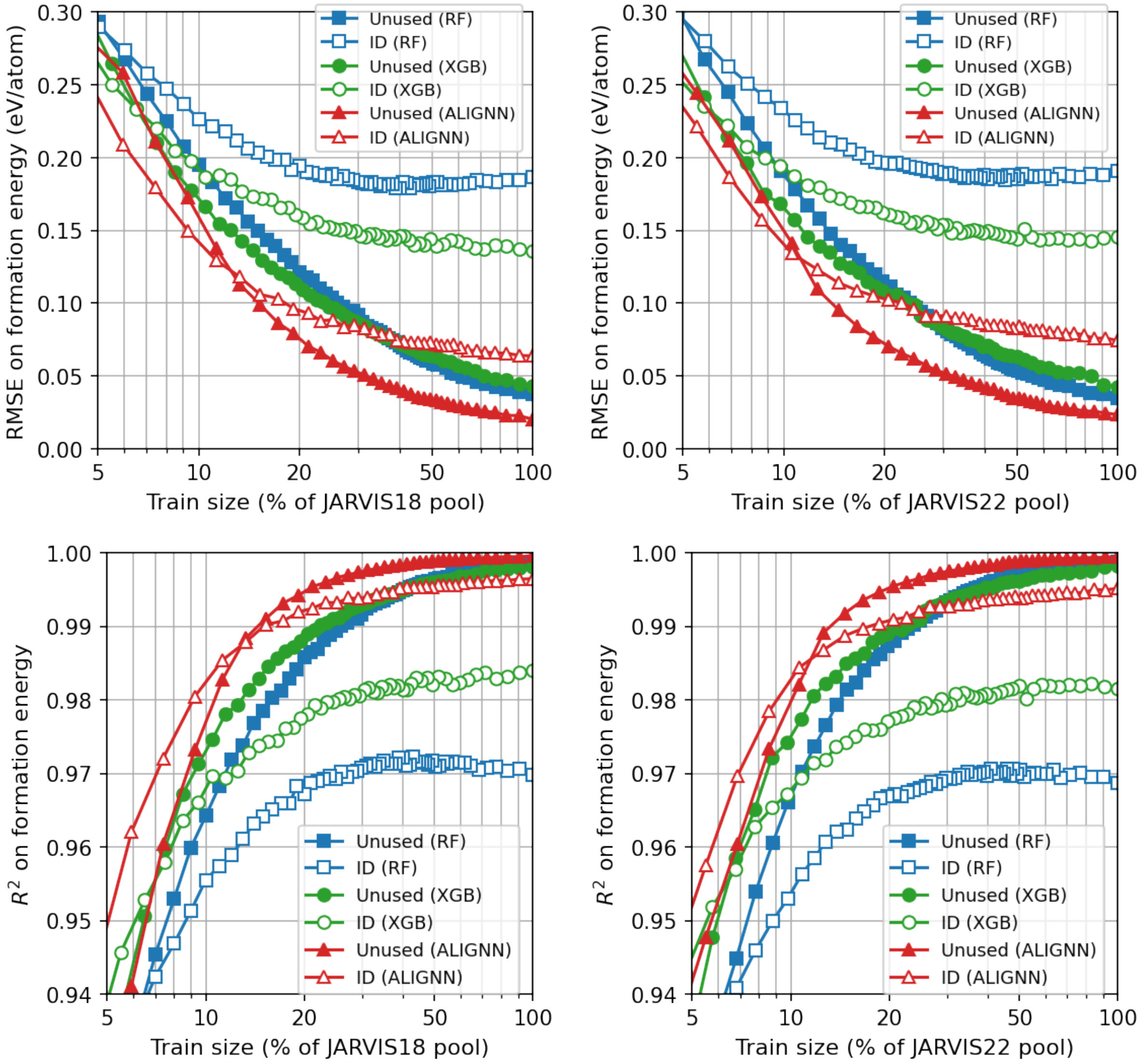

Supplementary Figure 7. Root mean square error (1st row) and R^2 (2nd row) on the unused data for the JARVIS18 (1st column) and JARVIS22 (2nd column) formation energy prediction. RF: random forest. XGB: XGBoost. ALIGNN: atomistic line graph neural network.

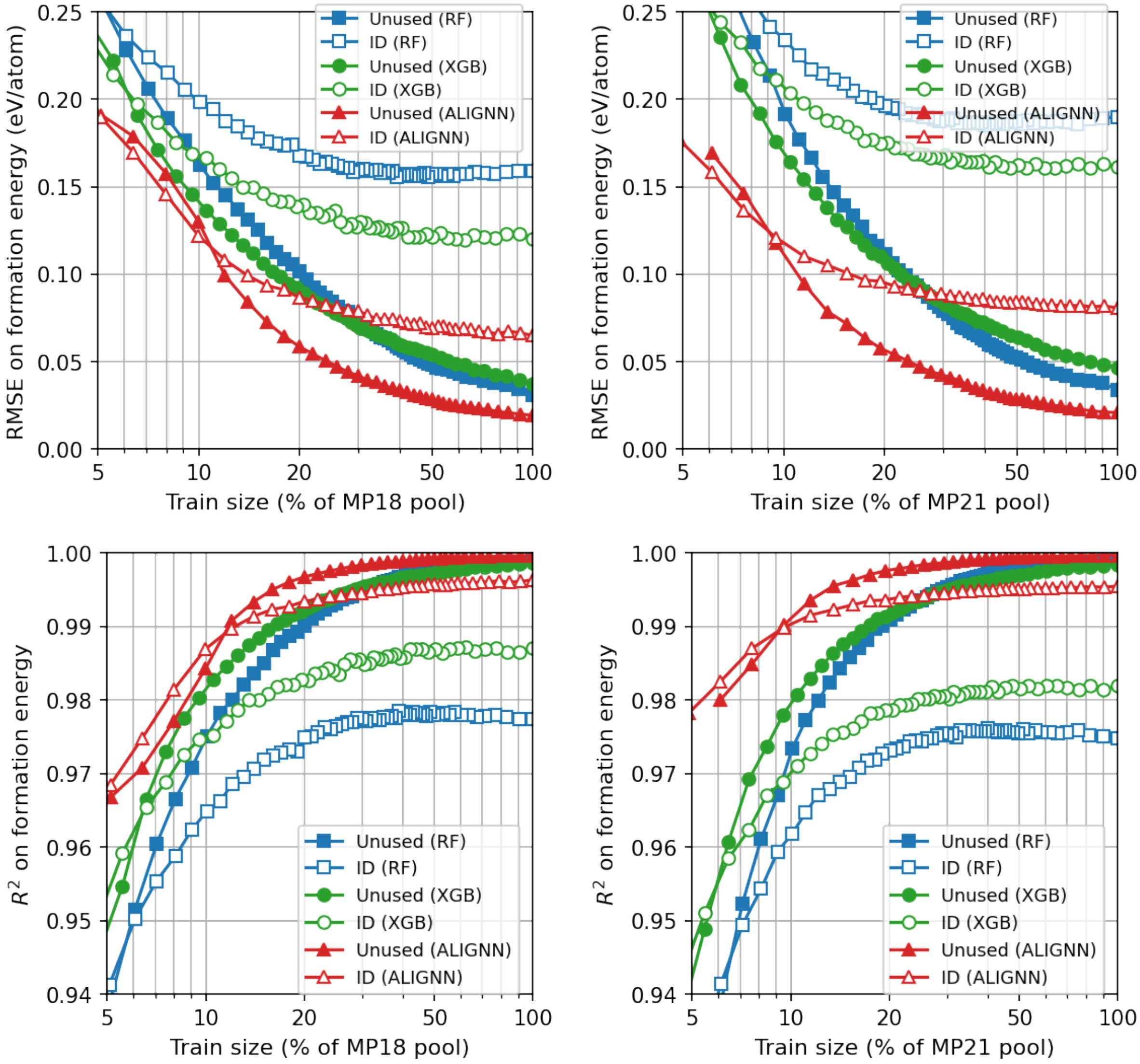

Supplementary Figure 8. Root mean square error (1st row) and R^2 (2nd row) on the unused data for the MP18 (1st column) and MP21 (2nd column) formation energy prediction. RF: random forest. XGB: XGBoost. ALIGNN: atomistic line graph neural network.

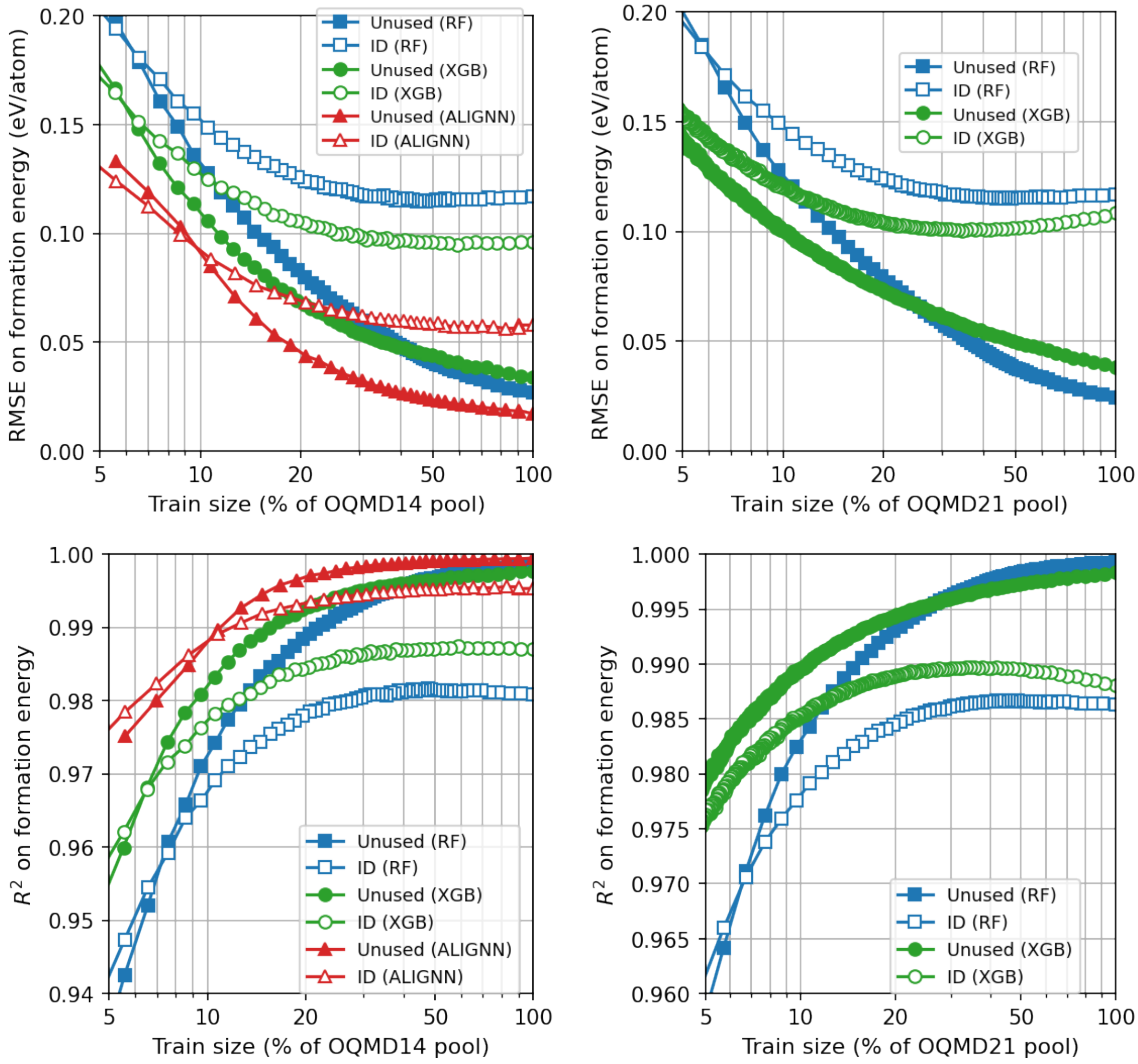

Supplementary Figure 9. Root mean square error (1st row) and R^2 (2nd row) on the unused data for the OQMD14 (1st column) and OQMD21 (2nd column) formation energy prediction. RF: random forest. XGB: XGBoost. ALIGNN: atomistic line graph neural network.

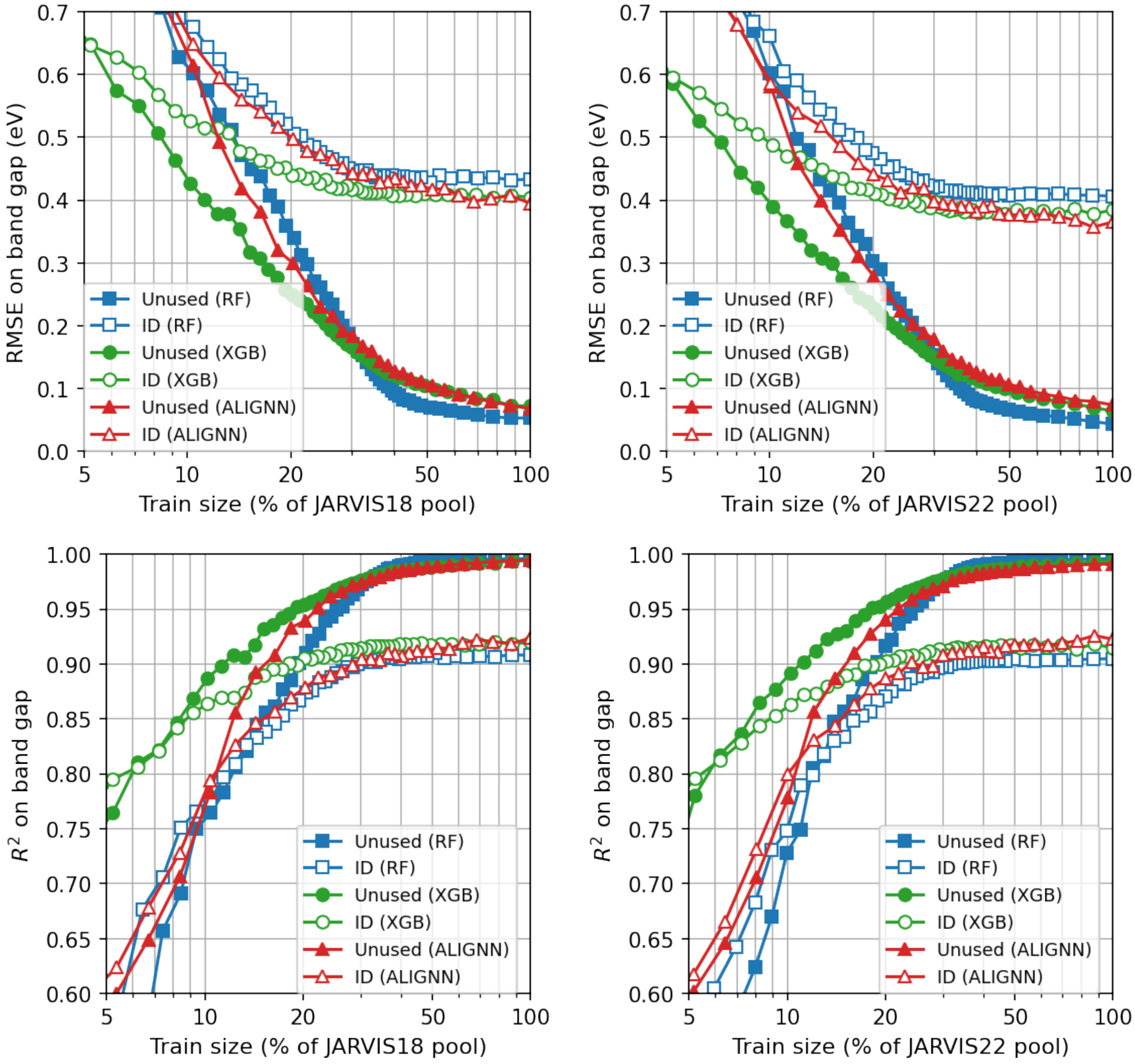

Supplementary Figure 10. Root mean square error (1st row) and R^2 (2nd row) on the unused data for the JARVIS18 (1st column) and JARVIS22 (2nd column) band gap prediction. RF: random forest. XGB: XGBoost. ALIGNN: atomistic line graph neural network.

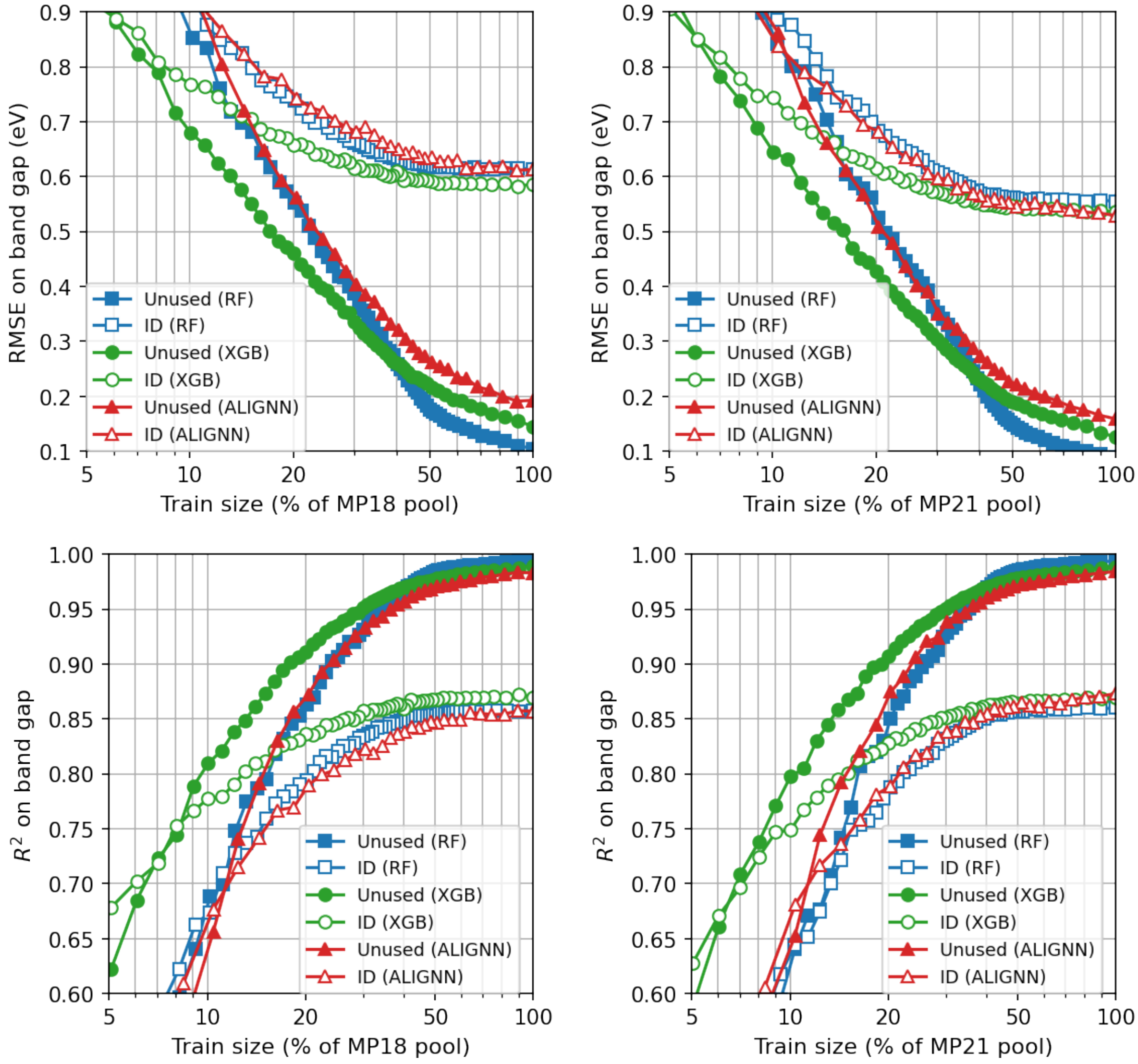

Supplementary Figure 11. Root mean square error (1st row) and R^2 (2nd row) on the unused data for the MP18 (1st column) and MP21 (2nd column) band gap prediction. RF: random forest. XGB: XGBoost. ALIGNN: atomistic line graph neural network.

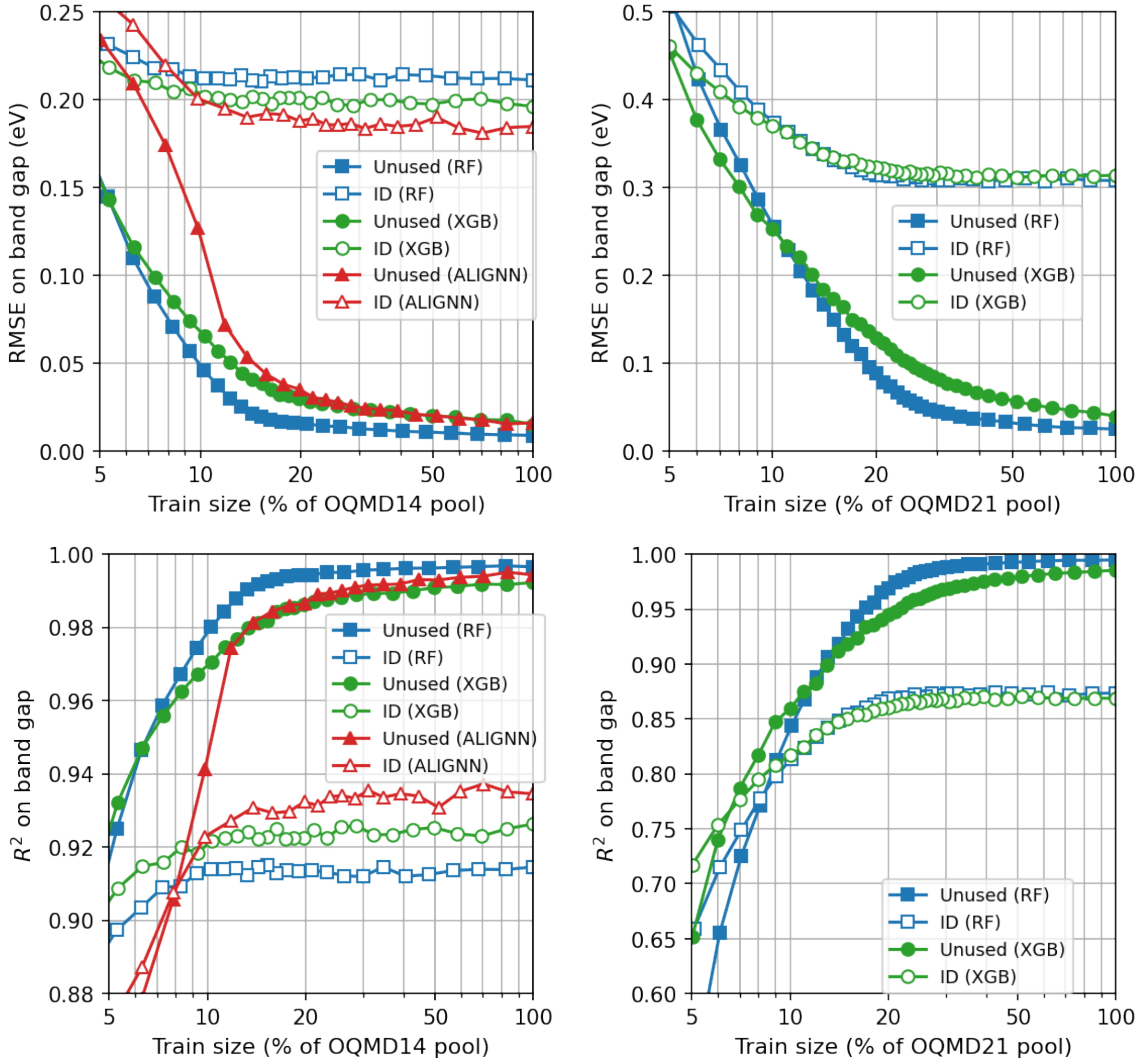

Supplementary Figure 12. Root mean square error (1st row) and R^2 (2nd row) on the unused data for the OQMD14 (1st column) and OQMD21 (2nd column) band gap prediction. RF: random forest. XGB: XGBoost. ALIGNN: atomistic line graph neural network.

C. Performance on OOD test set

To quantify the degree of the performance degradation due to the distribution shift, we train the models using the entire pool of the older datasets (JARVIS18, MP18, or OQMD14), and test their performance on the hold-out ID test sets of the older datasets, and on the OOD test sets in the newer datsets (JARVIS22, MP21, or OQMD21). The OOD performance (Root mean square error and R^2) of the formation energy models is shown in Fig. 13 for the JARVIS, MP, and OQMD datasets. The OOD performance (root mean square error and R^2) of the band gap models is shown in Fig. 14 for the JARVIS, MP, and OQMD datasets. The ratios of the ID root mean square error to the OOD root mean square error for the models trained on 100 % of the pool are given in Table I.

TABLE I. The ratio of the root mean square error on the OOD test set to that on the ID test set using the full models (namely, trained on the entire pool).

Database	Property	RF	XGB	ALIGNN
JARVIS	formation energy	1.5	1.6	2.0
	band gap	1.1	1.1	1.2
MP	formation energy	4.0	4.2	7.3
	band gap	1.3	1.3	1.3
OQMD	formation energy	3.1	3.1	3.2
	band gap	2.9	3.1	3.2

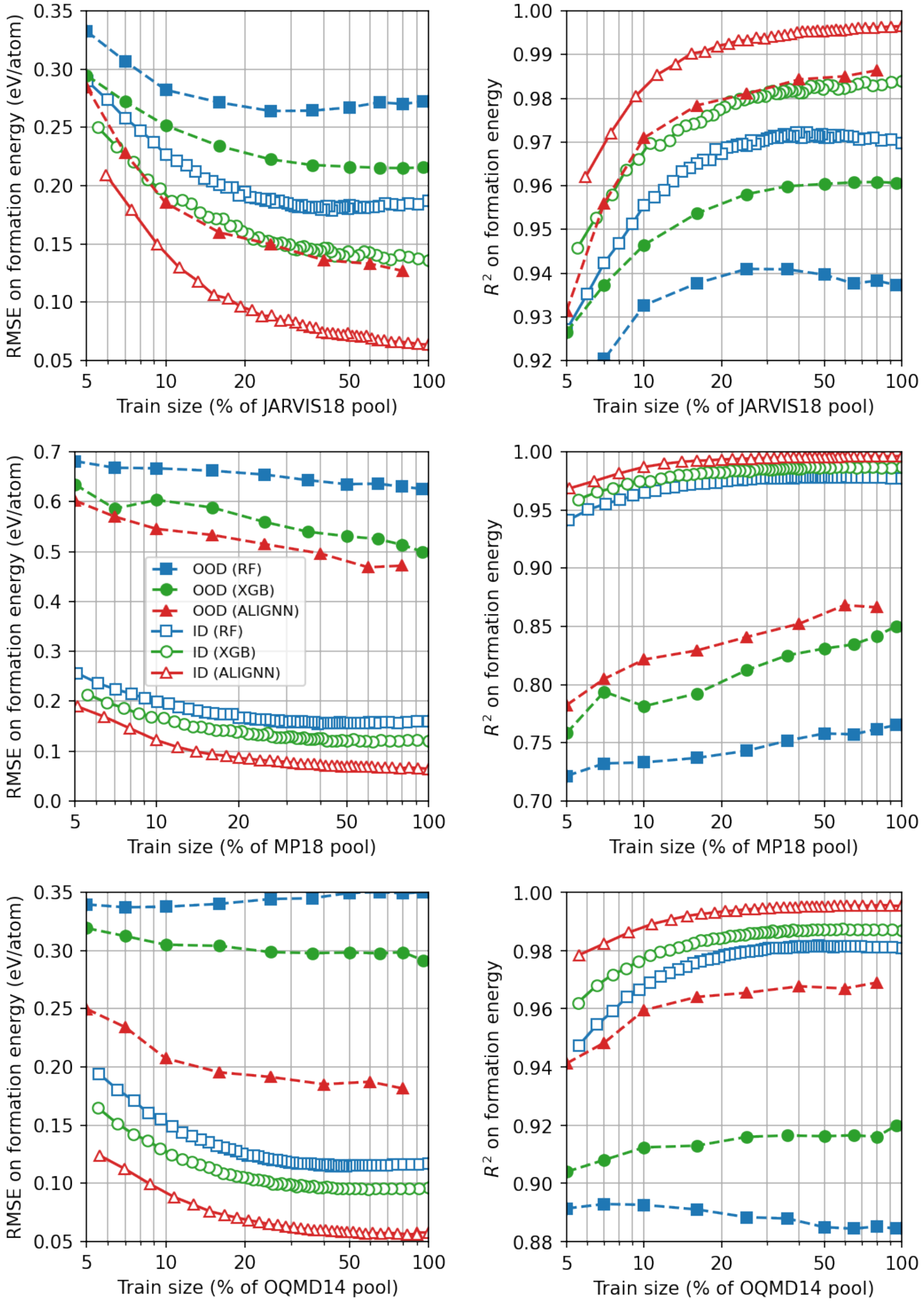

Supplementary Figure 13. OOD performance of the JARVIS, MP, and OQMD formation energy predictions. RF: random forest. XGB: XGBoost. ALIGNN: atomistic line graph neural network.

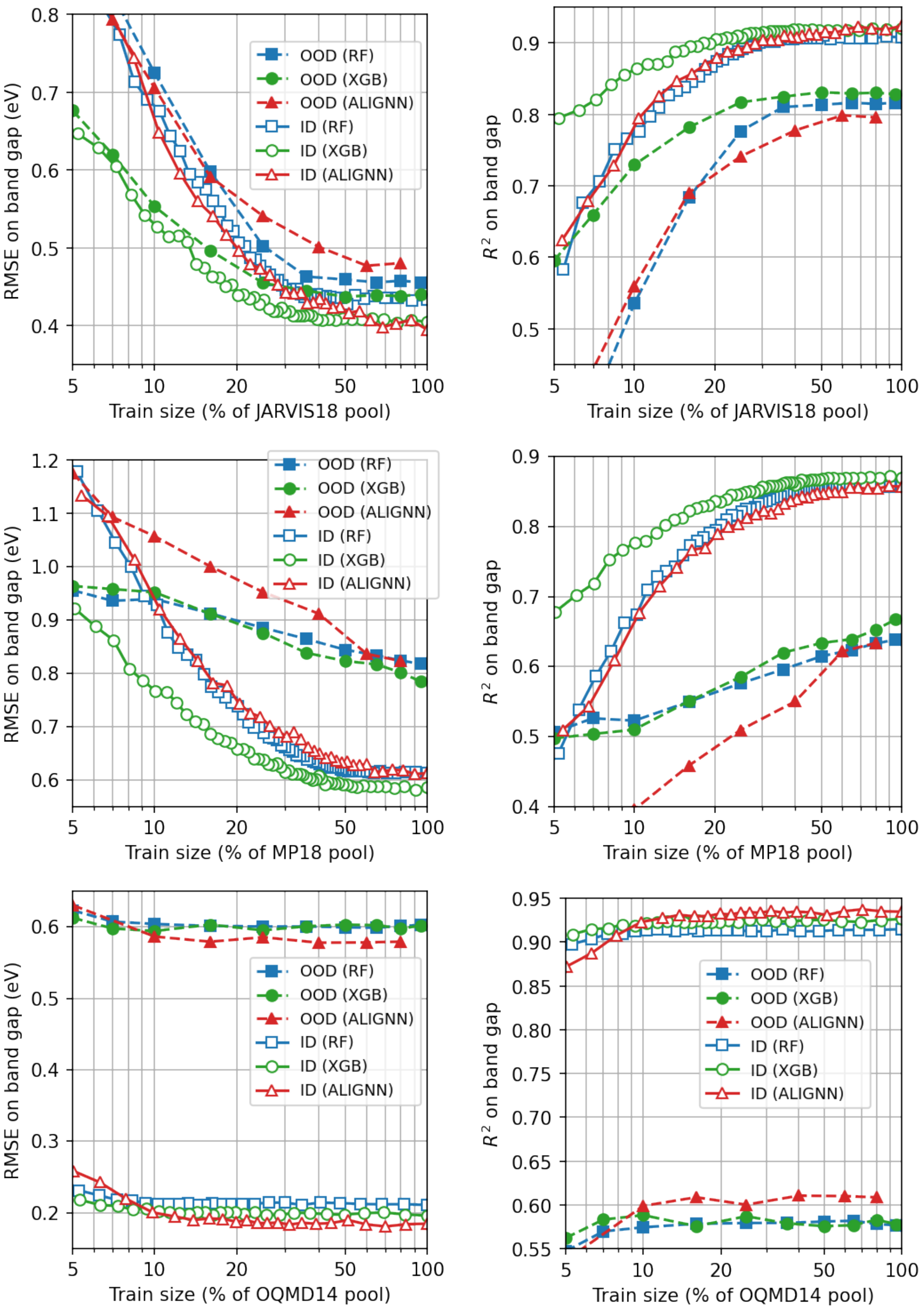

Supplementary Figure 14. OOD performance of the JARVIS, MP, and OQMD band gap predictions. RF: random forest. XGB: XGBoost. ALIGNN: atomistic line graph neural network.

II. LABEL DISTRIBUTION OF THE PRUNED DATA SETS

The pruned data exhibits a distribution different from the original distribution of S_0. To demonstrate this point, we show the label distributions of the XGB-pruned formation energy data in Fig. 15 and band gap data in Fig. 16. Compared to the original distributions (100 % of the pool), the distributions of the pruned data (50 %, 20 %, and 5 % of the pool) are increasingly skewed towards less stable materials which are underrepresented in the original distribution. Similarly for the band gap data, a large portion of the materials have a band gap close to zero in the original distribution, whereas the distributions of the pruned data are skewed towards materials with larger band gaps.

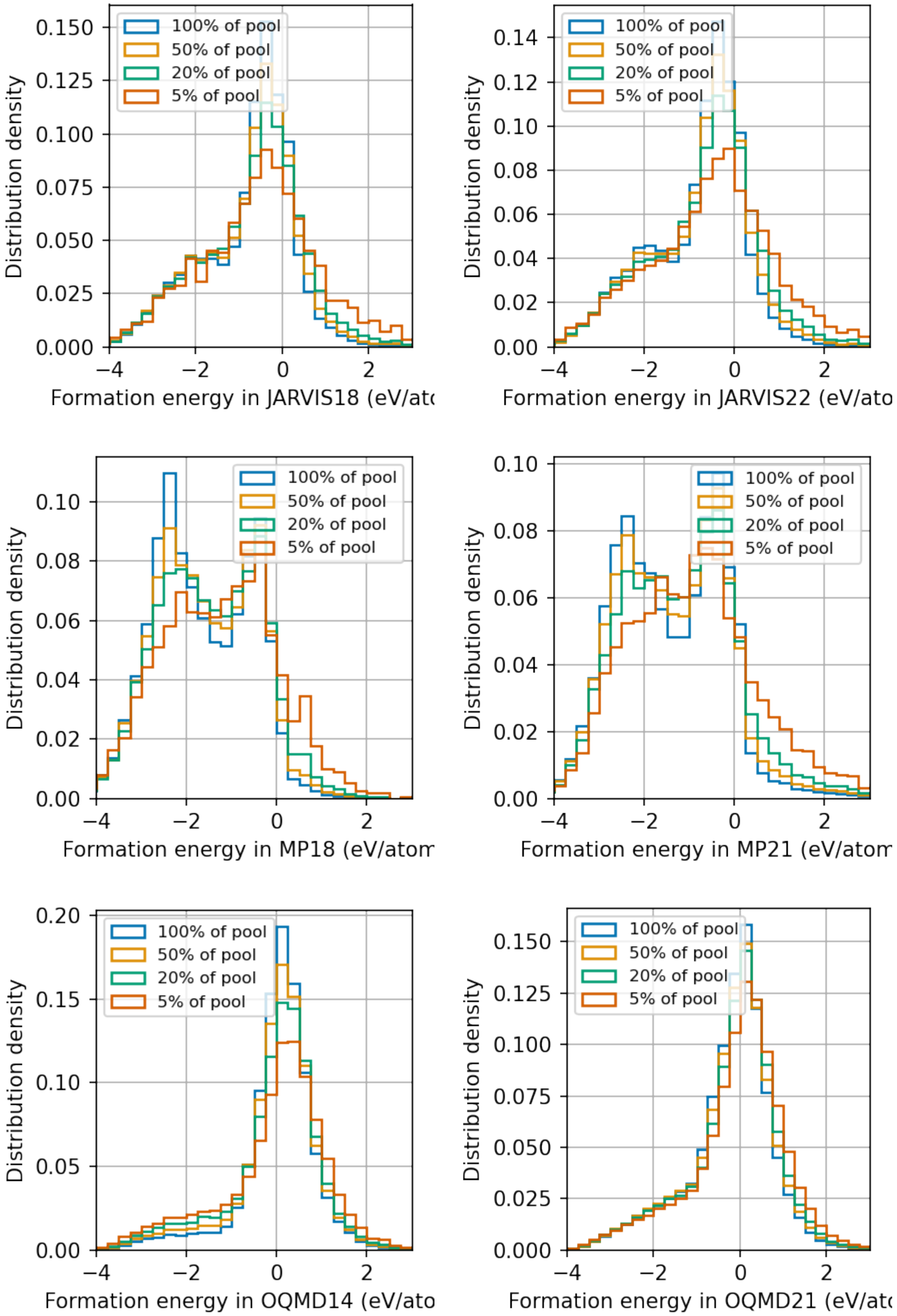

Supplementary Figure 15. Label distribution of the XGBoost-pruned formation energy data. In each figure, the distributions of the training sets accounting for 100 % to 5 % of the pool are shown.

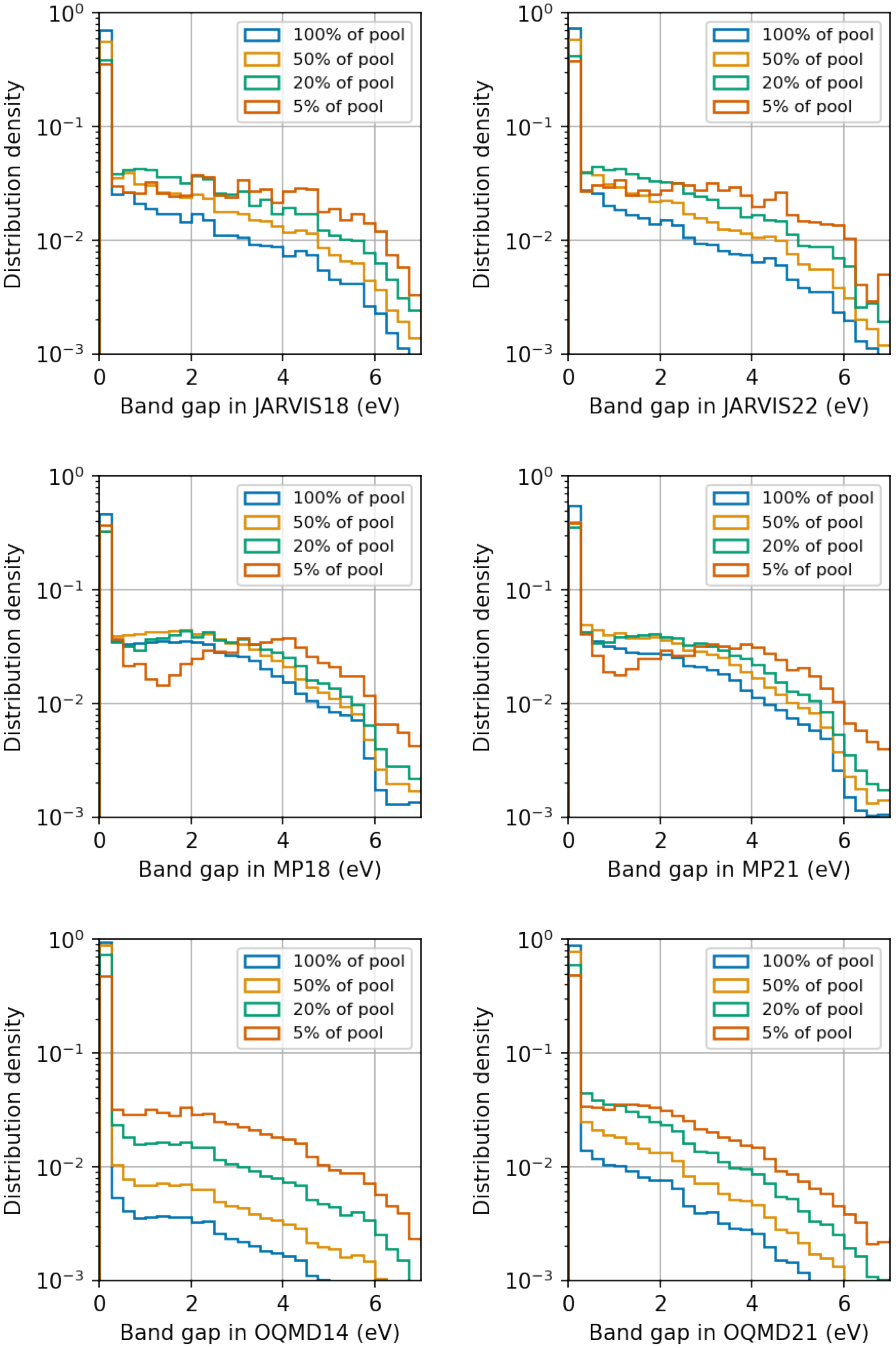

Supplementary Figure 16. Label distribution of the XGBoost-pruned band gap data. In each figure, the distributions of the training sets accounting for 100 % to 5 % of the pool are shown. Please note that the distribution density is in the logarithmic scale.

III. TRANSFERABILITY OF PRUNED MATERIAL SETS

A. Transferability between ML models

To investigate the transferability of material sets between ML architectures, we evaluate the ID performance of the XGB and RF models trained on the data pruned by the RF and XGB models, respectively. For the formation energy prediction, the ID performance of the XGB and RF models are shown in Fig. 17 and Fig. 18, respectively. For the band gap prediction, the ID performance of the XGB and RF models are shown in Fig. 19 and Fig. 20, respectively.

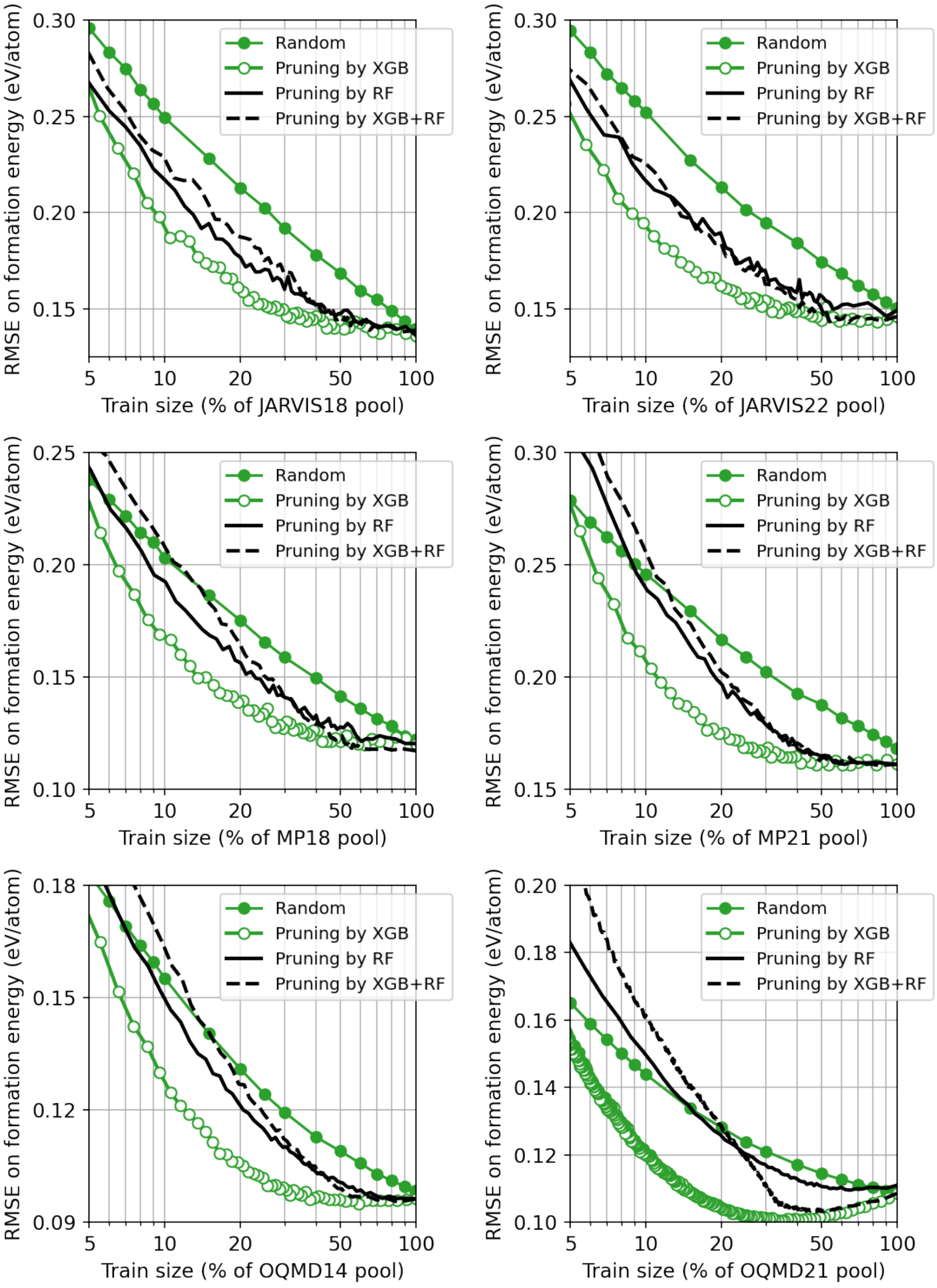

Supplementary Figure 17. ID performance of the XGB models for JARVIS18, JARVIS22, MP18, MP21, OQMD14, and OQMD21 formation energy datasets. For each dataset, the root mean square errors obtained by training the XGB models on the randomly selected data, the data pruned by the XGB models, the data pruned by the RF models, and the data jointly pruned by the XGB and RF models are shown. RF: random forest. XGB: XGBoost.

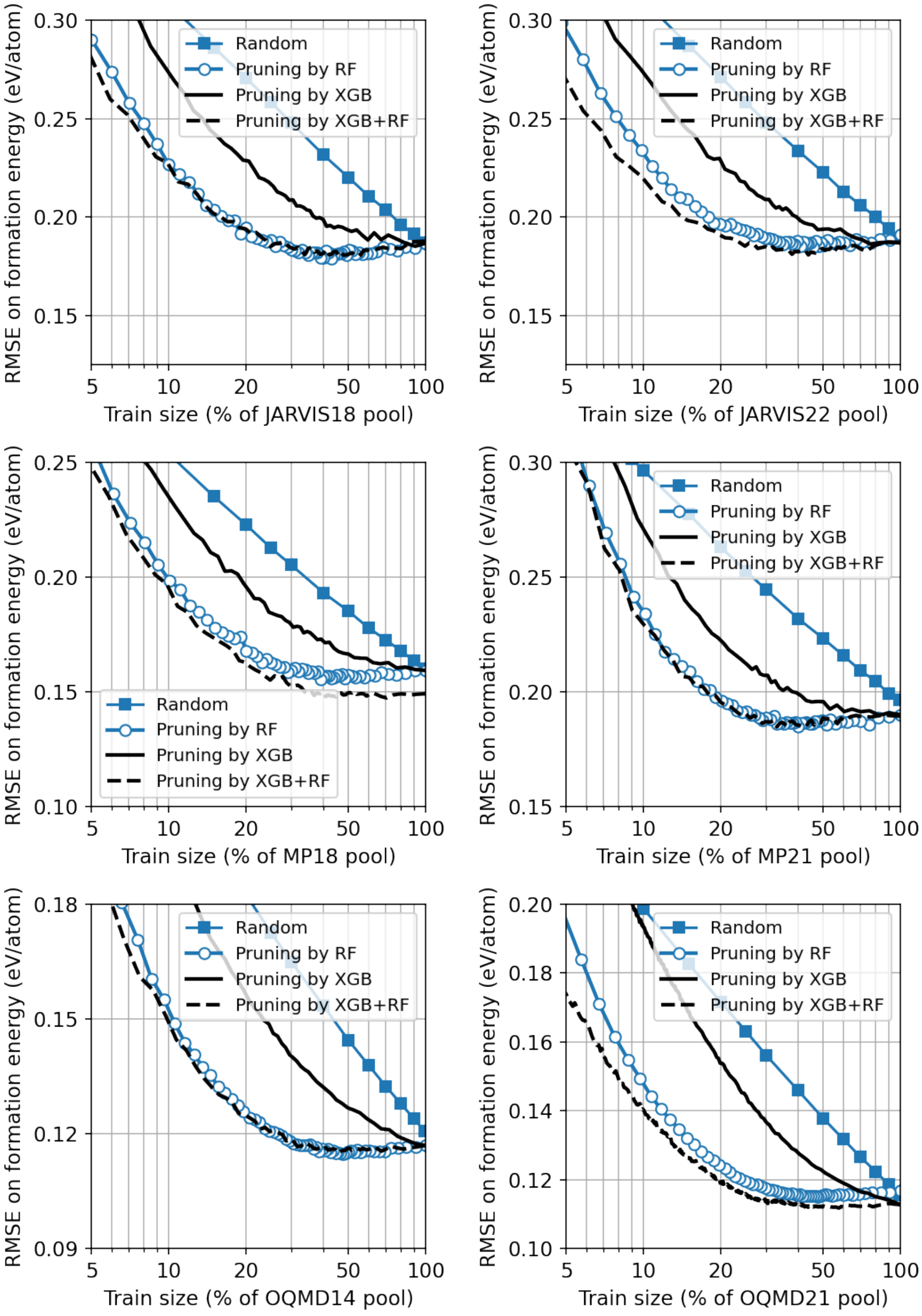

Supplementary Figure 18. ID performance of the RF models for JARVIS18, JARVIS22, MP18, MP21, OQMD14, and OQMD21 formation energy datasets. RF: random forest. XGB: XGBoost. For each dataset, the root mean square errors obtained by training the RF models on the randomly selected data, the data pruned by the RF models, the data pruned by the XGB models, and the data jointly pruned by the XGB and RF models are shown.

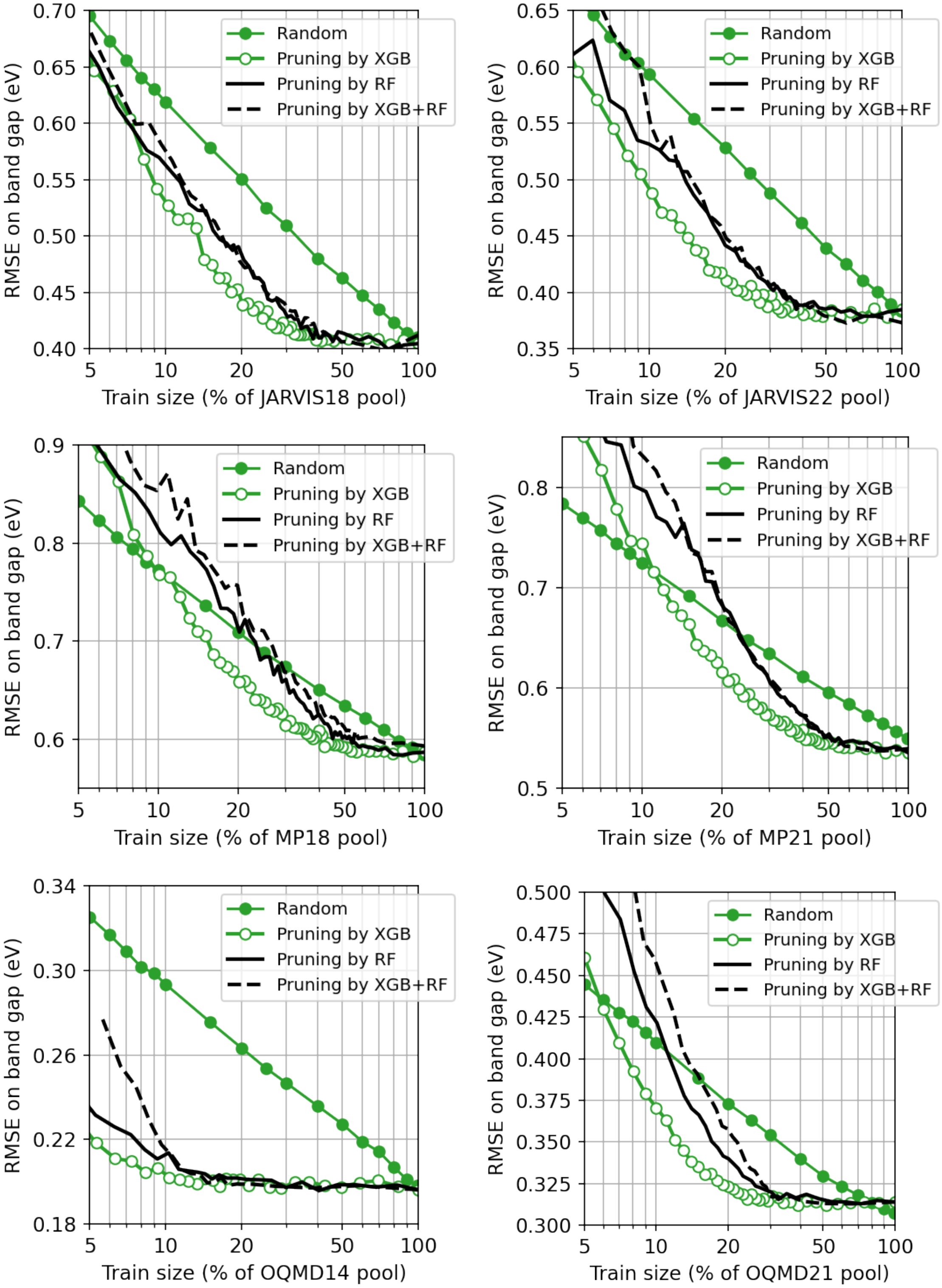

Supplementary Figure 19. ID performance of the XGB models for JARVIS18, JARVIS22, MP18, MP21, OQMD14, and OQMD21 band gap datasets. RF: random forest. XGB: XGBoost. For each dataset, the root mean square errors obtained by training the XGB models on the randomly selected data, the data pruned by the XGB models, the data pruned by the RF models, and the data jointly pruned by the XGB and RF models are shown.

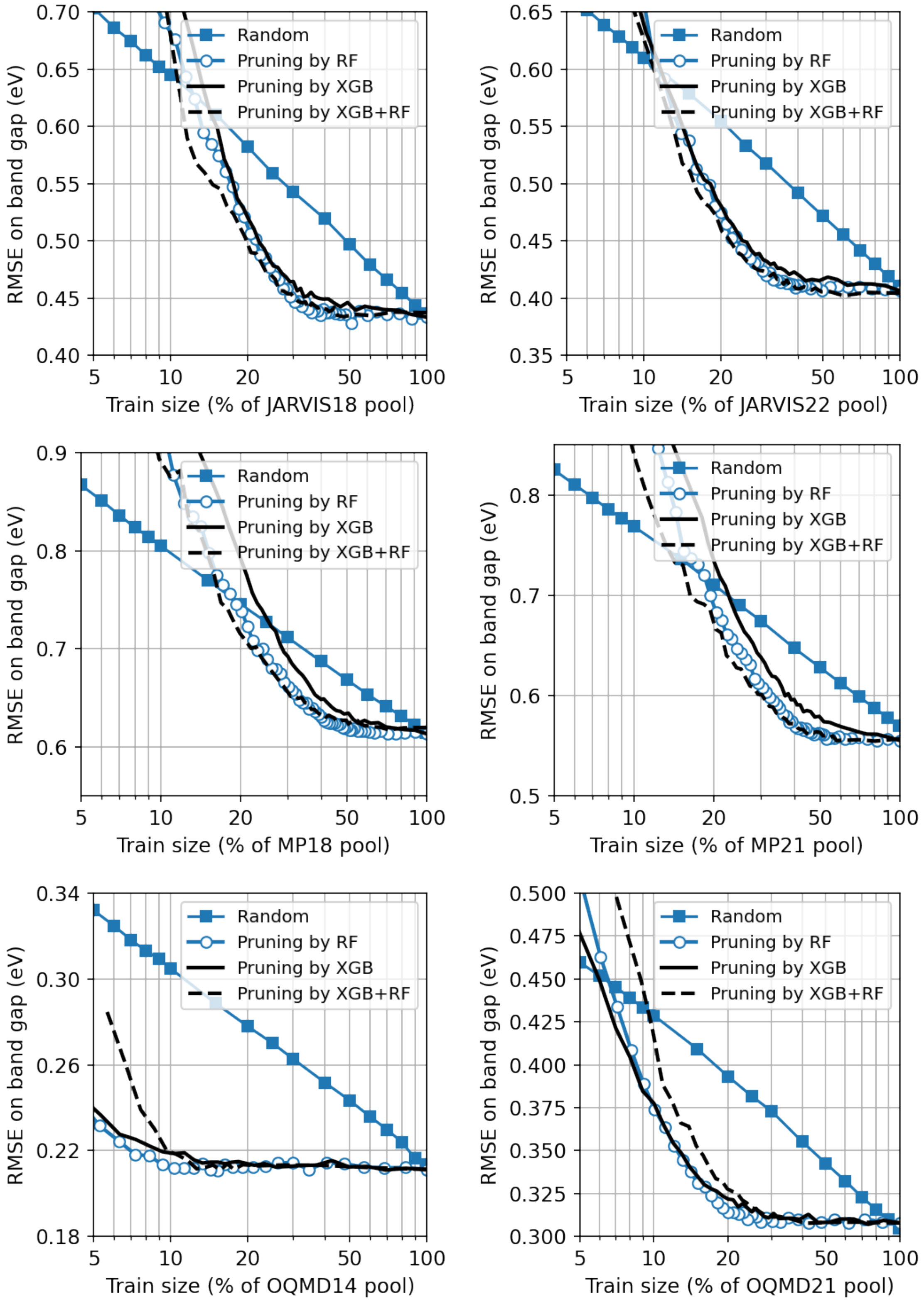

Supplementary Figure 20. ID performance of the RF models for JARVIS18, JARVIS22, MP18, MP21, OQMD14, and OQMD21 band gap datasets. RF: random forest. XGB: XGBoost. For each dataset, the root mean square errors obtained by training the RF models on the randomly selected data, the data pruned by the RF models, the data pruned by the XGB models, and the data jointly pruned by the XGB and RF models are shown.

B. Transferability between material properties

To investigate the transferability of material sets between material properties, we first identify the material sets from the formation energy data pruning procedure of a given ML model (XGB or RF), and use the corresponding band gap data of these material sets to train the given ML model. The resulting ID performance for JARVIS18, MP21, and OQMD14 band gap datasets is shown in Fig. 21. While the band gap models trained on the data identified from the formation energy data pruning still outperform the models trained on randomly sampled data but by a small degree, and perform less better the models trained on the data from the band gap data pruning, suggesting a limited transferability of the informative material sets between the formation energy and band gap data.

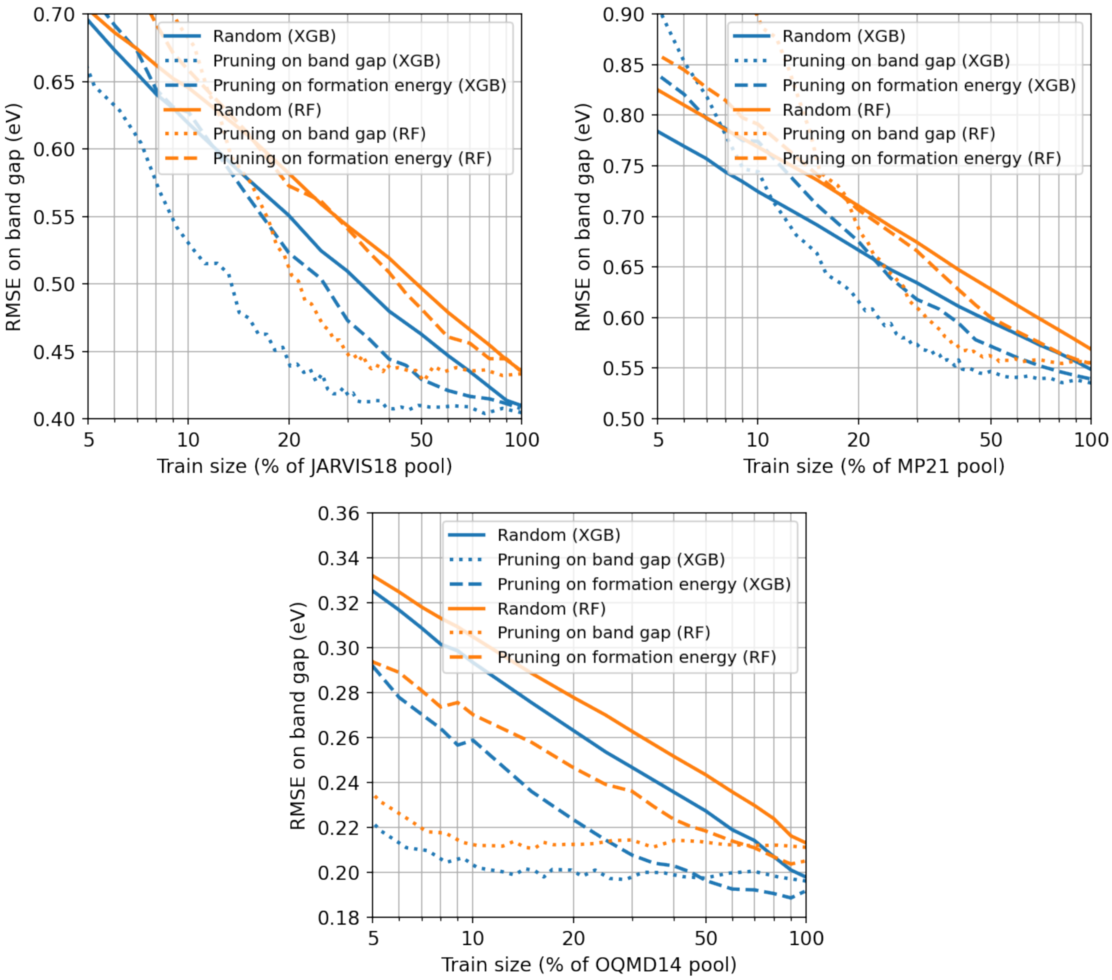

Supplementary Figure 21. ID performance of the XGB and RF models for JARVIS18, MP21, and OQMD14 band gap datasets. RF: random forest. XGB: XGBoost. For each dataset and each model, the root mean square errors by training on the randomly selected materials, the materials from the band gap data pruning, and the materials from the formation energy data pruning are shown.

IV. UNCERTAINTY-BASED ACTIVE LEARNING

To demonstrate the feasibility of building smaller but informative datasets, we use uncertainty-based active learning algorithms to grow the JARVIS22, MP21 and OQMD14 datasets from scratch. Three uncertainty measures are considered: The first one (RF-U) is based on the uncertainty of the RF model and is calculated as the difference between the 95th and 5th percentile of the tree predictions in the forest. The second one (XGB-U) is based on the uncertainty of the XGB model using an instance-based uncertainty estimation

for gradient-boosted regression trees developed in Ref. [1]. The third one (QBC) is based on the query by committee, where the uncertainty is taken as the difference between the RF and XGB predictions. Fig. 22 and 23 show the resulting ID performance of the XGB and RF models.

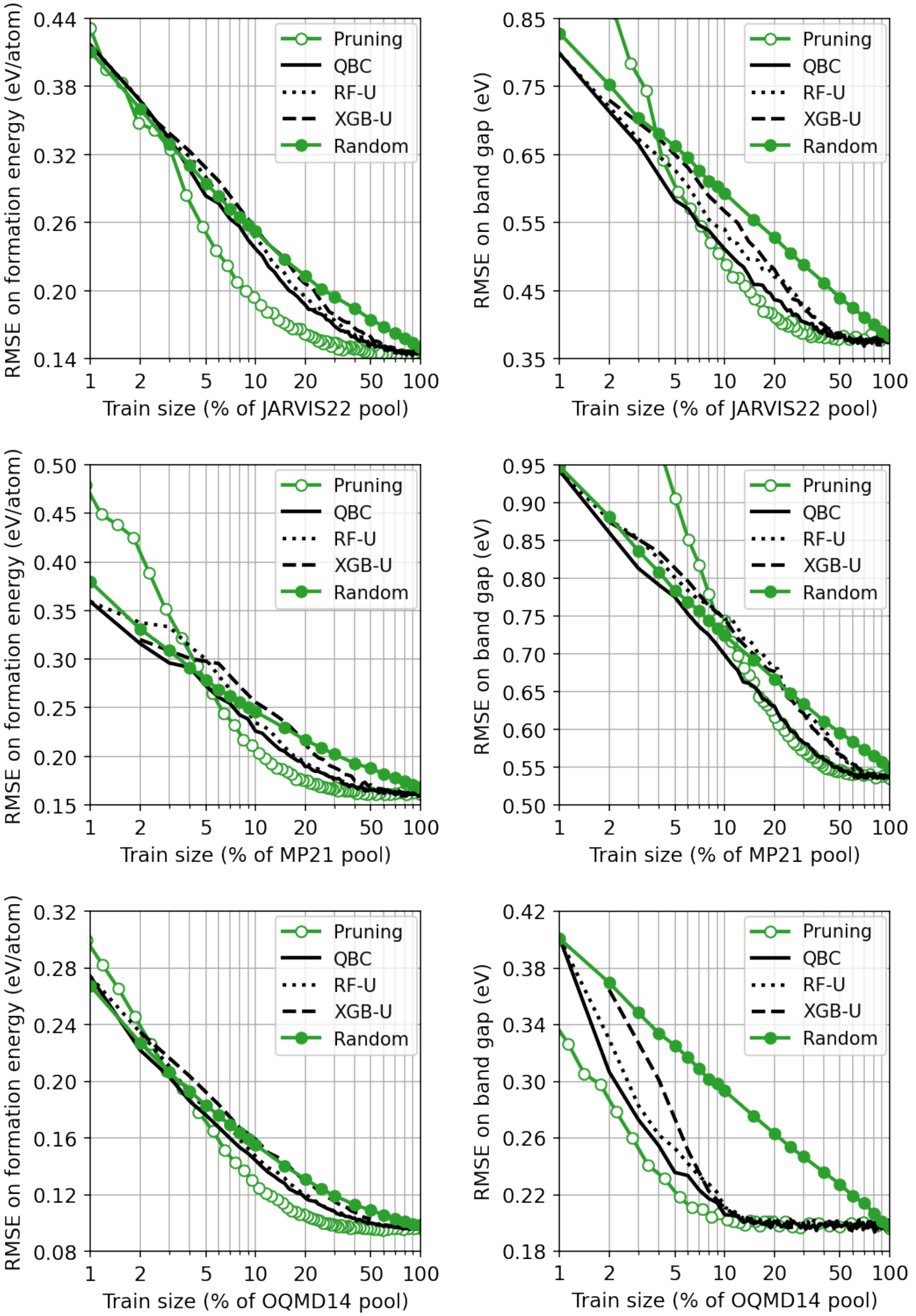

Supplementary Figure 22. ID performance of the XGB models for JARVIS22, MP21, and OQMD14 formation energy and band gap datasets, using the uncertainty-based active learning algorithms. RF: random forest. XGB: XGBoost. QBC: query by committee, RF-U: random forest uncertainty, XGB-U: XGBoost uncertainty. For each dataset, we show the root mean square errors

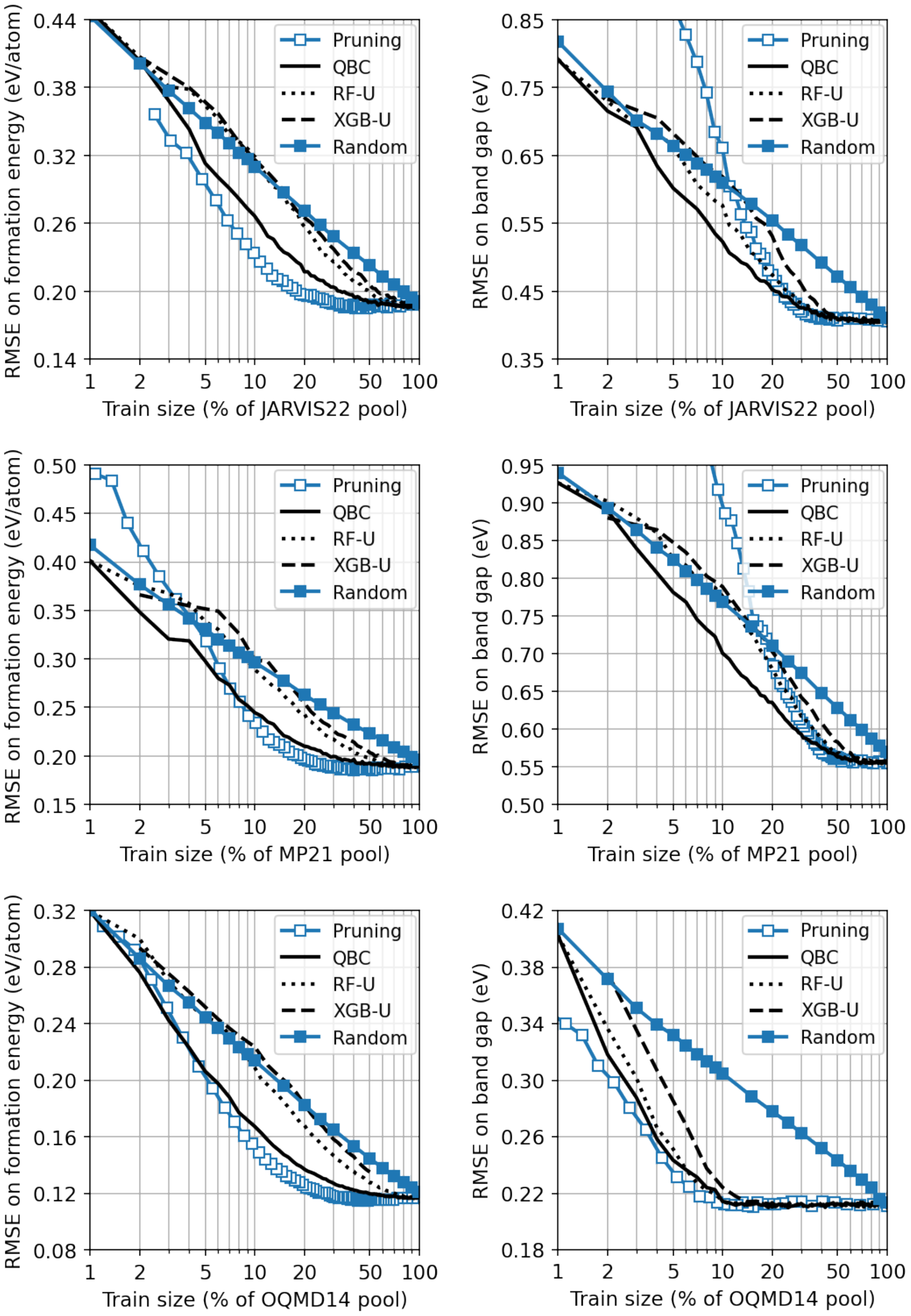

Supplementary Figure 23. ID performance of the RF models for JARVIS22, MP21, and OQMD14 formation energy and band gap datasets, using the uncertainty-based active learning algorithms. RF: random forest. XGB: XGBoost. QBC: query by committee, RF-U: random forest uncertainty, XGB-U: XGBoost uncertainty. For each dataset, we show the root mean square errors obtained

V. STATISTICAL OVERLAP BETWEEN THE TRAINING POOL AND OOD DATA

In Fig. 4 of the main text, we demonstrate that there is a strong OOD performance degradation for the formation energy prediction for the JARVIS and MP datasets, and the band gap prediction for the OQMD dataset. To better understand the statistical overlap between the older dataset and the OOD data in the newer dataset, we provide below an analysis in the feature space using Uniform Manifold Approximation and Projection (UMAP), which is a stochastic and non-linear dimensionality reduction algorithm that preserves the data's local and global structure [2]. This technique was previously used to analyze a similar OOD performance degradation for the formation energy prediction of metallic alloys in the MP dataset in our recent work [3], and interested reader is referred to the work therein for an extended discussion.

Here we take the JARVIS database as an example to describe how the UMAP analysis is done. We first perform the standardization of Matminer-extracted features over the whole JARVIS22 dataset. Then we sequentially drop highly correlated features using a Pearson correlation threshold of 0.7. Next, the remaining features are used as inputs of UMAP to create the embeddings for the whole JARVIS22 dataset. We use n_neighbors=300 and the default values for other UMAP hyperparameters. We visualize the data in the UMAP embedding with three subplots (see the 1st row in Supplementary Figure 24): the first subplot shows the JARVIS18 data on top of the new data in JARVIS22 (OOD data); the second subplot shows the JARVIS18 only; the third subplot show the OOD data, which are colored by the prediction errors of the XGBoost model trained on the whole JARVIS18 dataset. The results for the MP and OQMD datasets are obtained similarly (see the 2nd and 3rd rows in the 1st row in Supplementary Figure 24).

For the JARVIS database, the OOD data is almost covered by the training data in the UMAP-projected feature space. For the MP database, the OOD data also largely overlap with the training data, with only two small clusters less well covered by the training data. For the OQMD database, there is a relatively large portion of OOD data lying beyond the region covered by the training data. The degree of the overlap might explain to some extent the degree of the OOD performance degradation: for instance, the overlap for the JARVIS database almost reaches 100 %, which may explain why its OOD performance degradation

is the least significant among the three datasets (the Fig. 4 of the main text). However, the subplots showing the OOD data colored by their prediction errors indicate that the OOD data with large prediction errors also occur in the region well covered by the training data. This suggests that more in-depth analysis is needed in the future work to better understand the correlation between the overlap in the feature space and the prediction errors.

[1] J. Brophy and D. Lowd, in *Advances in Neural Information Processing Systems* (2022).

[2] L. McInnes, J. Healy, N. Saul, and L. Großberger, J. Open Source Softw. **3**, 861 (2018).

[3] K. Li, B. DeCost, K. Choudhary, M. Greenwood, and J. Hattrick-Simpers, npj Computational Materials **9**, 55 (2023).

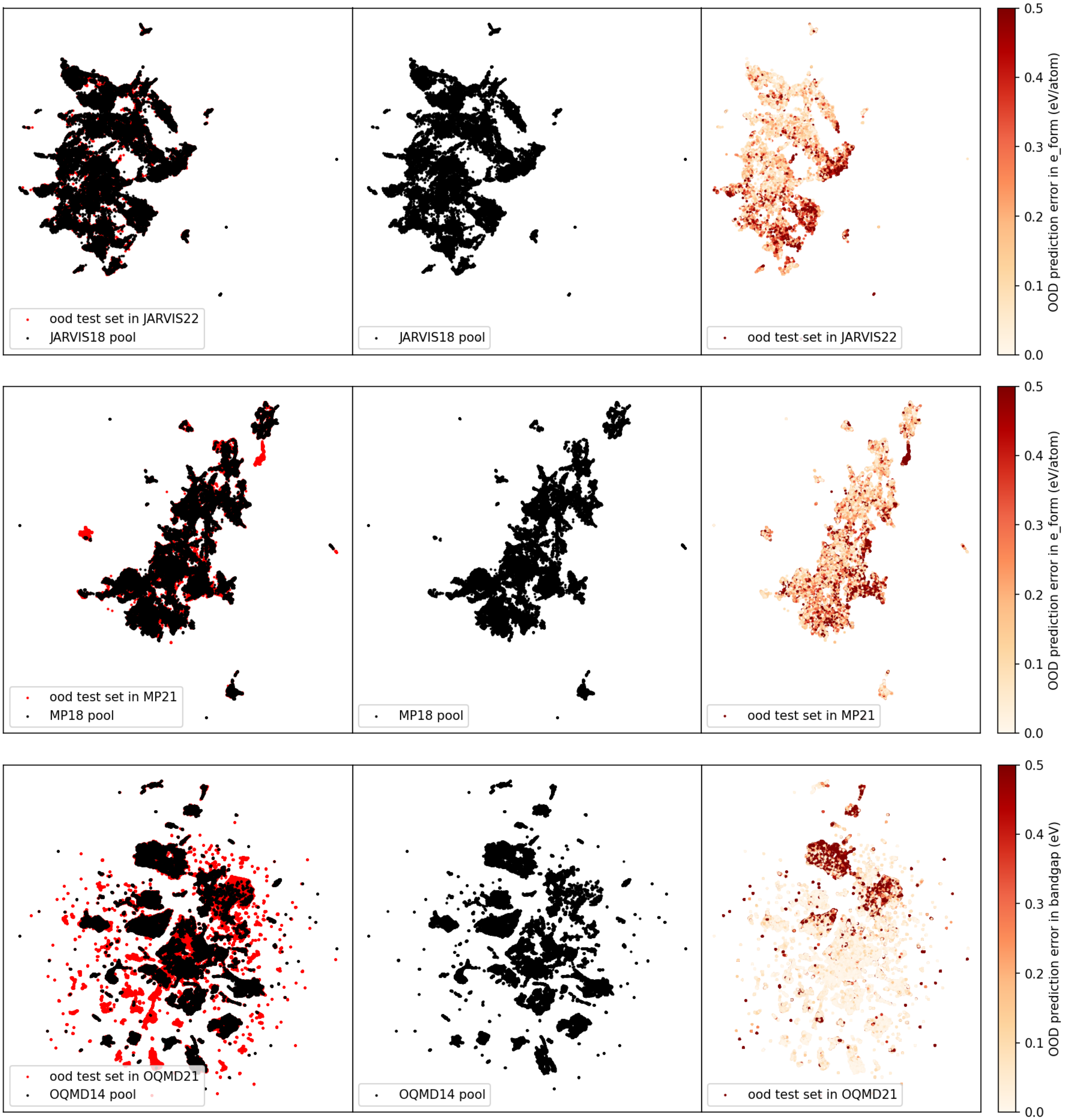

Supplementary Figure 24. Uniform Manifold Approximation and Projection (UMAP) in the feature space.
