# Supplementary material for: Exploiting redundancy in large materials datasets for efficient machine learning with less data
Source: Nat Commun. 2023 Nov 10;14:7283. doi: 10.1038/s41467-023-42992-y (PMC10638383; doi:10.1038/s41467-023-42992-y)
Supplement: Supplementary file 2 — Peer Review File [file 41467_2023_42992_MOESM2_ESM.pdf]

nature portfolio

Peer Review File

Exploiting redundancy in large materials datasets for efficient machine learning with less data

Open Access This file is licensed under a Creative Commons Attribution 4.0 International License, which permits use, sharing, adaptation, distribution and reproduction in any medium or format, as long as you give appropriate credit to the original author(s) and the source, provide a link to the Creative Commons license, and indicate if changes were made. In the cases where the authors are anonymous, such as is the case for the reports of anonymous peer reviewers, author attribution should be to 'Anonymous Referee' followed by a clear attribution to the source work. The images or other third party material in this file are included in the article's Creative Commons license, unless indicated otherwise in a credit line to the material. If material is not included in the article's Creative Commons license and your intended use is not permitted by statutory regulation or exceeds the permitted use, you will need to obtain permission directly from the copyright holder. To view a copy of this license, visit http://creativecommons.org/licenses/by/4.0/.

REVIEWER COMMENTS

Reviewer #1 (Remarks to the Author):

Li et al. present atimely and highly relevant contribution for the ML for Materials Science community and put a great highlight on the enhancement of sampling efficiency of active learning. In fact I discussed something very similar in my machine learning lecture this week on the QM9 dataset and kernel regression methods. The paper is overall very well written and presented concisely in such a way that I have all but one minor comment relating to the literature cited and putting this into context: Xi et al. present in 10.1038/s41586-019-1540-5 the issue relating to "Anthropogenic biases in chemical reaction data" and how it "hinders exploratory inorganic synthesis" and this paper is an excellent contribution from the field of organic chemistry. Then the paper by Wang et al. is IMHO not the (only) one to be cited here as both 10.1038/s41524-021-00656-9 and 10.1039/C9SC05999G predate it and demonstrate across multiple materials science domains. There is even a github repository to measure both the enhancement factor and acceleration factor - two measures that express how much active learning "accelerates".

Reviewer #2 (Remarks to the Author):

Data redundancy in machine learning (ML) in materials science is investigated. This is mostly done in the context of ML model generation and to a lesser extend for saving time and cost in data labelling. For the study, 3 different publicly available datasets in computational materials science (JARVIS, MP, and OQMD) were used. These were partly chosen, because recent updates extend earlier versions of the dataset and make it possible to quantify out-of-distribution errors. Different ML models are trained and then reduced in size with pruning or active learning. By comparing to the largest ML models, the authors find that low in-distribution errors can already be achieved with much smaller datasets pointing towards redundancy in the training set. The out-of-distribution error, however, remains high and suggests that the extensions to the datasets have reduced overlap with the original data.

The idea of applying a redundancy analysis to these nested datasets is very good and the out-of-distribution results are really insightful. Apart from that, the study does not offer much that is new. Many statements made in the manuscript are obvious. Challenging the “bigger is better” philosophy is of course appealing for a Nature-type article, but one should bear in mind that many datasets in materials science (the ones used by the authors included) are not primarily generated for training regression models. Secondly, if maximally compact datasets are generated with active learning, as the authors advocate, one has to be wary of the reusability. Due to the nature of their assembly, active learning datasets often exhibit a bias towards the target properties they were generated with and might not generalise for other purposes. This discussion is completely absent from the manuscript.

Furthermore, I am doubtful that the active learning results presented in Figure 5 are correct. In the Methods section, the authors state “During the active learning process, the training set is initially constructed by randomly sampling 1% to 2% of the pool […]”. This would mean that for the smallest training set size of 1 or 2% of the full training set, random sampling and active learning should agree with some statistical fluctuations. Unfortunately, this low range of the training set size is cut off in Figure 5. But it would appear as though the RMSE of the formation energy (panel a) is actually higher in active learning than in random sampling and for the band gap it might be lower. I strongly encourage the authors to check their calculations and to show these consistency checks in the paper or at least the supplementary information. I would also strongly recommend to average the RMSE over several runs to remove statistical fluctuations from the learning curves.

I am furthermore surprised that the authors achieve large data savings with their active learning models. The in-distribution test set is drawn randomly from the whole dataset. The test set

distribution and the training set distribution should therefore be very similar and random sampling should be the optimal strategy to minimise a global quantity like the RMSE.

Considering these aspects, I cannot recommend the manuscript for publication in Nature Communication. More detailed comments are given below:

- Overall the article is a bit repetitive as the introduction and the discussion are very similar.

- Voronoi tessellation is used as structural descriptor for the two tree methods. Descriptor design is a very active research field and many highly optimised atomic descriptors are freely available in the literature. With a different descriptor, the RMSE would reduce and a predefined accuracy target could therefore be reached with less data. This would effectively achieve the same as the pruning method proposed here.

- The concept of data redundancy is central to the article, but no clear definition or independent way of quantifying redundancy is given. In fact, through observation of data saving in the learning curves, redundancy is inferred. It would be good to quantify data redundancy as seen through the applied materials descriptor and then correlate this measure of redundancy with the amount of data saving.

- The paragraphs describing Fig 2 (and also later other figures) from lines 175 to 214 do not describe the figure well. The paragraphs are full of numbers that are not so interesting, but do not discuss the function shape or the difference between the different lines or datasets presented in the figure.

- One interesting aspect of Fig 2 that is not mentioned in the discussion is that the neural network does much better for the formation energy than the band gap. Why is this the case?

- Fig 2 also shows that the performance for the two tree methods saturates for the formation energy of the OQMD14 dataset, but not for JARVIS18 and MP18. Why is that?

- Line 208 to 210: “the 10% RSME increase in this case actually corresponds to an RSME increase of 6 meV/atom”. It is hard to ascertain for me where this number comes from. Looking at panels a to c of Figure 2, I see that the RMSE reaches values around 0.06 eV for all three datasets. But that would be 60 meV and not 6 meV.

- Lines 225 to 231: “We find that this is achieved by skewing the data distribution towards the underrepresented materials. For instance, the distributions of the pruned data are skewed towards materials with large formation energies and band gaps, which are both underrepresented and less accurately predicted materials (Supplementary Figure 15 and 16).” Such insight would enrich the article and it would be good to have it in the main article.

- The performance quantification for the unused data does not add anything to the manuscript. How is this different to checking for the in-distribution error? And how do you take into account that the amount of unused data shrinks with increasing training set size?

- The out-of-distribution results are interesting, but the analysis is again poor. It would be interesting to quantify the statistical overlap between the original and the extended dataset for each dataset.

- Lines 295 to 299: “Therefore, the excellent ID performance obtained with state-of-the-art models and large datasets might be an overly optimistic estimation of the true generalization performance in a realistic materials discovery setting [33, 37].” This is an example of the iteration of a generally accepted fact. I think nobody would have expected the ID performance to be very transferable for these datasets. What would be interesting would be to find ways to quantify the expected generalization error (for new data).

- Lines 300 to 302: “The strong OOD performance degradation is essentially related to the information richness rather than the volume of the training data.” This is also not too surprising. If I train on dataset A and predict on dataset B, which has no overlap with A, then my prediction will not be particularly good, no matter how large dataset A is. What we would need are methods that determine efficiently what data in B needs to be labelled (if it isn’t already labelled) to generalise the model trained on A.

- Lines 325 to 365: These two paragraphs exclusively refer to figures and material in the supplementary information. I do not find this helpful. When I read an article, I typically do not have the SI at hand and can therefore not look at those figures. If the information in those figures is important, then it should be included in the main article.

- Uncertainty-based active learning: The acquisition function in the active learning loop is only based on uncertainty. I would expect that many of the most uncertain systems are structurally very similar. Therefore, basing the selection criterion only on uncertainty would not lead to the most diverse training set. I am bit surprised then that the active learning reduces the RMSE so quickly.

- I find it a bit odd that the hyper parameters are only optimised for the smallest training sets and then kept fixed. This would imply that the learning for larger training sets is suboptimal and could explain why pruning and active learning have such large effects. I would suspect that this is partly an artefact of the lack of hyper parameter optimization. Larger training sets learn less well than they could, which gives the random curve a smaller slope than if the hyper parameters were optimised for every training set size. This means that the intersect of the pruning and active learning curves is pushed to larger training set sizes resulting in seemingly larger data savings.

- The description of the pruning algorithm is very brief. More details please. For example, what is meant by “iteration” here? Also, by construction the pruning algorithm seems to require a model that is trained on all data first. So where is the saving?

Reviewer #3 (Remarks to the Author):

It was a pleasure to read and review the manuscript. Written in well-versed English, it raises an interesting and important question on how much data redundancy is there in the available large comp. material science datasets. The question is important because using redundant data waste computational resources (and heat up our planet on the way ;); more frugal data sets obtained by data pruning will also open research possibilities for smaller research groups that do not have huge computational resources to use a "brute force" approach in mat. sci. computations. The sets of non-redundant data are well known in other data intensive sciences (viz. e.g. < 90% sequence identity sets PDB subsets), so it is high time for material sciences to make similar inventory. The authors offer a working data pruning method to do so.

The authors convincingly demonstrate that indeed large amounts of data can be discarded from existing data sets without impairing efficiency of machine learning processes. The demonstration is done using both in-distribution (ID) and out-of-dist (OOD) data, which is a good control. It is an interesting and not self-evident outcome (at least to me) that reduced data sets are transferable

among ML architectures, but not so for different material properties, which opens intriguing questions for future research as to what information faced are important for specific material properties.

The description of the method kindled the idea in my head that information theoretical (Shannon entropy) measures could be used to quantify real information contents in large data sets, and I was pleased to see in the discussion that the authors mention similar ideas and cite Henning 2012 and Zhang 2023 on this matter.

In summary, this is interesting and important paper, with conclusions well supported by the evidence, and presenting ideas important not only for materials research but for all data-driven sciences in general. The data sets that were used for research are well documented and so the similar results could be obtained, following the ideas of the paper, independently. The methodology is sound, and I trust that authors will deposit their data and code as the paper is accepted, which will be of great use for those who wold like to apply similar methods to other fields (w.g. bioinformatics and QSAR in chemiformatics could also benefit from such analysis, IMHO).

In my view, the paper does not require any revision and I would love to see it published as presented, just after removing the second "the" on the line 249.

Point-by-Point Response to the Reviewers' Comments

We would like to thank the editors and reviewers for their careful review of our work and their comments, which are valuable and contribute to further improvement of our work. In what follows, we make point-to-point responses (marked in blue) to all reviewers' comments. The changes in the revised manuscript are marked in red, and the original text in grey. We hope our efforts address all the concerns, and the current version satisfies the criteria of *Nature Communications*.

Reviewer 1 (Remarks to the Author):

Li et al. present a timely and highly relevant contribution for the ML for Materials Science community and put a great highlight on the enhancement of sampling efficiency of active learning. In fact I discussed something very similar in my machine learning lecture this week on the QM9 dataset and kernel regression methods. The paper is overall very well written and presented concisely in such a way that I have all but one minor comment relating to the literature cited and putting this into context: Xi et al. present in 10.1038/s41586-019-1540-5 the issue relating to "Anthropogenic biases in chemical reaction data" and how it "hinders exploratory inorganic synthesis" and this paper is an excellent contribution from the field of organic chemistry. Then the paper by Wang et al. is IMHO not the (only) one to be cited here as both 10.1038/s41524-021-00656-9 and 10.1039/C9SC05999G predate it and demonstrate across multiple materials science domains. There is even a GitHub repository to measure both the enhancement factor and acceleration factor - two measures that express how much active learning "accelerates".

Response: We thank the reviewer for the positive evaluation of our work and for the suggested references on active learning, which have been now cited as Ref [29,32,33].

Reviewer 2 (Remarks to the Author):

Comment #1: Data redundancy in machine learning (ML) in materials science is investigated. This is mostly done in the context of ML model generation and to a lesser extent for saving time and cost in data labelling. For the study, 3 different publicly available datasets in Comput. Mater. science (JARVIS, MP, and OQMD) were used. These were partly chosen, because recent updates extend earlier versions of the dataset and make it possible to quantify out-of-distribution errors. Different ML models are trained and then reduced in size with pruning or active learning. By comparing to the largest ML models, the authors find that low in-distribution errors can

already be achieved with much smaller datasets pointing towards redundancy in the training set. The out-of-distribution error, however, remains high and suggests that the extensions to the datasets have reduced overlap with the original data.

Response #1: We thank the reviewer for the careful reviewing and the comments.

Regarding the comment "*This is mostly done in the context of ML model generation",* it should be emphasized that our redundancy analysis is done not only in the context of more efficient ML training; it is also very much motivated by efficient data labeling. As noted in the introduction, exposing redundancy in existing datasets can reveal the efficiency of the current practices in data labeling. We also have a dedicated section to demonstrate how active learning algorithms can enable more efficient data acquisition to construct compact datasets.

Regarding the comment *"These were partly chosen..."*, we would like to clarify that redundancy analysis is also performed on the updated databases, for which the performance on the ID test set and the unused data is evaluated and presented in the supplementary information.

Comment #2: The idea of applying a redundancy analysis to these nested datasets is very good and the out-of-distribution results are really insightful. Apart from that, the study does not offer much that is new. Many statements made in the manuscript are obvious. Challenging the "bigger is better" philosophy is of course appealing for a Nature-type article, but one should bear in mind that many datasets in materials science (the ones used by the authors included) are not primarily generated for training regression models. Secondly, if maximally compact datasets are generated with active learning, as the authors advocate, one has to be wary of the reusability. Due to the nature of their assembly, active learning datasets often exhibit a bias towards the target properties they were generated with and might not generalise for other purposes. This discussion is completely absent from the manuscript.

Response #2: We thank the reviewer for the appreciation for the redundancy analysis and for the out-of-distribution (OOD) results. In addition to the novelty of redundancy analysis and OOD results, this work also contains other new insights: For instance, we demonstrate that the optimal training set does not have to be of the same data distribution as the in-distribution test set; we also show that our active learning algorithms can lead to a rapid decrease in RMSE. These are two examples of unexpected results for the reviewer in Comment #4 and #18, respectively. Comments from reviewer #3 also show that our results of transferability are interesting and novel.

While these datasets were not originally generated for training ML models, as noted by the reviewer, this does not reduce the interests and impacts of our work: First, irrespective of the original purpose of database developers, the presence and degree of redundancy of a dataset

is objective and worth discussing. Second, model training is the *de facto* primary use case of these datasets, irrespective of their original purpose. Third, ML should be considered not only as a purpose, but also as a statistical or data-science tool to quantify information richness. As noted in Comment #1, this work is not just to quantify redundancy for better ML training, but also to use ML as a tool to quantify redundancy and to inform efficient data labeling. On the other hand, this work is by no means a criticism of the efforts of database developers – their data were obtained at the very early stage of the application of ML in materials science, and it is their efforts in open data sharing and curation that have enabled fast application of ML in materials science. Instead, this work is intended to be a reflection on the current practices to better inform future database development. We have made accordingly the following clarifications in the manuscript:

(Lines 471-484) It is worth emphasizing that this work is by no means critical of the curation efforts or significance of these materials datasets. Indeed, many datasets were not originally generated for training ML models; they are the results of long-term project-driven computational campaigns. Some of them were even curated before the widespread use of ML and have played a significant role in fueling the rapid adoption of ML in materials science. On the other hand, the presence and degree of redundancy in a dataset is worth discussing irrespective of the original purpose. Furthermore, ML should be considered not only as a purpose, though it has become a primary use case of these datasets, but also as a statistical means of examining and improving these datasets.

Reusability is referred to as task transferability and discussed in Section II C of the manuscript, where we show that there is limited transferability across tasks (properties). We have also added the following paragraph in the discussion section:

(Lines 530-555) We demonstrate that transferability of compact datasets is reasonable across models but is limited across tasks (materials properties). It is discussed in the context of data pruning, but the idea and implication hold for active learning. The limited task transferability indicates that the maximally compact set of materials for property A is not ensured to be the maximally compact set for property B. While this is an interesting observation and invites further investigation, it is not a practical issue for active learning when the measurements of two properties are independent. For example, DFT calculations of band gap and elastic modulus are unrelated, therefore the maximally compact sets of materials can be constructed independently via active learning and need not be the same. For correlated property measurements, however, more careful planning is required. For instance, the calculations of more "expensive" properties such as band gap and elastic modulus would also give the formation energy of the same material since energy is a basic output of any DFT calculations. While the compact datasets for band gap and elastic modulus can still be searched independently without considering formation energy data, the construction of the compact dataset for formation energy should consider the data that can be obtained as by-products from the band gap and elastic modulus calculations.

Comment #3: Furthermore, I am doubtful that the active learning results presented in Figure 5 are correct. In the Methods section, the authors state “During the active learning process, the training set is initially constructed by randomly sampling 1% to 2% of the pool […]”. This would mean that for the smallest training set size of 1 or 2% of the full training set, random sampling and active learning should agree with some statistical fluctuations. Unfortunately, this low range of the training set size is cut off in Figure 5. But it would appear as though the RMSE of the formation energy (panel a) is actually higher in active learning than in random sampling and for the band gap it might be lower. I strongly encourage the authors to check their calculations and to show these consistency checks in the paper or at least the supplementary information. I would also strongly recommend to average the RMSE over several runs to remove statistical fluctuations from the learning curves.

Response #3: We thank the reviewer for pointing out this potential confusion. The reviewer’s concern arises because of the absence of the data range below 5% in Fig 5 (attached below).

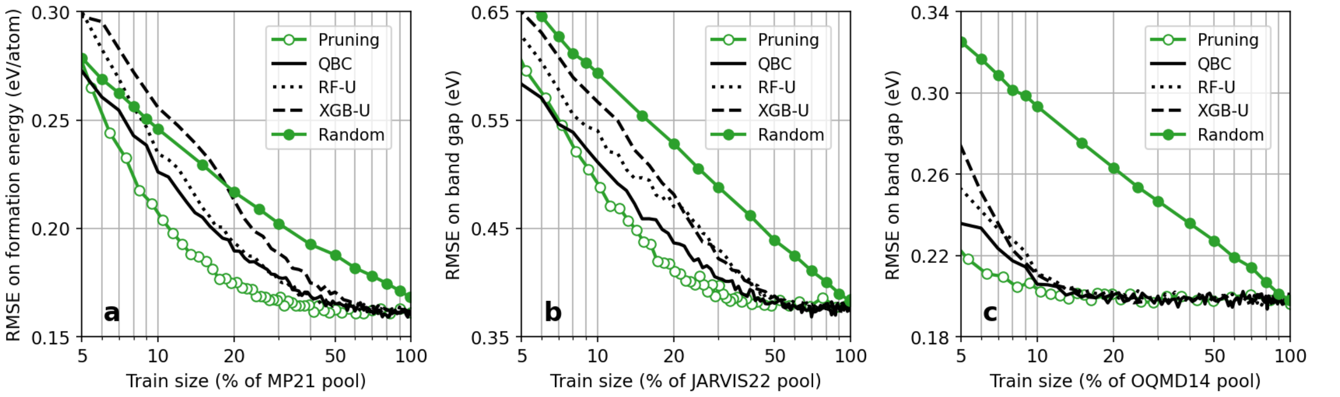

Original version of Figure 5 in the main text

Below we show the figure version with the data range starting from 1%. As expected, the active learning curves are close to the random-sampling curve at the initial training set size (1% for QBC and RF-U, and 2% for XGB-U), therefore addressing the concern over consistency.

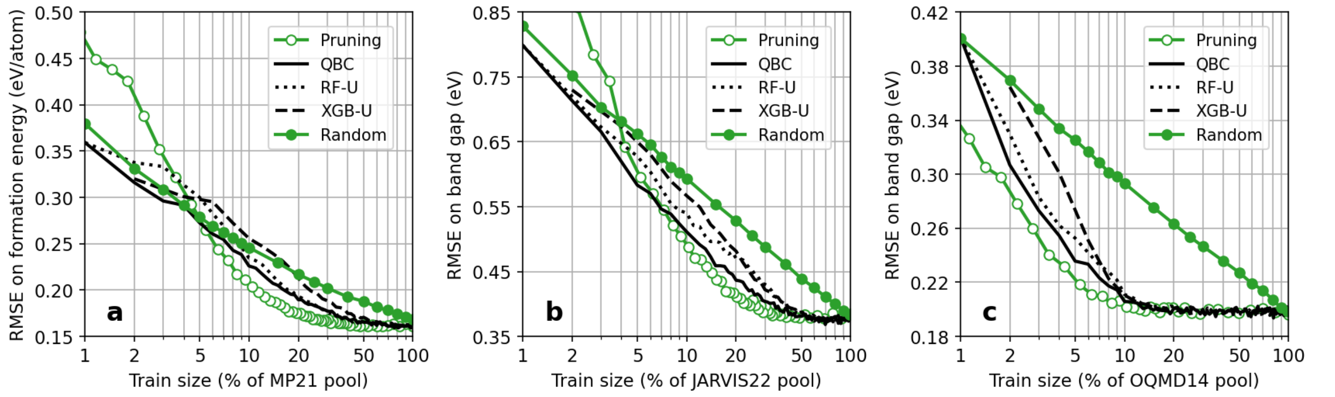

The data range below 5% was not shown previously because this data range is irrelevant to the redundancy issue discussed in the manuscript. Based on the same consideration, we still keep the scale in Fig 5 unchanged; but we have updated all the active learning curves in the supplementary figures (attached below) to include the 1% data range:

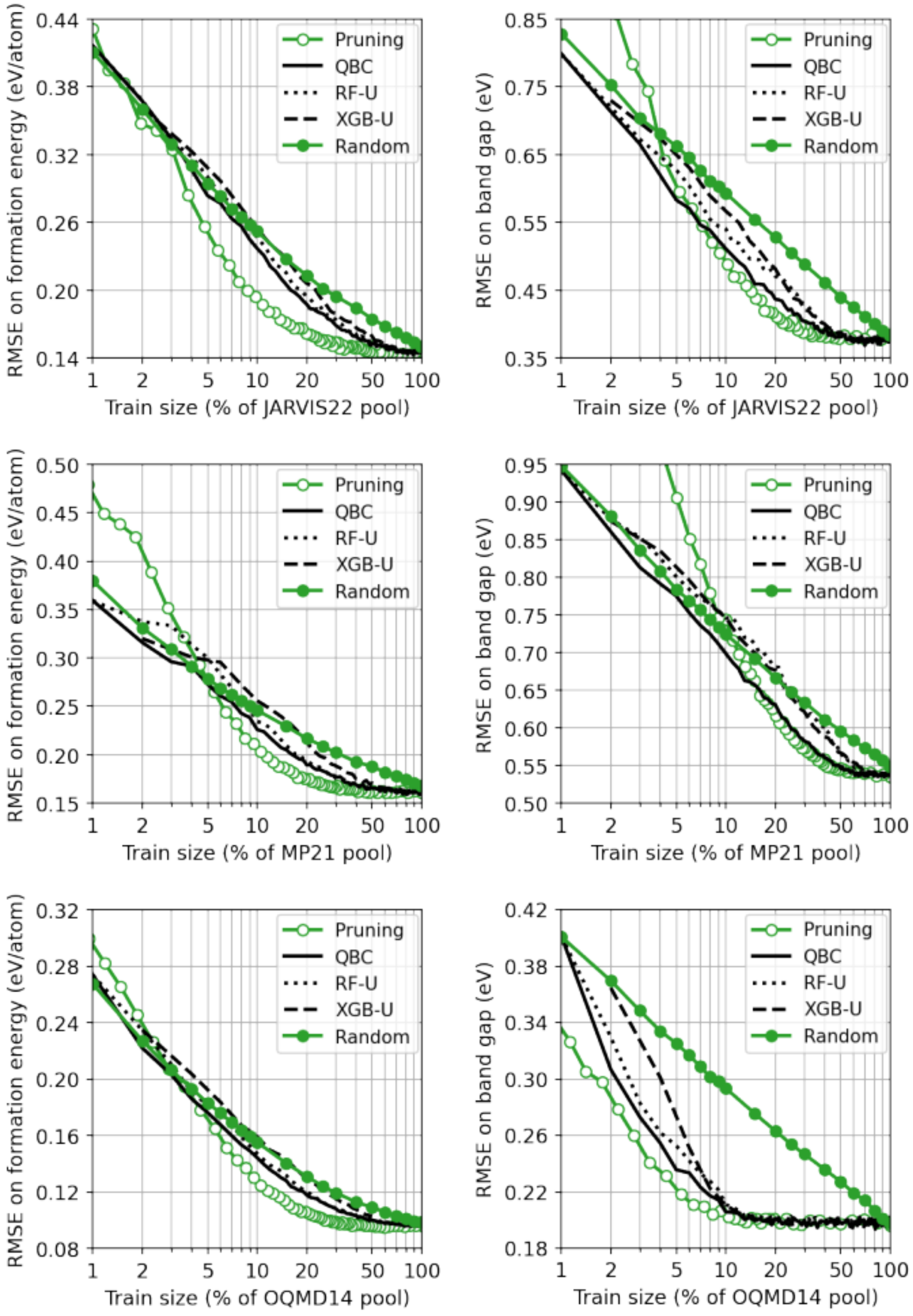

Supplementary Figure 22. ID performance of the XGB models for JARVIS22, MP21, and OQMD14 formation energy and band gap datasets, using the uncertainty-based active learning algorithms. For

each dataset, we show the RMSE scores obtained using the randomly selected data, the XGB-pruned data, and the data selected using the uncertainty-based active learning algorithms.

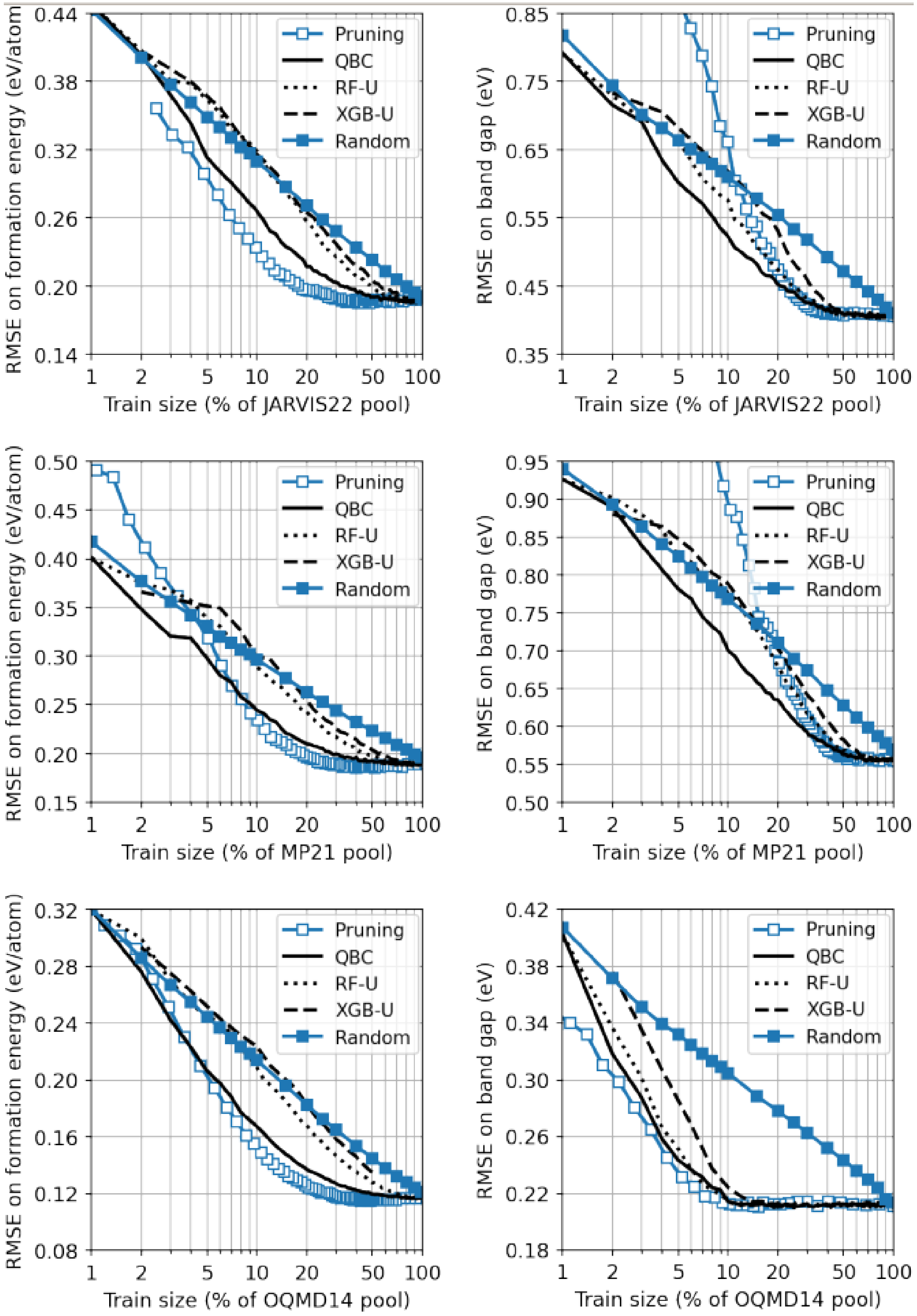

Supplementary Figure 23. ID performance of the RF models for JARVIS22, MP21, and OQMD14 formation energy and band gap datasets, using the uncertainty-based active learning algorithms. For each dataset, we show the RMSE scores obtained using the randomly selected data, the RF-pruned data, and the data selected using the uncertainty-based active learning algorithms.

We understand the reviewer's concern over the statistical fluctuations. We have followed the reviewer's recommendation by performing additional runs to average the RMSE as shown below:

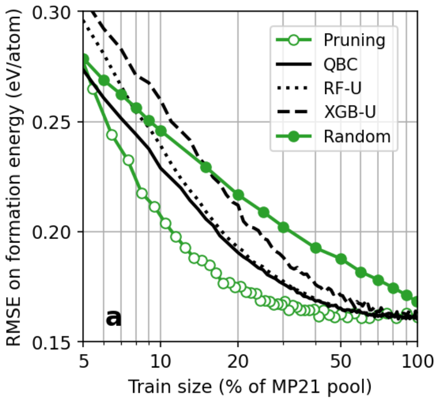

Revised version of Figure 5 in the main text

Here the QBC and RF-U results were averaged over five runs, while the XGB-U results were averaged over two runs (because of the high compute cost). For convenience of comparison, the original Fig 5 is attached below. The comparison shows that the active learning results are largely the same except for some localized fluctuations that have no impact on our conclusion.

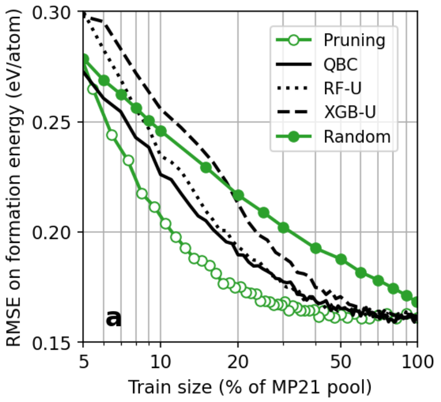

Original version of Figure 5 in the main text

Previously we did not average over multiple runs because of the high compute cost (in particular for the XGB-U algorithm, which took more than 3 weeks for a single run); doing so for all datasets would be very expensive. Furthermore, because of the large volume of these datasets, the statistical fluctuations are expected to be small and would not impact the conclusions, which is also common in computer science studies where a single train-test split is used rather than a 5-fold cross-validation (provided the dataset is large enough).

Comment #4: I am furthermore surprised that the authors achieve large data savings with their active learning models. The in-distribution test set is drawn randomly from the whole dataset.

The test set distribution and the training set distribution should therefore be very similar and random sampling should be the optimal strategy to minimise a global quantity like the RMSE.

Response #4: The reviewer assumes that the distribution of the optimal training has to be the same as the test set and hence questions the possibility of large data savings. However, very recent studies on image recognition (see, e.g., Ref [23] from ICLR 2023 and Ref [24] from NeurIPS 2023) have confirmed, with both theoretical backing and empirical evidence, that high-quality pruning can lead to better model performance than random sampling and can achieve data saving.

We also try to understand the possible rationale behind the reviewer's assumption, which might be as follows: Suppose that the data set consists of a large percentage (p_A) of materials of type A, and a small percentage (p_B) of materials of type B. The in-distribution test error is then equal to $p_A e_A + p_B e_B$, with e_A and e_B the errors associated with the two types of materials. As the errors for type-A materials have a larger weight in the test RMSE, reducing the errors in type-A materials is more important, and our training set should contain a large portion of type-A materials.

However, this rationale is not valid because it only considers the distributions (p_A and p_B) but neglects the marginal change in errors (e_A and e_B). It is possible that type-A materials are already well described with respect to type-B materials, so using a smaller portion of type-A materials in the training set only results in a slightly larger e_A but a much smaller e_B such that the overall RMSE is reduced. In this way, large data saving can be "achieved by skewing the data distribution towards the underrepresented materials" (see the reviewer's comment #12).

Please note that this "over-representation" issue can result from the inherent physics behind and/or the dataset bias. Below we provide two toy examples to illustrate this point. The figure on the left represents the case where our dataset is unbiased but there is a region with a flat response surface. As the underlying relationship in the flat region is inherently simple, we can reduce the training data in that region with little loss in prediction accuracy. The figure on the right represents the case where our dataset is biased towards the region around the second peak. Even though the underlying relationship is not necessarily simple, this region is still considered to be over sampled with respect to the complexity of the underlying relationship.

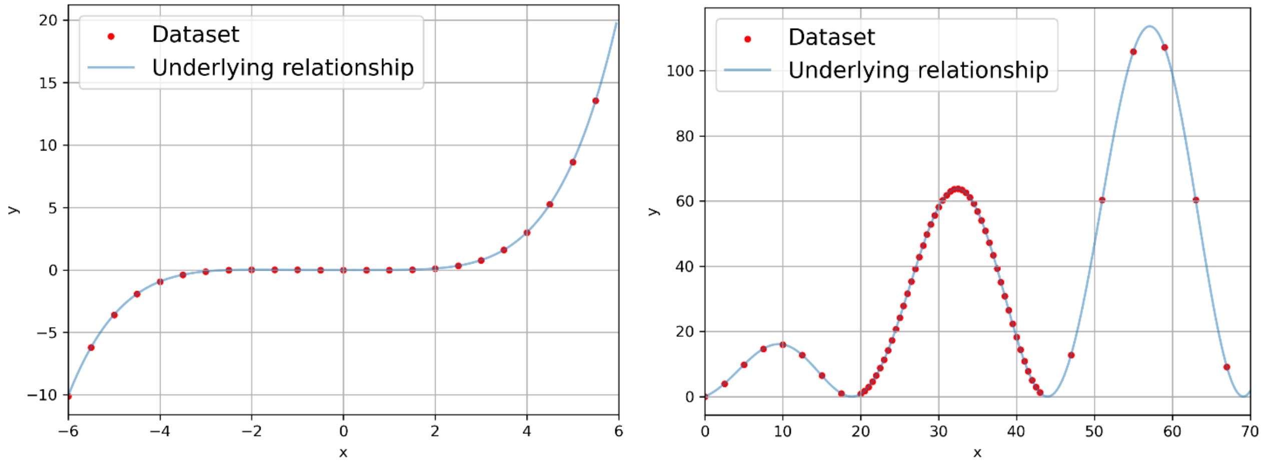

Comment #5: Considering these aspects, I cannot recommend the manuscript for publication in Nature Communication. More detailed comments are given below:

- Overall the article is a bit repetitive as the introduction and the discussion are very similar.

Response #5: We have simplified the discussion to be more focused on the implications.

(Now removed from discussion) ~~*Data redundancy evaluation of existing datasets may also inform and motivate the community to create smaller benchmark datasets by eliminating uninformative data, thereby significantly reducing training costs and facilitating machine learning model development and selection. This may be important in the future if data volume grows much faster than the available training budget, which is a likely scenario, as data volume is proportional to resources available to the entire community, while training budgets are confined to individual research groups.*~~

(Introduction. Lines 69-74) Revealing data redundancy can inform and motivate the community to create smaller benchmark datasets, hence significantly scaling down the training costs and facilitating model development and selection. This may be important in the future if data volume grows much faster than the available training budget, which is a likely scenario, as data volume is proportional to resources available to the entire community, while training budgets are confined to individual research groups.

(Discussion. Lines 511-529) While the pruning algorithm is proposed here to illustrate data redundancy, such data selection algorithms can have other use cases, e.g., inform the design of active learning algorithms. Indeed, the observation that data redundancy predominantly involves overrepresented materials implies that information entropy might also serve as a promising criterion for data acquisition [39,44]. A detailed analysis of pruned material sets may also offer insights into material prototypes and improve understanding of feature-property relationships, including identifying specific groups of redundant materials as well as identifying patterns that explain the poor task transferability of pruned datasets. Finally, the pruning algorithm offers a new funneling strategy for prioritizing materials for high-fidelity measurements. For instance, pruning the existing DFT data obtained with generalized gradient

approximation (GGA) functionals can point to the materials to be recomputed with high-fidelity meta-GGA functionals [34].

Comment #6: Voronoi tessellation is used as structural descriptor for the two tree methods. Descriptor design is a very active research field and many highly optimised atomic descriptors are freely available in the literature. With a different descriptor, the RMSE would reduce and a predefined accuracy target could therefore be reached with less data. This would effectively achieve the same as the pruning method proposed here.

Response #6: As mentioned by the reviewer in Comment #1 ("by comparing to the largest ML models, the authors find that low in-distribution errors can already be achieved with much smaller datasets pointing towards redundancy in the training set"), the comparison is made on a per-method basis: the accuracy target is defined, on a relative scale for each ML method, as the accuracy obtained using the full training pool. Namely, the redundancy is quantified as seen through a given ML method. This is why we always specify the method under consideration when discussing redundancy in the manuscript. Please note that here the term "ML method" refers to the descriptor set + ML architecture combination.

Comment #7: The concept of data redundancy is central to the article, but no clear definition or independent way of quantifying redundancy is given. In fact, through observation of data saving in the learning curves, redundancy is inferred. It would be good to quantify data redundancy as seen through the applied materials descriptor and then correlate this measure of redundancy with the amount of data saving.

Response #7: In the original manuscript, we defined a quantitative threshold to define redundancy:

(Lines 199-205) A large portion of training data can be removed without significantly hurting the model performance. To demonstrate this, we define a quantitative threshold for the "significance" of the performance degradation as a 10% relative increase in RMSE; data that can be pruned without exceeding this performance degradation threshold are considered redundant.

In our definition, redundancy is equivalent to data saving: it is the amount of data that can be saved without compromising the model performance. As noted in the previous comment, we measure redundancy of a dataset on the per-method basis.

The reviewer's distinction between data saving and redundancy might be that the reviewer considers redundancy to be an inherent method-independent property of dataset, whereas data

saving is method dependent. However, we would argue that such a method-independent definition of redundancy is impractical because measuring redundancy necessarily involves adopting certain methods.

In particular, we consider input representation (e.g., descriptor set) to be a part of the model specification and method. Hence, measuring redundancy with descriptors, as advocated by the reviewer, would still be subject to the choice of the descriptors and hence the choice of the ML method. Furthermore, using only descriptors does not appear to be a robust way to quantify redundancy. For instance, our results in Fig 2 suggest that the level of redundancy is different between the formation energy and band gap data, even if the same set of descriptors is used. This indicates that redundancy is also correlated with the underlying descriptor-property relationship (e.g., whether the relationship is complex or easy to learn, as also illustrated in Response #4).

Comment #8: The paragraphs describing Fig 2 (and also later other figures) from lines 175 to 214 do not describe the figure well. The paragraphs are full of numbers that are not so interesting, but do not discuss the function shape or the difference between the different lines or datasets presented in the figure.

Response #8: These paragraphs are focused on quantitative description of redundancy on a per-method and per-dataset basis. The function shape (scaling law) and comparison across different lines (ML methods) and subplots (datasets) are not central to the discussion of redundancy, but they may be of interests to some readers and have been added as shown below:

(In-distribution performance. Lines 220-238) While this work is focused on redundancy which is model and dataset specific, it is still worth commenting on the model performance scaling across models and datasets. When using the random sampling for data selection, we observe a power law scaling for all the models and datasets. For formation energy datasets, switching the models mainly shifts the scaling curve without much change to the slopes. For band gap datasets, switching from RF to XGB models shifts the scaling curve down without changing the slope, whereas switching from tree-based models to ALIGNN leads to a steeper slope and hence better scaling. Compared to training on randomly sampled data, training on informative data as selected by the pruning algorithm can lead to better scaling until reaching saturation when there is no more informative data in the pool. Different datasets exhibit similar scaling behaviors with the slope and saturation point dependent on target property and material space covered by the datasets.

(Performance on unused data. Lines 293-305) While the scaling curve for the unused data has a shape similar to the one for the ID test data, the former shows a much steeper slope for the training set sizes below 15% of the pool, and reaches saturation at a slower rate. In addition, it is noted that the ranking of different ML models for their performance on the unused data is not necessarily the same as for the ID

test data. For instance, for the JARVIS18 and MP18 formation energy data, the XGB model outperforms the RF model on the ID test set whereas their performance is practically the same on the unused data. Among the models trained on the OQMD14 band gap data, the RF model has the largest RMSE on the ID test set but the lowest error on the unused data.

(Out-of-distribution performance. Lines 318-321) … Overall, the scaling curves for the OOD performance show are similar to those for the ID performance with slightly different slopes and saturation data size, confirming the existence of the data redundancy measured by the OOD performance. Specifically…

(Out-of-distribution performance. Lines 338-349) Different databases exhibit a varying degree of performance degradation, which should be correlated with the degree of statistical overlaps between the database versions rather than the quality of the databases. In fact, database updates that induce such performance degradation are desirable because they are indications of new ``unknown" observations and can lead to more robust generalization performance. One interesting line of research would be therefore to develop methods to deliberately search for materials where the previous models would fail catastrophically as a path to expand a database.

Comment #9: One interesting aspect of Fig 2 that is not mentioned in the discussion is that the neural network does much better for the formation energy than the band gap. Why is this the case?

Response #9: Indeed, it has been observed in community benchmark results that neural networks tend to work much better for formation energy than for other properties including band gap (see, e.g., npj Comput. Mater. 6, 138 (2020), and arXiv:2306.11688 (2023)). A very recent investigation by Gong et al. (arXiv:2208.05039 (2023)) analyzed such limitations of the GNN models. To the best of our knowledge, however, the reason behind is not completely clear. It may be that current ML models typically express a property as a sum of local contributions (pair, or many-body terms). From a physical point of view, this is justified for energy but is less evident for properties such as band gap. Therefore, the ML representation may be more compatible with the underlying physics for energy than for other properties, making it possible to improve accuracy for energy prediction with the more advanced neural networks, whereas this is hard for other properties due to the inherent limitations behind the ML model/representation. Of course, the complete explanation may be multi-fold and more complicated.

This aspect is not discussed in the manuscript because it is not a novel observation, and the explanation is still an open question and worth future investigation. More importantly, such discussion is beyond the scope of this work as it is irrelevant to data redundancy.

Comment #10: Fig 2 also shows that the performance for the two tree methods saturates for the formation energy of the OQMD14 dataset, but not for JARVIS18 and MP18. Why is that?

Response #10: This might be a misreading of Fig 2 because they show saturation for the formation energy of all three datasets. As shown in the original Fig 2.(a)-(c) attached below, the pruning curves for the two tree methods (unfilled squares and circles) saturate around 30-40% of the JARVIS18 pool in Fig 2(a), around 40% of the MP18 pool in Fig 2(b), and around 30% of the OQMD14 pool in Fig 2(c).

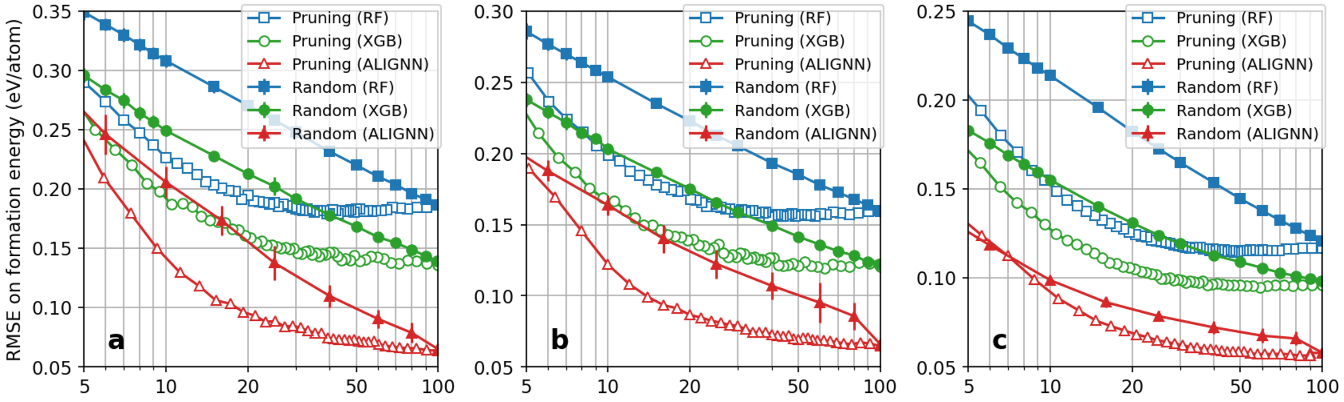

Figure 2 in the main text

Comment #11: Line 208 to 210: “the 10% RMSE increase in this case actually corresponds to an RMSE increase of 6 meV/atom”. It is hard to ascertain for me where this number comes from. Looking at panels a to c of Figure 2, I see that the RMSE reaches values around 0.06 eV for all three datasets. But that would be 60 meV and not 6 meV.

Response #11: The original sentence lacked clarity. As the reviewer noted, the best RMSE reaches 60 meV/atom, so 10% RMSE increase is an increase of 60*10% = 6 meV/atom in the RMSE. We have clarified the cited sentence in the revised manuscript as follows:

(Lines 213-215): the 10% increase in the RMSE (60 meV/atom) corresponds to an RMSE increase of only 6 meV/atom.

Comment #12: Lines 225 to 231: “We find that this is achieved by skewing the data distribution towards the underrepresented materials. For instance, the distributions of the pruned data are skewed towards materials with large formation energies and band gaps, which are both

underrepresented and less accurately predicted materials (Supplementary Figure 15 and 16)." Such insight would enrich the article and it would be good to have it in the main article.

Response #12: We have added a new figure (Fig. 3 in the revised manuscript) for the illustration. The main text has been revised as follows:

(Lines 251-253) … the distributions of the pruned data are skewed towards materials with large formation energies and band gaps (Fig 3) …

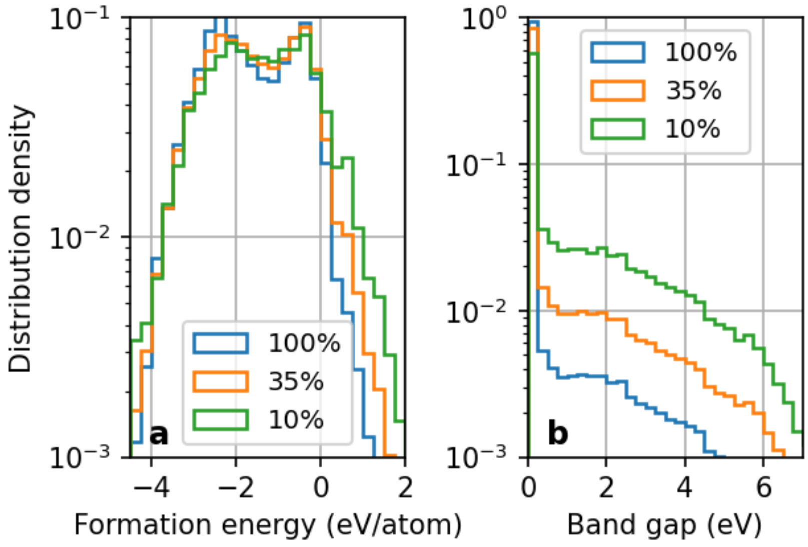

Fig. 3. Label distributions of the XGB-pruned training sets. ***a*** *MP18 formation energy data.* ***b*** *OQMD14 band gap data. The legend indicates the training set size in percentage of the pool. Results for other datasets can be found in Supplementary Figure 15 and 16.*

Comment #13: The performance quantification for the unused data does not add anything to the manuscript. How is this different to checking for the in-distribution error? And how do you take into account that the amount of unused data shrinks with increasing training set size?

Response #13: The motivation of the performance quantification for the unused data has been explained in the original manuscript (attached below):

(Lines 258-264) ID performance is not sufficient to prove that the unused data are truly redundant. The effects related to model capability and the test set distribution should also be considered. Indeed, one may argue that the current ML models (in particular, the band gap models) are not advanced enough to learn from the unused data leading to a false sense of the data redundancy.

The motivation here is to confirm that the in-distribution (ID) performance saturation is not an artifact of incapability of ML methods. Take, for example, a naïve model which always gives a prediction equal to the mean of the labels in the training set. Clearly, for any size of the randomly sampled subsets, the resulting ID performance would be the same (within statistical fluctuations). The ID performance saturation would therefore occur with very small training sets, but this is merely an artifact of incapability of the model, which can be revealed by showing that the performance on the unused data is the same as (or rather, as poor as) the ID performance.

The performance quantification for the unused data is particularly important for band gap since their ID test errors are relatively large and could raise similar concerns. It is therefore necessary to confirm with Fig. 4c that the unused data can be well predicted and truly redundant, as mentioned in the text:

(Lines 287-292) The good prediction on the unused data signifies a lack of new information in these data, confirming that the improvement saturation in the ID performance is caused by the information redundancy in the unused data rather than the incapability of models to learn new information.

The shrinking amount of unused data does not impact the robustness of our conclusion of performance saturation and data redundancy. The performance saturation occurs for training set sizes at 50% or a smaller portion of the pool and the unused data accounts for 50% or a larger portion of the pool. Therefore, there is enough unused data to compute statistically meaningful performance metrics.

Comment #14: The out-of-distribution results are interesting, but the analysis is again poor. It would be interesting to quantify the statistical overlap between the original and the extended dataset for each dataset.

Response #14: The main objective of the out-of-distribution (OOD) discussion is to demonstrate that the saturation in the in-distribution performance is also observed in the OOD performance. The severe OOD performance degradation is an interesting observation, but performing a statistical analysis of the cause is beyond the scope of this study focused on redundancy.

In fact, similar OOD degradation was already revealed and analyzed in our recent work in Ref [35] npj Comput. Mater. 9, 55 (2023), which provides a dedicated analysis for the formation energy prediction of metallic alloys in the MP dataset. An equivalent statistical analysis across multiple properties and datasets would require a significant amount of additional work and is not the focus of the present study. Nonetheless, we have now included a short analysis of this overlap in the supplementary material for interested readers.

(Supplementary Material)

Section V: STATISTICAL OVERLAP BETWEEN THE TRAINING POOL AND OOD DATA

In Fig. 4 of the main text, we demonstrate that there is a strong OOD performance degradation for the formation energy prediction for the JARVIS and MP datasets, and the band gap prediction for the OQMD dataset. To better understand the statistical overlap between the older dataset and the OOD data in the newer dataset, we provide below an analysis in the feature space using Uniform Manifold Approximation and Projection (UMAP), which is a stochastic and non-linear dimensionality reduction algorithm that preserves the data's local and global structure [2]. This technique was previously used to analyze a similar OOD performance degradation for the formation energy prediction of metallic alloys in the MP dataset in our recent work [3], and interested reader is referred to the work therein for an extended discussion.

Here we take the JARVIS database as an example to describe how the UMAP analysis is done. We first perform the standardization of Matminer-extracted features over the whole JARVIS22 dataset. Then we sequentially drop highly correlated features using a Pearson correlation threshold of 0.7. Next, the remaining features are used as inputs of UMAP to create the embeddings for the whole JARVIS22 dataset. We use n neighbors=300 and the default values for other UMAP hyperparameters. We visualize the data in the UMAP embedding with three subplots (see the 1st row in Supplementary Figure 24): the first subplot shows the JARVIS18 data on top of the new data in JARVIS22 (OOD data); the second subplot shows the JARVIS18 only; the third subplot show the OOD data, which are colored by the prediction errors of the XGBoost model trained on the whole JARVIS18 dataset. The results for the MP and OQMD datasets are obtained similarly (see the 2nd and 3rd rows in the 1st row in Supplementary Figure 24).

For the JARVIS database, the OOD data is almost covered by the training data in the UMAP-projected feature space. For the MP database, the OOD data also largely overlaps with the training data, with only two small clusters less well covered by the training data. For the OQMD database, there is a relatively large portion of OOD data lying beyond the region covered by the training data. The degree of the overlap might explain to some extent the degree of the OOD performance degradation: for instance, the overlap for the JARVIS database almost reaches 100 %, which may explain why its OOD performance degradation is the least significant among the three datasets (the Fig. 4 of the main text). However, the subplots showing the OOD data colored by their prediction errors indicate that the OOD data with large prediction errors also occur in the region well covered by the training data. This suggests that more in-depth analysis is needed in the future work to better understand the correlation between the overlap in the feature space and the prediction errors.

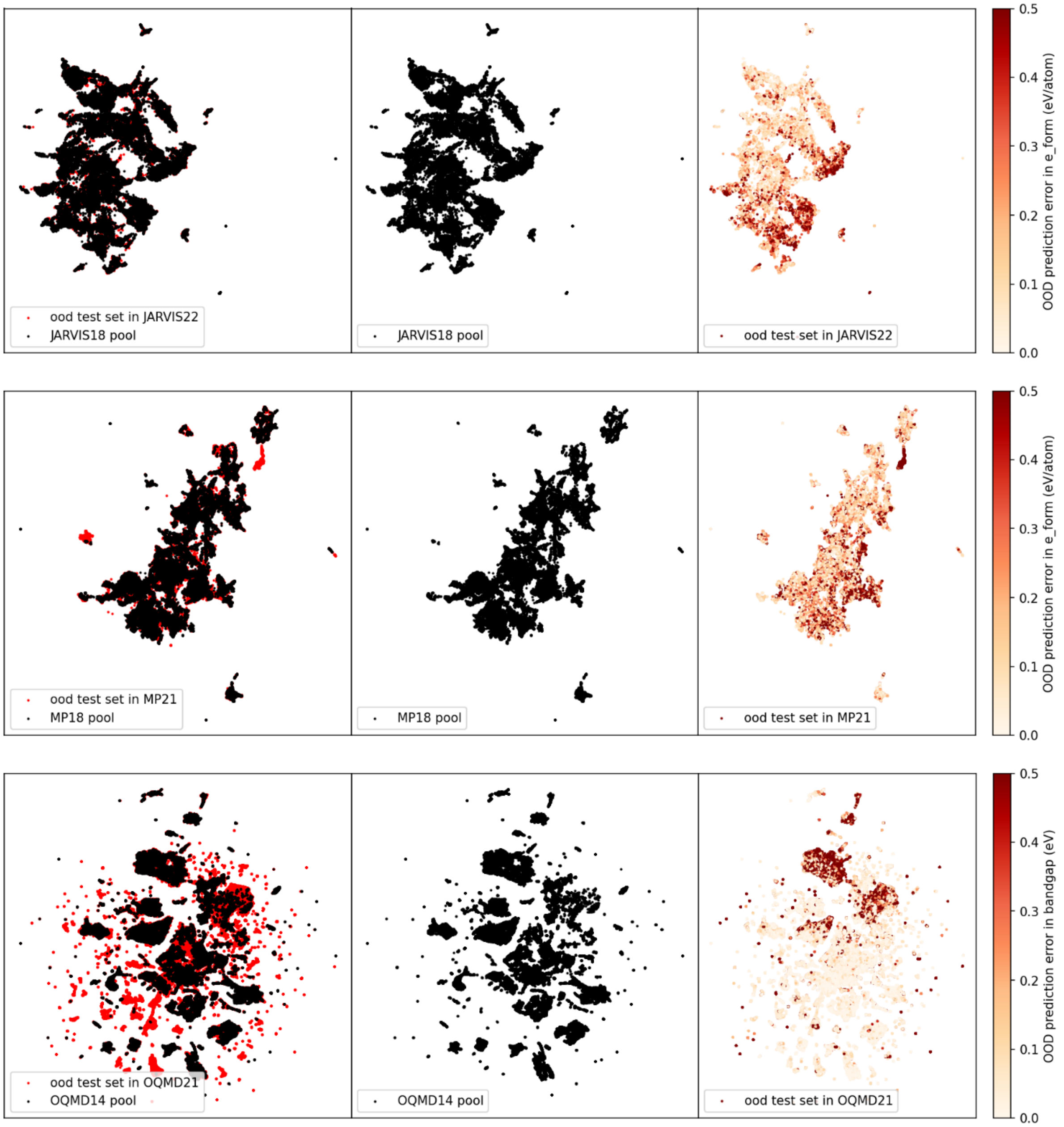

Supplementary Figure 24. UMAP projection in the feature space.

We have also added the following sentence in the main text to point to the relevant supplementary figure.

(Caption of Fig. 4 in the main text): …… The reader interested in the statistical overlaps between the ID and OOD data in the feature space is referred to Supplementary Figure 24.

Comment #15: Lines 295 to 299: "Therefore, the excellent ID performance obtained with state-of-the-art models and large datasets might be an overly optimistic estimation of the true generalization performance in a realistic materials discovery setting [33, 37]." This is an example of the iteration of a generally accepted fact. I think nobody would have expected the ID performance to be very transferable for these datasets. What would be interesting would be to find ways to quantify the expected generalization error (for new data).

Response #15: This is a misunderstanding of the emphasis. Here we are not restating that the transferability between the ID and OOD performance would not be very good. Instead, we are emphasizing that in some cases the ID performance is a *catastrophically* optimistic estimation and its transferability outright fails. Indeed, many of the researchers in the field would not expect such a severe performance degradation for their state-of-the-art models against database update. In addition, our results are a concrete demonstration that, for high throughput materials databases, potential datasets with little overlap with the training set are unintuitively easy to find in the course of routine mission-driven research. We have revised the sentence to clarify the emphasis:

(Lines 333-337) Therefore, the excellent ID performance obtained with state-of-the-art models and large datasets might be a catastrophically optimistic estimation of the true generalization performance in a realistic materials discovery setting.

We agree with the reviewer on the importance of uncertainty quantification for new data. We have some closely related discussion in our recent work in Ref [35] npj Comput. Mater. 9, 55 (2023). However, developing robust uncertainty quantification techniques is beyond the scope of the present redundancy study but will be a priority of our future work.

Comment #16: Lines 300 to 302: "The strong OOD performance degradation is essentially related to the information richness rather than the volume of the training data." This is also not too surprising. If I train on dataset A and predict on dataset B, which has no overlap with A, then my prediction will not be particularly good, no matter how large dataset A is. What we would need are methods that determine efficiently what data in B needs to be labelled (if it isn't already labelled) to generalise the model trained on A.

Response #16: We agree with the reviewer's example, but this example does not accurately capture the emphasis of the cited sentence and the paragraph. The reviewer's example is focused on the comparison between the training set A and test set B, whereas our emphasis is on the comparison of the information richness between the two training sets A1 and A2.

Thanks to the reviewer's comment, we have now the following clarification of our emphasis:

(Lines 350-358) The strong OOD performance degradation highlights the importance of information richness over data volume. It also raises an interesting question: given a training set A1, is it possible to find a smaller training set A2 such that the A2-trained model perform similarly to the A1-trained model on an A1-favorable test set B1 (i.e., same distribution as A1) but significantly outperform the A1-trained model on an A1-unfavorable test set B (i.e., distribution different from A1)? Indeed, we find that training on the heavily pruned MP21 pool (A2) gives dramatically better prediction performance on the MP21 test data (B*) than training on 10x more data from the MP18 pool (A1) whereas their performance is similar on the MP18 test set (B1). The result confirms the idea of finding a training set whose distribution can not only well cover but also significantly extend beyond the original one while still being much smaller in size. The result highlights that information richness and data volume are not necessarily correlated, and the former is much more important for the prediction robustness. By covering more materials within the data distribution, we may better ensure unknown materials are from known distributions (``known unknown'') and avoid unexpected performance degradation (``unknown unknown''), which is particularly important in scenarios such as materials discovery or building universal interatomic potentials [22,42,43].*

We agree with the reviewer on the importance of developing methods for OOD identification and for active learning. Our recent work in Ref [35] npj Comput. Mater. 9, 55 (2023) addresses these issues for a case study of metal alloys in Materials Project, showing that the OOD performance degradation can be efficiently solved by adding only 1% of the new data, using the QBC as a way for OOD identification. In addition, in the present study we also demonstrate that our active learning methods are very effective in identifying the next data to label.

Comment #17: Lines 325 to 365: These two paragraphs exclusively refer to figures and material in the supplementary information. I do not find this helpful. When I read an article, I typically do not have the SI at hand and can therefore not look at those figures. If the information in those figures is important, then it should be included in the main article.

Response #17: The supplemental information is not included here because it is not essential and could be distracting. For convenience of discussion, we attach below the two paragraphs and underline the sentences that refer to the supplementary information:

(Lines 384-392) We find a reasonable level of transferability of the pruned material set across ML architectures, confirming that data pruned by a given ML architecture remains informative to other ones (<u>Supplementary Figure 17-20). For example, XGB models trained on RF-pruned data outperform those trained on twice as much randomly selected data for formation energy prediction. Moreover, the XGB model still outperforms an RF model trained on the same pruned data</u>...

(Lines 403-408) In contrast, we find that there is a limited transferability of pruned datasets across different material properties. <u>For instance, the band gap models trained on the pruned formation energy</u>

data outperform those trained on randomly sampled data by only by a slight margin (Supplementary Figure 21) ...

We think the underlined sentences already provide clear and concise summaries of the relevant supplementary information. The supplementary information is provided as supporting evidence but not essential for the understanding of the underlined sentences. Furthermore, the rest of the paragraphs are only focused on the implications rather than on the descriptions of the supplementary information. In addition, including such extensive information could be overwhelming and distracting, especially considering that there is already a large amount of information presented in the main text.

Comment #18: Uncertainty-based active learning: The acquisition function in the active learning loop is only based on uncertainty. I would expect that many of the most uncertain systems are structurally very similar. Therefore, basing the selection criterion only on uncertainty would not lead to the most diverse training set. I am bit surprised then that the active learning reduces the RMSE so quickly.

Response #18: We are unaware of the rationale or evidence behind the reviewer's statement. In fact, a similar query-by-committee acquisition policy was already proven to be effective in Ref [35] npj Comput. Mater. 9, 55 (2023) showing that the OOD performance degradation can be efficiently solved by adding only 1% of the new data. The code to reproduce that work can be found on GitHub at https://github.com/mathsphy/paper-ml-robustness-material-property.

For this work, we perform additional analysis to demonstrate that our active learning method leads to a diverse sampling of data. The analysis for the QBC active learning for the OQMD14 band gap data is shown in the figure below, which is the UMAP projection in the feature space for the new training data added at different iterations. In total we perform 100 active learning iterations and in each iteration 1% of the pool data is added to the training set. The first subplot (iteration 0) is the (initial) randomly sampled training data, whereas the other subplots are the newly added training data in each iteration. The data are colored by the true band gap values. Two things should be noted: First, the data added in each iteration is spread over a wide feature space, confirming the diversity of the data. Second, the data added in the first few iterations are relatively concentrated, but they also skew towards materials with large band gaps, hence enabling a better learning of the feature-property relationship. This explains why the corresponding QBC curve quickly reaches the performance saturation at only 10% of the pool size (Fig. 5c of the main text).

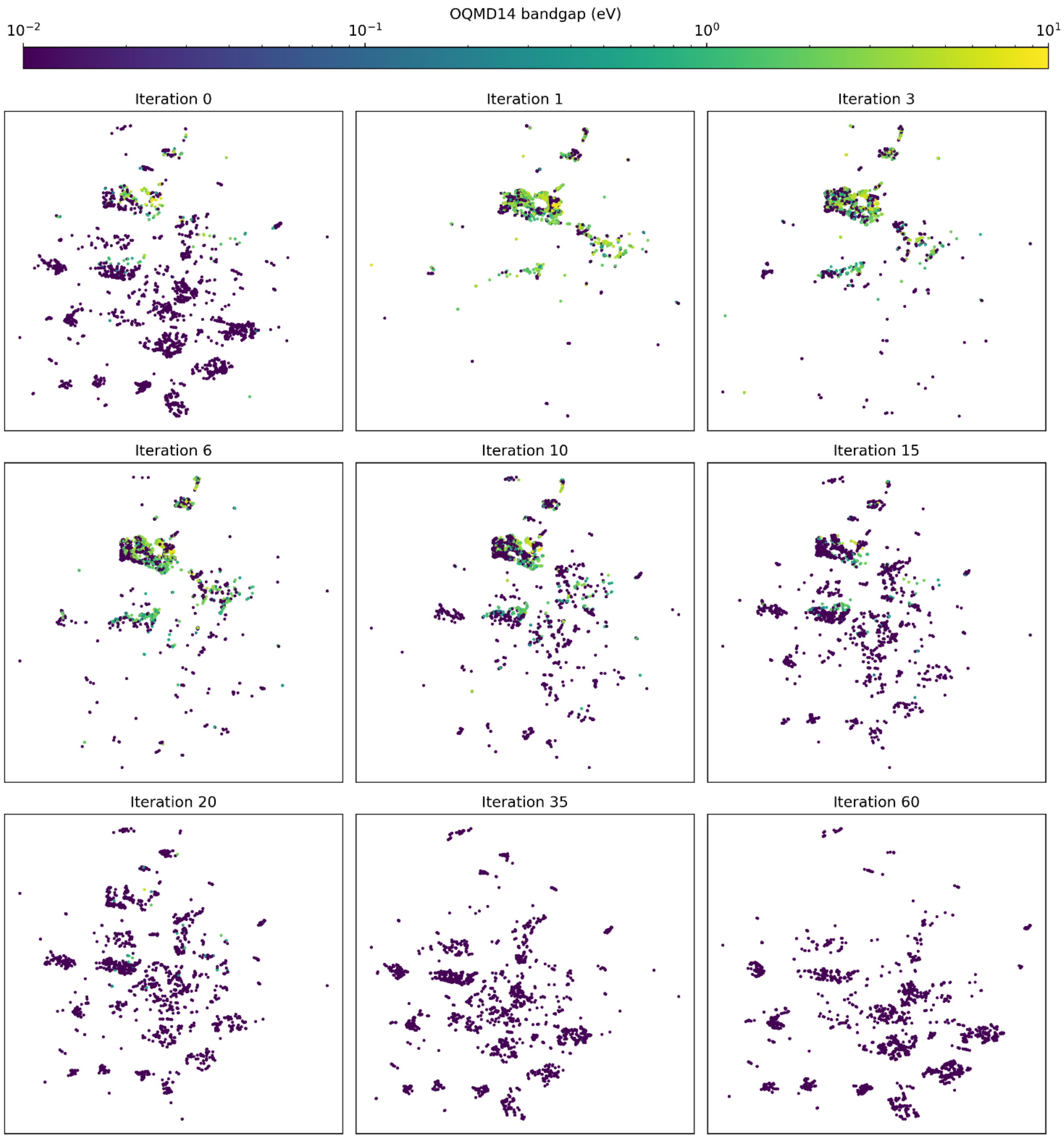

Figure. UMAP projection in the feature space for the samples newly acquired in each active learning iteration step. Each subplot contains the same number of data points equal to a batch size of 1% of the pool. To ensure a consistent UMAP representation across the subplots, the UMAP embeddings are constructed by using all the data in the OQMD14 pool (rather than constructed using the new data in each iteration), and the axis ranges are the same across the subplots.

Comment #19: I find it a bit odd that the hyper parameters are only optimised for the smallest training sets and then kept fixed. This would imply that the learning for larger training sets is suboptimal and could explain why pruning and active learning have such large effects. I would suspect that this is partly an artefact of the lack of hyper parameter optimization. Larger training sets learn less well than they could, which gives the random curve a smaller slope than if the hyper parameters were optimised for every training set size. This means that the intersect of the pruning and active learning curves is pushed to larger training set sizes resulting in seemingly larger data savings.

Response #19: This is a misunderstanding of our hyperparameter tuning procedure. In fact, the hyperparameter set used in this work is optimized for the full training pool. We choose to do so because for ensemble methods, we can use a large number of estimators to saturate the performance without worrying too much about overfitting, though using a too large number of estimators would unnecessarily increase the training cost. Therefore, we optimize the hyperparameters on the full training set and keep the same hyperparameters for the smaller training sets. Contrary to the reviewer's suspicion, the learning might be suboptimal for smaller training sets, implying that the degree of redundancy could be more serious than is revealed.

On the other hand, we did additional verifications to quantify the effects of using the same hyperparameters on the smaller training sets. As mentioned in the original manuscript, *"we verified that the model performance using the optimal hyperparameters tuned from the randomized cross-validation search was close to the one using the chosen hyperparameters"*. Specifically, for each training set size, we tune the hyperparameters using the randomized cross-validation search, and we compare the model performance obtained between the optimal hyperparameters and the ones chosen in the work. The figure below shows the corresponding results for the XGB method.

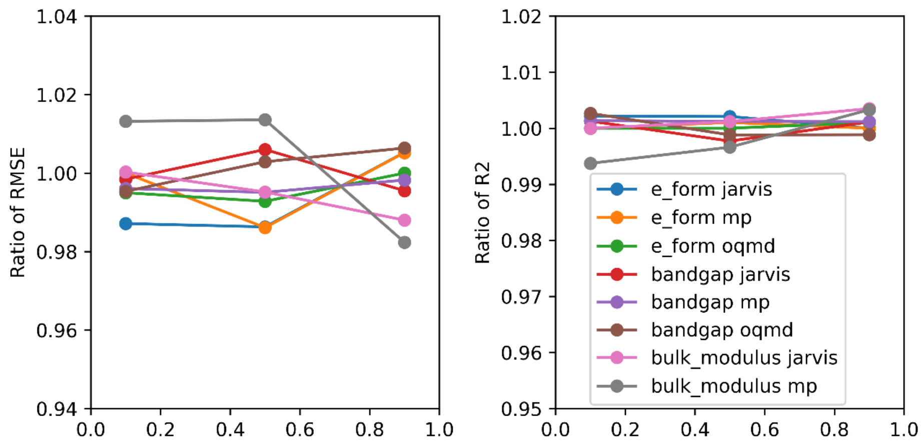

In each subfigure, we show the ratios of the RMSE or R2 obtained using the hyperparameters tuned for the corresponding training set to the error obtained using the hyperparameters. It can be seen that the performance difference is very small (e.g, less than 2% for the RMSE). This is also in accordance with our empirical observation that for ensemble methods, hyperparameters tuned for larger datasets typically work well for smaller datasets.

Comment #20: The description of the pruning algorithm is very brief. More details please. For example, what is meant by “iteration” here? Also, by construction the pruning algorithm seems to require a model that is trained on all data first. So where is the saving?

Response #20: We have rewritten the description of the pruning algorithm with math notations for better clarity in the revised manuscript.

(Method section, lines 656-672) We proposed a pruning algorithm that starts with the full training pool and iteratively reduces the training set size. We denote the full training pool as D_{pool}, the training set at the i-th iteration as D^i_{train}, the unused set as D^i_{unused} ($= D_{pool} - D^i_{train}$), and the trained model as M_i. At the initial iteration ($i = 0$), $D^0_{train} = D_{pool}$ and D^0_{unused} is empty. At each iteration $i > 0$, D^i_{train} and D^i_{unused} are updated as follows: First, a random splitting of D^{i-1}_{train} is performed to obtained two subsets D^i_A (80 % of D^{i-1}_{train}) and D^i_B (20 % of D^{i-1}_{train}). Then, a model M' is trained on D^i_A and then tested on D^i_B. The data in D^i_B with lowest prediction errors (denoted as $D^i_{B,unused}$) are then removed from the training set. Namely, $D^i_{train} = D^{i-1}_{train} - D^i_{B,unused}$, and $D^i_{unused} = D^{i-1}_{unused} + D^i_{B,unused}$. The model M^i trained on D^i_{train} is then used in the performance evaluation on the ID test set, the unused set D^i_{unused} and the OOD test set.

Regarding data saving: First, independent of the data selection algorithms in use, we need to train ML models with different training set sizes, including the largest one, to obtain baselines for comparison to draw conclusion of redundancy and data saving. Second, the pruning algorithm can reveal redundancy of an existing dataset and can therefore lead to data saving from two perspectives: (1) From the data acquisition perspective, the revealed redundancy offers an idea of the amount of data that we could have saved and thus offers feedback to our data acquisition practices. In the active learning context, the pruning algorithm can also be used as a strong baseline to give an idea of the potential room for further data saving and guide the design of active learning methods. (2) From the ML training perspective, while the pruning process does not “save” training data (i.e., reduce the training cost) for *the model that guides the pruning*, the pruned dataset can be used to reduce the training cost for fast development of *other ML models in the future*. This is an important practical consideration as datasets are growing much faster. A closely related example is the work in Ref [21] arXiv:2204.02782 (2022), where different subsets of Open Catalyst 2020 (with over 200 million data points) were used to train ML models and compare their performance, and the randomly subsampled 2 million subset (i.e., less than

1% of the total) was found to be a good choice for accelerating future development cycles of ML models.

Reviewer 3 (Remarks to the Author):

It was a pleasure to read and review the manuscript. Written in well-versed English, it raises an interesting and important question on how much data redundancy is there in the available large comp. material science datasets. The question is important because using redundant data waste computational resources (and heat up our planet on the way ;); more frugal data sets obtained by data pruning will also open research possibilities for smaller research groups that do not have huge computational resources to use a "brute force" approach in mat. sci. computations. The sets of non-redundant data are well known in other data intensive sciences (viz. e.g. < 90% sequence identity sets PDB subsets), so it is high time for material sciences to make similar inventory. The authors offer a working data pruning method to do so.

The authors convincingly demonstrate that indeed large amounts of data can be discarded from existing data sets without impairing efficiency of machine learning processes. The demonstration is done using both in-distribution (ID) and out-of-dist (OOD) data, which is a good control. It is an interesting and not self-evident outcome (at least to me) that reduced data sets are transferable among ML architectures, but not so for different material properties, which opens intriguing questions for future research as to what information faced are important for specific material properties.

The description of the method kindled the idea in my head that information theoretical (Shannon entropy) measures could be used to quantify real information contents in large data sets, and I was pleased to see in the discussion that the authors mention similar ideas and cite Henning 2012 and Zhang 2023 on this matter.

In summary, this is interesting and important paper, with conclusions well supported by the evidence, and presenting ideas important not only for materials research but for all data-driven sciences in general. The data sets that were used for research are well documented and so the similar results could be obtained, following the ideas of the paper, independently. The methodology is sound, and I trust that authors will deposit their data and code as the paper is accepted, which will be of great use for those who would like to apply similar methods to other fields (w.g. bioinformatics and QSAR in chemiformatics could also benefit from such analysis, IMHO).

In my view, the paper does not require any revision and I would love to see it published as presented, just after removing the second "the" on the line 249.

Response #1: We thank the reviewer for the positive evaluation of our work, in particular for the comments related to the importance and novelty of the proposed concepts, and soundness of our methodology, and the robustness of our conclusions. We also thank the reviewer for pointing out the typo which has now been corrected in the revised manuscript.

REVIEWERS' COMMENTS

Reviewer #1 (Remarks to the Author):

My comments have been addressed. I also think that the concerns raised by reviewer 2 are good and helped the authors in streamlining/straightening their story and improve readability. With the additional plots and comments in the supplementary information the comments by reviewer 2 are in my opinion well addressed and make this manuscript a even better one. The comment by reviewer 3 was in-tune with my review.

Point-by-Point Response to the Reviewers' Comments

We would like to thank the editors and reviewers for their careful review of our work and their comments, which are valuable and contribute to further improvement of our work. In what follows, we make point-to-point responses (marked in blue) to all reviewers' comments. We hope our efforts address all the concerns, and the current version satisfies the criteria of *Nature Communications*.

Reviewer #1 (Remarks to the Author):

My comments have been addressed. I also think that the concerns raised by reviewer 2 are good and helped the authors in streamlining/straightening their story and improve readability. With the additional plots and comments in the supplementary information the comments by reviewer 2 are in my opinion well addressed and make this manuscript a even better one. The comment by reviewer 3 was in-tune with my review.

Response: We thank the reviewer for the positive evaluation of our work.
